# Supplementary material for: HER2-targeting antibody drug conjugate FS-1502 in HER2-expressing metastatic breast cancer: a phase 1a/1b trial
Source: Nat Commun. 2024 Jun 17;15:5158. doi: 10.1038/s41467-024-48798-w (PMC11183070; doi:10.1038/s41467-024-48798-w)
Supplement: Supplementary file 1 — Supplementary Information [file 41467_2024_48798_MOESM1_ESM.docx]

**Table of Contents**

[Supplementary Note 1. A list of the ethic committee that approved the study protocol 2](#_Toc165904939)

[Supplementary Note 2. Eligibility criteria 3](#_Toc165904940)

[Supplementary Note 3. Definition of dose limiting toxicity (DLT) 7](#_Toc165904941)

[Supplementary Note 4. Dose modification rules 8](#_Toc165904942)

[Supplementary Table 1. Demographic and baseline characteristics 9](#_Toc165904943)

[Supplementary Table 2. Summary of adverse events (safety set) 10](#_Toc165904944)

[Supplementary Table 3. Summary of study drug-related AEs ≥15% at each CTCAE grade (safety set) 11](#_Toc165904945)

[Supplementary Table 4. Pharmacokinetic parameters of FS-1502 (PK Parameter Analysis Set) 12](#_Toc165904946)

[Supplementary Table 5. Pharmacokinetic parameters of total antibody (PK Parameter Analysis Set) 13](#_Toc165904947)

[Supplementary Table 6. Pharmacokinetic parameters of unconjugated MMAF (PK Parameter Analysis Set) 14](#_Toc165904948)

[Supplementary Figure 1. Forest plot of subgroup analysis in HER2-positive breast cancer patients 15](#_Toc165904949)

[Supplementary Figure 2. Serum concentration of FS-1502 at (A) Cycle 1 and (B) Cycle 3 (PK Concentration Analysis Set) 16](#_Toc165904950)

[Supplementary Figure 3. Serum concentration of total antibody at (A) Cycle 1 and (B) Cycle 3 (PK Concentration Analysis Set) 17](#_Toc165904951)

[Supplementary Figure 4. Serum concentration of unconjugated MMAF at (A) Cycle 1 and (B) Cycle 3 (PK Concentration Analysis Set) 18](#_Toc165904952)

[Supplementary Note 5. Study protocol and amendments 19](#_Toc165904953)

## Supplementary Note 1. A list of the ethic committee that approved the study protocol

|  | **Name** |
| --- | --- |
| **1** | National Cancer Center/Cancer Hospital Chinese Academy of Medical Sciences and Peking Union Medical College, Beijing, China |
| **2** | Sir Run Run Shaw Hospital, Zhejiang university School of Medicine, Zhejiang, China |
| **3** | Jilin Cancer Hospital, Jilin, China |
| **4** | The Fourth Hospital of Hebei Medical University, Hebei, China |
| **5** | Cancer Hospital Chinese Academy of Medical Sciences, Shenzhen Center, Shenzhen, China |
| **6** | Henan Cancer Hospital, Zhengzhou, China |
| **7** | Tianjin Medical University Cancer Institute and Hospital, Tianjin, China |
| **8** | Sun Yat-sen University Cancer Center, Guangzhou, China |
| **9** | Meizhou People's Hospital, Meizhou, China |

## Supplementary Note 2. Eligibility criteria

**Inclusion Criteria:**

1. Age ≥18 years at the time of study registration (men and women eligible).
2. Phase 1a dose-escalation study: Patients with HER2 expressed advanced malignant solid tumor had failed to standard therapy (including surgery, chemotherapy, radiotherapy, or biotherapy), or cannot receive standard therapy, or no available standard therapy.
   1. HER2 high expression: IHC3+, IHC2+/FISH+, or FISH+
   2. HER2 low expression: IHC1+, IHC2+/FISH-

Phase 1b dose-expansion study: Histologically or cytologically confirmed HER2 high expression breast cancer patients who had failed to prior anti-HER2 therapy and had received at least 2 treatment lines for advanced breast cancer (ABC).

- 1. HER2 high expression: IHC3+, IHC2+/FISH+, or FISH+.
  2. Had failed to prior anti-HER2 therapy and had received at least 2 treatment lines for ABC. Disease progression occurred during treatment or within 12 months after the end of postoperative adjuvant therapy could be counted as one line of treatment.
  3. Provided evidence of disease progression or intolerable toxicity as confirmed by the investigator or medical history recorded prior to enrollment.
  4. Patients could provide local laboratory HER2 examination written report for enrollment. Without HER2 examination report, patients must provide sufficient paraffin sections or fresh tumor tissue samples for confirmation by study center or central laboratory.

1. The ECOG performance status must be 0 or 1.
2. Expected survival for at least 12 weeks.
3. Has adequate organ and bone marrow function: absolute neutrophil count (ANC) ≥ 1.5x10^9^/L; hemoglobin ≥ 90g/L (without red blood cell infusion within 14 days); platelet count ≥ 100x10^9^/L; total bilirubin ≤ 1.5x upper limit normal (ULN), or ≤ 3x ULN if with Gilbert syndrome; aspartate aminotransferase (AST) and alanine aminotransferase (ALT) ≤ 2.5x ULN; AST and ALT ≤ 5x ULN if liver metastasis; serum creatinine < 1.5x ULN and creatinine clearance ≥ 60mL/min (Cockroft-Gault formula calculation); serum potassium ≥ 3.5mmol/L; albumin ≥ 3g/dL; left ventricular ejection fraction (LVEF) >50%; urine protein ≤ 1+, or 24-hour urine protein quantification < 1.0g.
4. Had at least one measurable non-intracranial lesion by RECIST version 1.1.
5. Male or female patients with fertility must agree to use effective contraceptive methods during the study period and within 30 days of the last dose of study treatment, such as dual barrier contraceptive methods, condoms, oral or injectable contraceptives, and intrauterine devices.
6. Ability to understand and voluntarily sign the written informed consent.

**Exclusion Criteria:**

1. Patients who had received chemotherapy, targeted therapy, or radiotherapy, etc., 14 days or within 5 half-lives periods prior to the study treatment, whichever is shorter; had received major surgery, tumor immunotherapy, or antitumor monoclonal antibody therapy within 4 weeks prior to the study treatment.
2. Patients who had participated in other clinical trials 4 weeks or within 5 half-lives periods prior to the study treatment, whichever is shorter; had received similar treatments.
3. Patients who had received anti-HER2 ADC therapy other than T-DM1.
4. Uncontrolled central nervous system metastasis or injury. Patients who met listed conditions were allowed for enrollment:
   1. Had received local therapy and had a stable disease for more than 2 months.
   2. No need for corticosteroids therapy or other treatments for dehydration, and with a stable dosage of antiepileptic drugs (if applicable).
   3. No meningeal metastasis.
5. Patients with uncontrolled diabetes mellitus (Patients who were receiving a stable insulin regimen or a stable hypoglycemic regimen, and with a specialist-evaluated good glycemic control were allowed for enrollment).
6. Had not recovered from previous antitumor treatment-related toxicity (> NCI-CTCAE 5.0 Grade 2); the neurotoxicity of patients who had previously received chemotherapy needed to be restored to NCI-CTCAE 5.0 Grade 2 or below.
7. Had keratopathy (except for mild punctate keratopathy).
8. Patients who had received drugs that prolong the QTc interval (mainly Ia, Ic, and III antiarrhythmic drugs) or patients with risk factors for QTc interval prolongation, such as uncorrectable hypokalemia, hereditary long QT syndrome. Drugs that potentially prolong the QTc interval can be found at <https://crediblemeds.org/index.php/tools/pdfdownload?f=cql_en>.
9. Cardiac function and disease met one of the following conditions:
   1. Three 12-lead electrocardiogram (ECG) measurements at the study center during the screening period. Calculate the average of three measurements based on the QTc formula, QTc > 470 milliseconds.
   2. New York Heart Association (NYHA) graded ≥3 congestive heart failure.
   3. Clinically significant arrhythmias, including but not limited to complete left bundle branch conduction abnormality, and second-degree atrioventricular block.
10. Pregnant or lactating woman.
11. Allergic to any excipients of FS-1502.
12. Clinically significant active bacterial, fungal or viral infections, including hepatitis B (hepatitis B virus surface antigen positive and hepatitis B virus DNA over 1000 IU/ml) or hepatitis C (hepatitis C virus RNA positive), human immunodeficiency virus infection (HIV positive).
13. Any other disease or condition of clinical significance (eg, active or uncontrollable infection, etc.) considered by investigator that may affect protocol compliance or affect ICF signature.

## Supplementary Note 3. Definition of dose limiting toxicity (DLT)

DLT was defined as a dose-limiting toxic event that occurred during the observation period of DLT, which was at least probably related to FS-1502 by the discretion of investigator and/or sponsor. DLT was graded per NCI-CTCAE version 5.0. Any of the following AE or laboratory abnormality was considered as a DLT.

1. Grade 4 hematological toxicity as below:
   1. Grade 4 neutropenia lasting ≥3 days after symptomatic treatment.
   2. Grade 4 febrile neutropenia (fever ≥38.0 °C lasting for one hour or ≥38.3 °C).
   3. Grade 4 thrombocytopenia or grade 3 thrombocytopenia with obvious hemorrhage.
2. Non-hematological toxicity grade ≥3 (not resulted from disease progression)
   1. Any grade ≥3 ALT, AST, or total bilirubin increases.
   2. Grade 3 nausea, vomiting, diarrhea, and electrolyte disturbance (with the best supportive treatment lasting for 3 days).
   3. QTc interval ≥501 ms (the average of at least 2 ECG measurements) or QTc interval prolonged ≥60 ms from baseline.
   4. Grade ≥2 interstitial pneumonia.
   5. Grade ≥3 nephrotoxicity not recovered within 7 days, including nephrogenic hematuria, proteinuria, and decreased creatinine clearance, etc.
   6. Grade ≥3 increased amylase or grade 2 pancreatitis.
3. Others: in the opinion of the investigator or sponsor, a toxic event that required dose adjustment, or dose interruption, or was clinically unacceptable.

## Supplementary Note 4. Dose modification rules

If a patient experienced an NCI CTCAE v5.0 Grade 3 or higher and/or unacceptable toxic event, the study drug was discontinued, and supportive care was given according to local routine. If the toxicity recovered to an NCI CTCAE grade 1 or less within 2 cycles and the patient did not progress, the original dose of the study drug could be continued or the dose could be reduced after discussion between the investigator and the sponsor. If the toxicity recovered to ≤ NCI CTCAE Grade 1 after 2 cycles, the investigator discussed with the sponsor's medical monitoring, and the decision to continue the medication was made based on the patient's clinical benefit. If the patient recovered within 2 cycles and did not progress at the time of recurrence of a Grade 3 toxic event, the study drug could be re-administered by one dose reduction. At the third occurrence of a Grade 3 toxicity event, if the patient recovered within 2 cycles and did not progress, then the dose was reduced by one additional dose and re-administered. Patients were allowed a maximum of 3 dose modifications after which no further dose modifications were allowed, but a dose interruption was allowed. Dose reduction was not allowed during the DLT observation period during the dose escalation phase. Refer to Table 6-1 for the guidelines for dose reduction.

**Table 6-1 FS-1502 Dose Reduction Principles**

| **Start Dose** | **Dose Grade 1** | **Dose Grade 2** | **Dose Grade 3** |
| --- | --- | --- | --- |
| 2.3 mg/kg | 1.7 mg/kg | 1.3 mg/kg | 1.0 mg/kg |

## Supplementary Table 1. Demographic and baseline characteristics

| Dose | **0.1 mg/kg** | **0.2 mg/kg** | **0.4 mg/kg** | **0.6 mg/kg** | **0.8 mg/kg** | **1.0 mg/kg** | | **1.3 mg/kg** | | **1.7 mg/kg** | | **2.3 mg/kg** | **3.0 mg/kg** | **3.5 mg/kg** | **Total** |
| --- | --- | --- | --- | --- | --- | --- | --- | --- | --- | --- | --- | --- | --- | --- | --- |
| Dose frequency | Q4W  (*n*=3) | Q4W  (*n*=4) | Q4W  (*n*=5) | Q4W  (*n*=7) | Q4W  (*n*=4) | Q4W  (*n*=6) | Q3W  (*n*=3) | Q4W  (*n*=6) | Q3W  (*n*=4) | Q4W  (*n*=5) | Q3W  (*n*=4) | Q3W  (*n*=89) | Q3W  (*n*=9) | Q3W  (*n*=1) | (*n*=150) |
| Median age (range), yr | 42.0  (40-54) | 41.5  (36-60) | 51.0  (43-66) | 43.0  (35-57) | 49.5  (38-67) | 52.5  (33-71) | 42.0  (39-63) | 55.0  (36-64) | 43.5  (29-56) | 54.0  (38-74) | 49.5  (39-61) | 52.0  (27-76) | 57.0  (44-66) | 47.0  (47-47) | 52.0  (27-76) |
| ECOG PS, *n* (%) |  |  |  |  |  |  |  |  |  |  |  |  |  |  |  |
| 0 | 3 (100) | 2 (50.0) | 3 (60.0) | 5 (71.4) | 4 (100) | 4 (66.7) | 3 (100) | 2 (33.3) | 2 (50.0) | 1 (20.0) | 4 (100) | 41 (46.1) | 1 (11.1) | 1 (100) | 76 (50.7) |
| 1 | 0 | 2 (50.0) | 2 (40.0) | 2 (28.6) | 0 | 2 (33.3) | 0 | 4 (66.7) | 2 (50.0) | 4 (80.0) | 0 | 48 (53.9) | 8 (88.9) | 0 | 74 (49.3) |
| Tumor type, *n* (%) |  |  |  |  |  |  |  |  |  |  |  |  |  |  |  |
| Breast cancer | 3 (100) | 4 (100) | 5 (100) | 7 (100) | 4 (100) | 6 (100) | 3 (100) | 6 (100) | 4 (100) | 5 (100) | 4 (100) | 87 (97.8) | 6 (66.7) | 1 (100) | 145 (96.7) |
| Others |  |  |  |  |  |  |  |  |  |  |  | 2 (2.2) | 3 (33.3) | 0 | 5 (3.3) |
| Metastatic site, *n* (%) |  |  |  |  |  |  |  |  |  |  |  |  |  |  |  |
| Bone | 3 (100) | 2 (50.0) | 0 | 4 (57.1) | 1 (25.0) | 0 | 1 (33.3) | 3 (50.0) | 2 (50.0) | 2 (40.0) | 1 (25.0) | 39 (43.8) | 5 (55.6) | 0 | 63 (42.0) |
| Liver | 3 (100) | 1 (25.0) | 1 (20.0) | 2 (28.6) | 2 (50.0) | 1 (16.7) | 0 | 2 (33.3) | 2 (50.0) | 2 (40.0) | 2 (50.0) | 42 (47.2) | 3 (33.3) | 0 | 63 (42.0) |
| Lung | 1 (33.3) | 1 (25.0) | 3 (60.0) | 4 (57.1) | 3 (75.0) | 3 (50.0) | 2 (66.7) | 3 (50.0) | 3 (75.0) | 4 (80.0) | 3 (75.0) | 46 (51.7) | 4 (44.4) | 1 (100) | 81 (54.0) |
| Brain | 1 (33.3) | 1 (25.0) | 0 | 0 | 0 | 0 | 0 | 0 | 0 | 0 | 0 | 7 (7.9) | 2 (22.2) | 0 | 11 (7.3) |
| Lymph nodes | 2 (66.7) | 3 (75.0) | 5 (100) | 4 (57.1) | 4 (100) | 5 (83.3) | 1 (33.3) | 4 (66.7) | 2 (50.0) | 4 (80.0) | 2 (50.0) | 49 (55.1) | 6 (66.7) | 0 | 91 (60.7) |
| Other | 0 | 2 (50.0) | 2 (40.0) | 4 (57.1) | 0 | 6 (100) | 0 | 1 (16.7) | 2 (50.0) | 1 (20.0) | 0 | 26 (29.2) | 5 (55.6) | 1 (100) | 51 (34.0) |
| HER2 expression status, *n* (%) |  |  |  |  |  |  |  |  |  |  |  |  |  |  |  |
| *HER2*-low expression (HER2 1+ or HER2 2+/FISH-) | 0 | 3 (75.0) | 2 (40.0) | 2 (28.6) | 1 (25.0) | 1 (16.7) | 2 (66.7) | 0 | 2 (50.0) | 2 (40.0) | 0 | 16 (18.0) | 1 (11.1) | 0 | 32 (21.3) |
| *HER2*-positive (HER2 3+ or HER2 2+/FISH+) | 3 (100) | 1 (25.0) | 3 (60.0) | 5 (71.4) | 3 (75.0) | 5 (83.3) | 1 (33.3) | 6 (100) | 1 (25.0) | 3 (60.0) | 4 (100) | 71 (81.6) | 5 (55.6) | 1 (100) | 112 (74.7) |
| HER2 2+ | 0 | 0 | 0 | 0 | 0 | 0 | 0 | 0 | 0 | 0 | 0 | 1 (1.1) | 1 (11.1) | 0 | 2 (1.3) |
| HER2 3+ | 0 | 0 | 0 | 0 | 0 | 0 | 0 | 0 | 0 | 0 | 0 | 1 (1.1) | 2 (22.2) | 0 | 3 (2.0) |
| History of prior treatment lines, median (min–max) | 3.0 (1-5) | 4.0 (2-6) | 3.0 (1-6) | 4.0 (2-6) | 2.5 (1-7) | 4.5 (3-8) | 1.0 (1-3) | 1.5 (1-6) | 3.5 (3-6) | 2.0 (1-4) | 2.0 (2-3) | 3.0 (1-16) | 2.0 (1-10) | 2.0 (2-2) | 3.0 (1-16) |
| History of anti-HER2 therapy for HER2 positive breast cancer pts, *n* (%) | 3 (100) | 1 (25.0) | 3 (60.0) | 5 (71.4) | 3 (75.0) | 5 (83.3) | 1 (33.3) | 6 (100) | 1 (25.0) | 3 (60.0) | 4 (100) | 71 (79.8) | 5 (55.6) | 1 (100) | 112 (74.7) |
| Trastuzumab^a^ | 2 (66.7) | 1 (100) | 3 (100) | 5 (100) | 3 (100) | 5 (100) | 1 (100) | 4 (66.7) | 1 (100) | 3 (100) | 4 (100) | 69 (97.2) | 5 (100) | 1 (100) | 107 (95.5) |
| Pyrotinib^a^ | 2 (66.7) | 0 | 0 | 3 (60.0) | 2 (66.7) | 5 (100) | 0 | 1 (16.7) | 1 (100) | 1 (33.3) | 3 (75.0) | 54 (76.1) | 3 (60.0) | 0 | 75 (67.0) |
| Pertuzumab^a^ | 0 | 0 | 0 | 1 (20.0) | 1 (33.3) | 2 (40.0) | 0 | 2 (33.3) | 0 | 1 (33.3) | 0 | 28 (39.4) | 2 (40.0) | 1 (100) | 38 (33.9) |
| Lapatinib^a^ | 2 (66.7) | 0 | 3 (100) | 0 | 2 (66.7) | 3 (60.0) | 0 | 0 | 1 (100) | 0 | 1 (25.0) | 14 (19.7) | 2 (40.0) | 0 | 28 (25.0) |
| Inetetamab^a^ | 0 | 0 | 0 | 0 | 0 | 0 | 0 | 0 | 0 | 0 | 0 | 12 (16.9) | 0 | 1 (100) | 13 (11.6) |
| Other^a^ | 0 | 0 | 1 (33.3) | 0 | 0 | 0 | 0 | 1 (16.7) | 0 | 0 | 0 | 6 (8.5) | 0 | 0 | 8 (7.1) |
| T-DM1^a^ | 0 | 0 | 1 (33.3) | 0 | 0 | 1 (20.0) | 0 | 0 | 0 | 0 | 0 | 4 (5.6) | 0 | 0 | 6 (5.4) |

^a^The denominator is the number of patients with *HER2*-positive breast cancer in each dose group.

## Supplementary Table 2. Summary of adverse events (safety set)

| Dose | **0.1 mg/kg** | **0.2 mg/kg** | **0.4 mg/kg** | **0.6 mg/kg** | **0.8 mg/kg** | **1.0 mg/kg** | | **1.3 mg/kg** | | **1.7 mg/kg** | | **2.3 mg/kg** | **3.0 mg/kg** | **3.5 mg/kg** | **Total** |
| --- | --- | --- | --- | --- | --- | --- | --- | --- | --- | --- | --- | --- | --- | --- | --- |
| Dose frequency | Q4W  (*n*=3) | Q4W  (*n*=4) | Q4W  (*n*=5) | Q4W  (*n*=7) | Q4W  (*n*=4) | Q4W  (*n*=6) | Q3W  (*n*=3) | Q4W  (*n*=6) | Q3W  (*n*=4) | Q4W  (*n*=5) | Q3W  (*n*=4) | Q3W  (*n*=89) | Q3W  (*n*=9) | Q3W  (*n*=1) | (*n*=150) |
| ***n* (%)** |  |  |  |  |  |  |  |  |  |  |  |  |  |  |  |
| Any TEAEs | 3 (100) | 4 (100) | 5 (100) | 7 (100) | 4 (100) | 6 (100) | 3 (100) | 6 (100) | 4 (100) | 5 (100) | 4 (100) | 85 (95.5) | 9 (100) | 1 (100) | 146 (97.3) |
| TEAEs of CTCAE grade ≥3 | 1 (33.3) | 2 (50.0) | 1 (20.0) | 1 (14.3) | 0 | 2 (33.3) | 0 | 1 (16.7) | 2 (50.0) | 2 (40.0) | 2 (50.0) | 36 (40.4) | 8 (88.9) | 1 (100) | 59 (39.3) |
| SAEs | 0 | 1 (25.0) | 0 | 0 | 0 | 0 | 0 | 0 | 0 | 0 | 2 (50.0) | 15 (16.9) | 3 (33.3) | 0 | 21 (14.0) |
| Study drug-related TEAEs | 2 (66.7) | 4 (100) | 5 (100) | 7 (100) | 4 (100) | 4 (66.7) | 3 (100) | 5 (83.3) | 4 (100) | 5 (100) | 4 (100) | 85 (95.5) | 9 (100) | 1 (100) | 142 (94.7) |
| Study drug-related TEAEs of CTCAE grade ≥3 | 1 (33.3) | 2 (50.0) | 1 (20.0) | 1 (14.3% | 0 | 1 (16.7) | 0 | 0 | 2 (50.0) | 1 (20.0) | 2 (50.0) | 32 (36.0) | 7 (77.8) | 1 (100) | 51 (34.0) |
| Study drug-related SAEs | 0 | 1 (25.0) | 0 | 0 | 0 | 0 | 0 | 0 | 0 | 0 | 0 | 10 (11.2) | 3 (33.3) | 0 | 14 (9.3) |
| TEAE leading to drug interruption | 0 | 1 (25.0) | 0 | 0 | 0 | 0 | 0 | 0 | 1 (25.0) | 1 (20.0) | 3 (75.0) | 32 (36.0) | 7 (77.8) | 1 (100) | 46 (30.7) |
| TEAE leading to drug discontinuation | 0 | 0 | 0 | 0 | 0 | 0 | 1 (33.3) | 0 | 0 | 0 | 0 | 5 (5.6) | 1 (11.1) | 0 | 7 (4.7) |
| TEAE leading to drug reduction | 0 | 0 | 0 | 0 | 0 | 0 | 0 | 1 (16.7) | 1 (25.0) | 2 (40.0) | 2 (50.0) | 27 (30.3) | 3 (33.3) | 1 (100) | 37 (24.7) |
| TEAE leading to death | 0 | 0 | 0 | 0 | 0 | 0 | 0 | 0 | 0 | 0 | 0 | 3 (3.4) | 1 (11.1) | 0 | 4 (2.7) |
| **Study drug–related TEAEs ≥15% for all patients by preferred term** | | | | | | | | | | | | | | | |
| AST increased | 1 (33.3) | 2 (50.0) | 2 (40.0) | 3 (42.9) | 2 (50.0) | 2 (33.3) | 2 (66.7) | 1 (16.7) | 2 (50.0) | 5 (100) | 2 (50.0) | 66 (74.2) | 9 (100) | 1 (100) | 100 (66.7) |
| Hypokalemia | 0 | 0 | 0 | 1 (14.3) | 0 | 0 | 0 | 2 (33.3) | 3 (75.0) | 2 (40.0) | 3 (75.0) | 59 (66.3) | 6 (66.7) | 1 (100) | 77 (51.3) |
| ALT increased | 1 (33.3) | 2 (50.0) | 2 (40.0) | 3 (42.9) | 2 (50.0) | 2 (33.3) | 1 (33.3) | 1 (16.7) | 2 (50.0) | 4 (80.0) | 3 (75.0) | 39 (43.8) | 4 (44.4) | 0 | 66 (44.0) |
| Platelet count decreased | 0 | 2 (50.0) | 0 | 0 | 0 | 1 (16.7) | 0 | 1 (16.7) | 2 (50.0) | 2 (40.0) | 2 (50.0) | 31 (34.8) | 9 (100) | 1 (100) | 51 (34.0) |
| Proteinuria | 1 (33.3) | 0 | 0 | 0 | 0 | 1 (16.7) | 1 (33.3) | 0 | 0 | 0 | 3 (75.0) | 36 (40.4) | 8 (88.9) | 1 (100) | 51 (34.0) |
| Dry mouth | 0 | 0 | 0 | 1 (14.3) | 1 (25.0) | 1 (16.7) | 1 (33.3) | 1 (16.7) | 2 (50.0) | 1 (20.0) | 2 (50.0) | 32 (36.0) | 7 (77.8) | 1 (100) | 50 (33.3) |
| Anemia | 1 (33.3) | 1 (25.0) | 0 | 2 (28.6) | 0 | 2 (33.3) | 0 | 2 (33.3) | 1 (25.0) | 0 | 1 (25.0) | 24 (27.0) | 5 (55.6) | 1 (100) | 40 (26.7) |
| Blood lactate dehydrogenase increased | 0 | 1 (25.0) | 1 (20.0) | 0 | 1 (25.0) | 1 (16.7) | 2 (66.7) | 0 | 1 (25.0) | 2 (40.0) | 0 | 29 (32.6) | 0 | 1 (100) | 39 (26.0) |
| Dry eye | 0 | 0 | 0 | 0 | 0 | 3 (50.0) | 1 (33.3) | 1 (16.7) | 1 (25.0) | 1 (20.0) | 3 (75.0) | 19 (21.3) | 7 (77.8) | 0 | 36 (24.0) |
| Hyperuricemia | 0 | 2 (50.0) | 1 (20.0) | 1 (14.3) | 0 | 2 (33.3) | 3 (100) | 1 (16.7) | 2 (50.0) | 0 | 0 | 21 (23.6) | 2 (22.2) | 1 (100) | 36 (24.0) |
| Alopecia | 0 | 0 | 0 | 2 (28.6) | 0 | 3 (50.0) | 2 (66.7) | 2 (33.3) | 1 (25.0) | 1 (20.0) | 2 (50.0) | 18 (20.2) | 2 (22.2) | 1 (100) | 34 (22.7) |
| Hypercholesterolemia | 0 | 0 | 1 (20.0) | 1 (14.3) | 3 (75.0) | 1 (16.7) | 1 (33.3) | 0 | 0 | 1 (20.0) | 1 (25.0) | 20 (22.5) | 2 (22.2) | 0 | 31 (20.7) |
| Decreased appetite | 0 | 0 | 0 | 1 (14.3) | 0 | 0 | 0 | 1 (16.7) | 1 (25.0) | 3 (60.0) | 0 | 19 (21.3) | 6 (66.7) | 0 | 31 (20.7) |
| Gamma-glutamyltransferase increased | 1 (33.3) | 1 (25.0) | 1 (20.0) | 1 (14.3) | 2 (50.0) | 1 (16.7) | 1 (33.3) | 0 | 0 | 3 (60.0) | 0 | 15 (16.9) | 1 (11.1) | 1 (100) | 28 (18.7) |
| Weight decreased | 0 | 0 | 0 | 0 | 0 | 1 (16.7) | 1 (33.3) | 2 (33.3) | 0 | 2 (40.0) | 2 (50.0) | 16 (18.0) | 2 (44.4) | 0 | 28 (18.7) |
| Keratitis | 0 | 0 | 0 | 0 | 0 | 0 | 0 | 0 | 1 (25.0) | 0 | 1 (25.0) | 24 (27.0) | 0 | 1 (100) | 27 (18.0) |
| White blood cell count decreased | 1 (33.3) | 1 (25.0) | 2 (40.0) | 2 (28.6) | 0 | 0 | 0 | 4 (66.7) | 0 | 2 (40.0) | 1 (25.0) | 13 (14.6) | 1 (11.1) | 0 | 27 (18.0) |
| Amylase increased | 0 | 1 (25.0) | 0 | 0 | 0 | 0 | 0 | 0 | 1 (25.0) | 0 | 0 | 18 (20.2) | 5 (55.6) | 0 | 25 (16.7) |
| Hypertriglyceridemia | 0 | 0 | 0 | 2 (28.6) | 2 (50.0) | 1 (16.7) | 1 (33.3) | 0 | 0 | 0 | 0 | 18 (20.2) | 0 | 1 (100) | 25 (16.7) |
| Fatigue | 0 | 2 (50.0) | 0 | 1 (14.3) | 1 (25.0) | 2 (33.3) | 0 | 1 (16.7) | 2 (50.0) | 1 (20.0) | 1 (25.0) | 8 (9.0) | 5 (55/6) | 0 | 24 (16.0) |
| Hypoalbuminemia | 0 | 0 | 0 | 0 | 0 | 1 (16.7) | 0 | 0 | 1 (25.0) | 0 | 1 (25.0) | 19 (21.3) | 1 (11.1) | 0 | 23 (15.3) |
| **CTCAE grade ≥3 study drug related TEAEs ≥2% for all patients by preferred term** | | | | | | | | | | | | | | | |
| Hypokalemia | 0 | 0 | 0 | 0 | 0 | 0 | 0 | 0 | 0 | 1 (20.0) | 2 (50.0) | 15 (16.9) | 5 (55.6) | 0 | 23 (15.3) |
| Platelet count decreased | 0 | 2 (50.0) | 0 | 0 | 0 | 0 | 0 | 0 | 0 | 0 | 0 | 7 (7.9) | 2 (22.2) | 1 (100) | 12 (8.0) |
| Anemia | 0 | 1 (25.0) | 0 | 1 (14.3) | 0 | 0 | 0 | 0 | 1 (25.0) | 0 | 0 | 3 (3.4) | 1 (11.1) | 0 | 7 (4.7) |
| Electrocardiogram QT prolonged | 0 | 0 | 0 | 0 | 0 | 0 | 0 | 0 | 0 | 1 (20.0) | 0 | 3 (3.4) | 0 | 0 | 4 (2.7) |
| Neutrophil count decreased | 0 | 0 | 0 | 0 | 0 | 0 | 0 | 0 | 0 | 0 | 0 | 4 (4.5) | 0 | 0 | 4 (2.7) |
| Gamma-glutamyltransferase increased | 1 (33.3) | 0 | 0 | 0 | 0 | 1 (16.7) | 0 | 0 | 0 | 0 | 0 | 1 (1.1) | 0 | 0 | 3 (2.0) |
| Pneumonitis | 0 | 0 | 0 | 0 | 0 | 0 | 0 | 0 | 0 | 0 | 0 | 1 (1.1) | 2 (22.2) | 0 | 3 (2.0) |

AST, aspartate aminotransferase; ALT, alanine aminotransferase; SAE, serious adverse events; TEAEs: treatment-emergent adverse events.

## Supplementary Table 3. Summary of study drug-related AEs ≥15% at each CTCAE grade (safety set)

| Dose | **2.3 mg/kg**  Q3W  (*n*=89) | **Grade 1** | **Grade 2** | **Grade 3** | **Grade 4** | **Total**  (*n*=150) | **Grade 1** | **Grade 2** | **Grade 3** | **Grade 4** |
| --- | --- | --- | --- | --- | --- | --- | --- | --- | --- | --- |
| AST increased | 66 (74.2) | 58 (65.2%) | 9 (10.1%) | 1 (1.1%) | 0 | 100 (66.7) | 89 (59.3%) | 15 (10.0%) | 1 (0.7%) | 0 |
| Hypokalemia | 59 (66.3) | 40 (44.9%) | 18 (20.2%) | 14 (15.7%) | 1 (1.1%) | 77 (51.3) | 53 (35.3%) | 26 (17.3%) | 22 (14.7%) | 1 (0.7%) |
| ALT increased | 39 (43.8) | 37 (41.6%) | 4 (4.5%) | 1 (1.1%) | 0 | 66 (44.0) | 63 (42.0%) | 6 (4.0%) | 2 (1.3%) | 0 |
| Proteinuria | 36 (40.4) | 30 (33.7%) | 8 (9.0%) | 0 | 0 | 51 (34.0) | 37 (24.7%) | 16 (10.7%) | 0 | 0 |
| Dry mouth | 32 (36.0) | 24 (27.0%) | 6 (6.7%) | 2 (2.2%) | 0 | 50 (33.3) | 33 (22.0%) | 15 (10.0%) | 2 (1.3%) | 0 |
| Platelet count decreased | 31 (34.8) | 21 (23.6%) | 11 (12.4%) | 6 (6.7%) | 1 (1.1%) | 51 (34.0) | 34 (22.7%) | 22 (14.7%) | 11 (7.3%) | 2 (1.3%) |
| Blood lactate dehydrogenase increased | 29 (32.6) | 29 (32.6%) | 0 | 0 | 0 | 39 (26.0) | 39 (26.0%) | 0 | 0 | 0 |
| Anemia | 24 (27.0) | 17 (19.1%) | 4 (4.5%) | 2 (2.2%) | 1 (1.1%) | 40 (26.7) | 27 (18.0%) | 7 (4.7%) | 6 (4.0%) | 1 (0.7%) |
| Keratitis | 24 (27.0) | 13 (14.6%) | 12 (13.5%) | 0 | 0 | 27 (18.0) | 15 (10.0%) | 13 (8.7%) | 0 | 0 |
| Hyperuricemia | 21 (23.6) | 21 (23.6%) | 1 (1.1%) | 0 | 0 | 36 (24.0) | 36 (24.0%) | 1 (0.7%) | 0 | 0 |
| Hypercholesterolemia | 20 (22.5) | 16 (18.0%) | 6 (6.7%) | 1 (1.1%) | 0 | 31 (20.7) | 27 (18.0%) | 6 (4.0%) | 1 (0.7%) | 0 |
| Dry eye | 19 (21.3) | 16 (18.0%) | 1 (1.1%) | 2 (2.2%) | 0 | 36 (24.0) | 24 (16.0%) | 10 (6.7%) | 2 (1.3%) | 0 |
| Decreased appetite | 19 (21.3) | 13 (14.6%) | 6 (6.7%) | 0 | 0 | 31 (20.7) | 21 (14.0%) | 11 (7.3%) | 0 | 0 |
| Hypoalbuminemia | 19 (21.3) | 0 | 1 (1.1%) | 0 | 0 | 23 (15.3) | 1 (0.7%) | 2 (1.3%) | 0 | 0 |
| Alopecia | 18 (20.2) | 9 (10.1%) | 9 (10.1%) | 0 | 0 | 34 (22.7) | 19 (12.7%) | 15 (10.0%) | 0 | 0 |
| Amylase increased | 18 (20.2) | 14 (15.7%) | 5 (5.6%) | 0 | 0 | 25 (16.7) | 20 (13.3%) | 6 (4.0%) | 0 | 0 |
| Hypertriglyceridemia | 18 (20.2) | 16 (18.0%) | 1 (1.1%) | 1 (1.1%) | 0 | 25 (16.7) | 23 (15.3%) | 1 (0.7%) | 1 (0.7%) | 0 |
| Weight decreased | 16 (18.0) | 9 (10.1%) | 7 (7.9%) | 0 | 0 | 28 (18.7) | 14 (9.3%) | 13 (8.7%) | 1 (0.7%) | 0 |
| Gamma-glutamyltransferase increased | 15 (16.9) | 13 (14.6%) | 1 (1.1%) | 1 (1.1%) | 0 | 28 (18.7) | 21 (14.0%) | 5 (3.3%) | 3 (2.0%) | 0 |
| White blood cell count decreased | 13 (14.6) | 11 (12.4%) | 7 (7.9%) | 2 (2.2%) | 0 | 27 (18.0) | 23 (15.3%) | 10 (6.7%) | 2 (1.3%) | 0 |
| Fatigue | 8 (9.0) | 6 (6.7%) | 3 (3.4%) | 1 (1.1%) | 0 | 24 (16.0) | 21 (14.0%) | 5 (3.3%) | 1 (0.7%) | 0 |

AST, aspartate aminotransferase; ALT, alanine aminotransferase; SAE, serious adverse events; TEAEs: treatment-emergent adverse events.

## Supplementary Table 4. Pharmacokinetic parameters of FS-1502 (PK Parameter Analysis Set)

| **Dose**  **(mg/kg)** | **Regimen** | **N** | **Cmax**  **(µg/mL)** | **AUCinf**  **(µg* day /mL)** | **AUClast**  **(µg* day /mL)** | **AUC0-21day**  **(µg* day /mL)** | **t1/2**  **(day)** | **CL**  **(mL/day/kg)** | **Vz**  **(mL/kg)** | **ARCmax** | **ARAUC** |
| --- | --- | --- | --- | --- | --- | --- | --- | --- | --- | --- | --- |
| **Cycle 1** |  |  |  |  |  |  |  |  |  |  |  |
| 0.1 | Q4W | 3 | 1.25 (30.82) | 1.21 (42.30) | 1.11 (43.97) | 1.21 (42.31) | 0.626 (8.74) | 82.3 (51.62) | 74.4 (42.47) | NA | NA |
| 0.4 | Q4W | 5 | 7.16 (9.50) | 15.3 (14.76) | 15.0 (13.86) | 15.2 (14.46) | 1.31 (76.09) | 26.2 (14.42) | 49.6 (59.14) | NA | NA |
| 0.6 | Q4W | 7 | 12.6 (94.76) | 31.1 (22.04) | 30.6 (22.66) | 31.1 (22.02) | 1.63 (22.26) | 19.3 (21.34) | 45.3 (27.65) | NA | NA |
| 0.8 | Q4W | 4 | 12.8 (28.88) | 41.0 (45.96) | 40.6 (46.45) | 40.9 (45.91) | 2.31 (17.98) | 19.5 (39.86) | 65.1 (57.14) | NA | NA |
| 1.0 | Q4W | 6 | 19.3 (27.93) | 77.0 (36.20) | 76.7 (36.20) | 76.6 (35.83) | 2.42 (26.18) | 13.0 (36.33) | 45.4 (17.37) | NA | NA |
|  | Q3W | 3 | 26.5 (16.60) | 97.7 (11.04) | 95.8 (8.18) | 95.8 (8.18) | 3.03 (46.29) | 10.2 (10.42) | 44.8 (34.44) | NA | NA |
|  | （Pooled） | 9 | 21.4 (27.05) | 83.4 (29.11) | 82.6 (28.69) | 82.6 (28.44) | 2.61 (35.82) | 12.0 (34.49) | 45.2 (22.10) | NA | NA |
| 1.3 | Q4W | 6 | 22.9 (8.17) | 89.1 (19.85) | 88.8 (19.84) | 88.6 (19.45) | 2.47 (25.76) | 14.6 (25.58) | 52.0 (11.89) | NA | NA |
|  | Q3W | 4 | 26.6 (12.69) | 101 (28.23) | 100 (27.93) | 100 (27.75) | 2.40 (19.54) | 12.9 (22.06) | 44.7 (27.59) | NA | NA |
|  | （Pooled） | 10 | 24.3 (12.80) | 93.7 (23.51) | 93.3 (23.34) | 93.2 (23.14) | 2.44 (22.51) | 13.9 (24.09) | 48.9 (18.64) | NA | NA |
| 1.7 | Q4W | 5 | 31.6 (12.19) | 149 (10.33) | 148 (10.29) | 147 (10.13) | 3.24 (10.55) | 11.4 (10.73) | 53.3 (12.63) | NA | NA |
|  | Q3W | 4 | 30.6 (19.58) | 106 (45.01) | 105 (44.97) | 105 (44.90) | 2.60 (24.37) | 16.1 (72.25) | 60.2 (60.22) | NA | NA |
|  | （Pooled） | 9 | 31.1 (14.75) | 128 (28.09) | 127 (28.15) | 126 (27.97) | 2.93 (18.82) | 13.3 (62.56) | 56.3 (44.47) | NA | NA |
| 2.3 | Q3W | 14 | 43.7 (10.66) | 177 (22.76) | 171 (24.13) | 174 (22.03) | 3.18 (21.70) | 13.0 (26.85) | 59.6 (15.94) | NA | NA |
| 3.0 | Q3W | 9 | 59.9 (13.62) | 325 (16.97) | 310 (17.36) | 307 (16.61) | 4.80 (8.09) | 9.24 (18.12) | 63.9 (17.87) | NA | NA |
| 3.5 | Q3W | 1 | 76.5 (-) | 429 (-) | 409 (-) | 412 (-) | 4.45 (-) | 8.17 (-) | 52.4 (-) | NA | NA |
| **Cycle 3** |  |  |  |  |  |  |  |  |  |  |  |
| 0.1 | Q4W | 1 | 1.37 (-) | 1.47 (-) | 1.25 (-) | NA | 0.761 (-) | 67.8 (-) | 74.4 (-) | 0.860 (-) | 0.824 (-) |
| 0.6 | Q4W | 6 | 11.2 (41.61) | 38.6 (51.02) | 38.4 (51.16) | NA | 1.61 (40.52) | 15.5 (37.21) | 36.2 (46.65) | 0.903 (47.99) | 1.27 (49.65) |
| 0.8 | Q4W | 2 | 13.2 (37.87) | 50.8 (42.02) | 50.3 (42.59) | NA | 2.31 (15.51) | 15.7 (42.02) | 52.5 (27.40) | 0.972 (12.57) | 0.976 (4.39) |
| 1.0 | Q4W | 1 | 22.3 (-) | 104 (-) | 103 (-) | NA | 2.98 (-) | 9.65 (-) | 41.5 (-) | 1.26 (-) | 1.18 (-) |
|  | Q3W | 3 | 21.6 (18.53) | 99.9 (14.46) | 99.2 (14.68) | NA | 2.79 (2.19) | 10.0 (15.44) | 40.3 (15.12) | 0.817 (2.37) | 1.04 (21.28) |
| 1.3 | Q4W | 3 | 24.1 (7.17) | 109 (5.39) | 109 (5.36) | NA | 3.23 (2.26) | 11.9 (5.25) | 55.5 (4.85) | 1.07 (1.16) | 1.26 (35.56) |
|  | Q3W | 2 | 28.3 (15.93) | 134 (22.63) | 132 (22.03) | NA | 2.66 (0.02) | 9.73 (22.63) | 37.4 (22.60) | 1.05 (4.81) | 1.15 (11.71) |
| 1.7 | Q4W | 4 | 29.7 (18.07) | 158 (12.80) | 157 (12.70) | NA | 3.59 (13.04) | 10.8 (13.50) | 55.9 (13.01) | 0.975 (8.78) | 1.02 (6.63) |
|  | Q3W | 3 | 31.0 (18.50) | 168 (36.27) | 163 (35.16) | NA | 4.04 (26.70) | 10.1 (45.18) | 58.9 (13.08) | 1.08 (2.31) | 1.71 (30.92) |
| 2.3 | Q3W | 11 | 47.9 (15.36) | 263 (22.62) | 211 (35.87) | NA | 4.20 (16.85) | 8.75 (27.11) | 53.1 (15.91) | 1.09 (13.46) | 1.38 (19.09) |
| 3.0 | Q3W | 8 | 61.0 (9.17) | 366 (24.66) | 332 (23.06) | NA | 5.41 (16.20) | 8.19 (24.85) | 63.9 (15.02) | 1.06 (8.30) | 1.14 (24.59) |
| 3.5 | Q3W | 1 | 39.4 (-) | 250 (-) | 234 (-) | NA | 6.25 (-) | 14.0 (-) | 126 (-) | 0.515 (-) | 0.558 (-) |

C_max_, maximum concentration, AUC, area under the serum concentration-time curve; CL, total body clearance; Vz, the volume in the terminal state; ARCmax, accumulation ratio for C_max_; ARAUC, accumulation ratio for AUC; Q3W, once every 3 weeks; Q4W, once every 4 weeks; NA, not applicable.

Data were expressed as mean (CV%).

ARCmax was ratio of Cmax of Cycle 3 to Cycle 1.

ARAUC was ratio of AUC0-t of Cycle 3 to Cycle 1.

AUClast was AUC0-21day (Q3W) or AUC0-27day (Q4W).

## Supplementary Table 5. Pharmacokinetic parameters of total antibody (PK Parameter Analysis Set)

| **Dose**  **(mg/kg)** | **Regimen** | **N** | **Cmax**  **(µg/mL)** | **AUCinf**  **(µg* day /mL)** | **AUClast**  **(µg* day /mL)** | **AUC0-21day**  **(µg* day /mL)** | **t1/2**  **(day)** | **CL**  **(mL/day/kg)** | **Vz**  **(mL/kg)** | **ARCmax** | **ARAUC** |
| --- | --- | --- | --- | --- | --- | --- | --- | --- | --- | --- | --- |
| **Cycle 1** |  |  |  |  |  |  |  |  |  |  |  |
| 0.1 | Q4W | 3 | 1.55 (47.20) | 1.46 (49.51) | 1.55 (47.20) | 0.674 (7.30) | 64.6 (64.18) | 62.9 (56.15) | 1.55 (47.20) | NA | NA |
| 0.4 | Q4W | 3 | 25.0 (4.75) | 24.6 (4.79) | 24.9 (4.04) | 2.45 (50.69) | 16.0 (4.63) | 56.5 (45.53) | 25.0 (4.75) | NA | NA |
| 0.6 | Q4W | 6 | 50.1 (20.62) | 49.4 (20.17) | 49.4 (19.53) | 2.43 (84.61) | 12.0 (22.10) | 42.0 (68.52) | 50.1 (20.62) | NA | NA |
| 0.8 | Q4W | 3 | 78.8 (29.53) | 78.5 (29.53) | 78.0 (29.19) | 3.09 (17.72) | 10.1 (26.06) | 45.2 (27.46) | 78.8 (29.53) | NA | NA |
| 1.0 | Q4W | 5 | 125 (44.39) | 124 (43.67) | 124 (43.44) | 3.00 (18.43) | 7.99 (41.34) | 34.6 (25.58) | 125 (44.39) | NA | NA |
|  | Q3W | 2 | 166 (6.67) | 165 (6.60) | 165 (6.59) | 2.74 (0.35) | 6.01 (6.67) | 23.8 (6.32) | 166 (6.67) | NA | NA |
|  | （Pooled） | 7 | 136 (35.74) | 135 (35.22) | 134 (35.08) | 2.93 (16.27) | 7.37 (40.36) | 31.1 (29.24) | 136 (35.74) | NA | NA |
| 1.3 | Q4W | 4 | 125 (21.29) | 124 (21.02) | 123 (20.33) | 3.23 (23.56) | 10.4 (23.87) | 48.6 (10.38) | 125 (21.29) | NA | NA |
|  | Q3W | 4 | 160 (28.13) | 158 (26.60) | 158 (26.33) | 2.90 (21.27) | 8.10 (22.83) | 34.0 (20.03) | 160 (28.13) | NA | NA |
|  | （Pooled） | 8 | 141 (27.78) | 140 (26.57) | 139 (26.44) | 3.06 (21.71) | 9.19 (25.62) | 40.6 (22.78) | 141 (27.78) | NA | NA |
| 1.7 | Q4W | 4 | 233 (25.92) | 230 (25.79) | 225 (25.58) | 3.94 (14.72) | 7.31 (32.83) | 41.6 (33.27) | 233 (25.92) | NA | NA |
|  | Q3W | 4 | 178 (45.65) | 174 (45.47) | 174 (45.47) | 3.49 (17.16) | 9.53 (66.14) | 47.9 (84.04) | 178 (45.65) | NA | NA |
|  | （Pooled） | 8 | 204 (34.34) | 200 (34.33) | 198 (34.05) | 3.71 (16.05) | 8.34 (57.46) | 44.6 (68.43) | 204 (34.34) | NA | NA |
| 2.3 | Q3W | 12 | 238 (28.07) | 226 (29.05) | 230 (26.86) | 3.66 (21.73) | 9.67 (42.94) | 51.1 (29.30) | 238 (28.07) | NA | NA |
| 3.0 | Q3W | 7 | 470 (10.88) | 424 (15.24) | 421 (11.68) | 5.87 (20.80) | 6.38 (10.25) | 54.1 (25.02) | 470 (10.88) | NA | NA |
| 3.5 | Q3W | 0 | - | - | - | - | - | - | - | NA | NA |
| **Cycle 3** |  |  |  |  |  |  |  |  |  |  |  |
| 0.1 | Q4W | 1 | 1.53 (-) | 2.03 (-) | 1.97 (-) | NA | 0.691 (-) | 49.3 (-) | 49.1 (-) | 1.13 (-) | 0.893 (-) |
| 0.6 | Q4W | 6 | 17.5 (37.08) | 67.8 (56.65) | 67.5 (56.64) | NA | 2.28 (36.71) | 8.85 (37.08) | 29.1 (55.82) | 1.02 (56.26) | 1.17 (69.96) |
| 0.8 | Q4W | 2 | 20.8 (18.75) | 86.1 (34.06) | 85.3 (33.99) | NA | 3.27 (26.91) | 9.29 (34.06) | 43.8 (7.49) | 1.13 (4.26) | 1.04 (3.81) |
| 1.0 | Q4W | 1 | 26.1 (-) | 143 (-) | 142 (-) | NA | 4.15 (-) | 6.99 (-) | 41.9 (-) | 1.08 (-) | 0.793 (-) |
|  | Q3W | 3 | 35.3 (13.30) | 199 (7.11) | 192 (5.07) | NA | 3.90 (12.88) | 5.03 (7.00) | 28.3 (6.96) | 0.782 (23.80) | 0.871 (38.75) |
| 1.3 | Q4W | 3 | 32.0 (8.89) | 171 (7.72) | 169 (7.52) | NA | 4.14 (24.00) | 7.59 (7.42) | 45.3 (23.34) | 0.968 (9.41) | 0.964 (59.23) |
|  | Q3W | 2 | 41.4 (12.49) | 291 (55.51) | 284 (54.14) | NA | 3.14 (14.68) | 4.47 (55.51) | 20.2 (42.57) | 1.14 (7.16) | 1.53 (29.88) |
| 1.7 | Q4W | 4 | 47.9 (13.36) | 272 (18.93) | 265 (18.69) | NA | 4.79 (12.81) | 6.26 (18.99) | 43.3 (18.29) | 0.981 (12.21) | 0.992 (20.58) |
|  | Q3W | 3 | 41.5 (14.73) | 293 (36.61) | 265 (32.80) | NA | 5.32 (22.66) | 5.81 (44.04) | 44.5 (17.88) | 0.867 (38.97) | 1.68 (30.20) |
| 2.3 | Q3W | 11 | 57.7 (21.59) | 381 (31.28) | 287 (42.64) | NA | 5.30 (23.11) | 6.04 (39.29) | 46.2 (37.49) | 1.02 (17.40) | 1.25 (27.04) |
| 3.0 | Q3W | 8 | 70.6 (18.97) | 533 (30.24) | 451 (29.82) | NA | 6.69 (20.60) | 5.63 (37.10) | 54.3 (32.87) | 1.01 (20.88) | 1.11 (32.47) |
| 3.5 | Q3W | 1 | 54.4 (-) | 424 (-) | 387 (-) | NA | 6.64 (-) | 8.26 (-) | 79.1 (-) | 0.573 (-) | 0.611 (-) |

C_max_, maximum concentration, AUC, area under the serum concentration-time curve; CL, total body clearance; ARCmax, accumulation ratio for C_max_; ARAUC, accumulation ratio forAUC; Q3W, once every 3 weeks; Q4W, once every 4 weeks; NA, not applicable; -, no available.

Data were expressed as mean (CV%).

ARCmax was ratio of Cmax of Cycle 3 to Cycle 1.

ARAUC was ratio of AUC0-t of Cycle 3 to Cycle 1.

AUClast was AUC0-21day (Q3W) or AUC0-27day (Q4W).

Individuals with serum concentration of total antibody >5% of Cmax were excluded from the descriptive statistics.

## Supplementary Table 6. Pharmacokinetic parameters of unconjugated MMAF (PK Parameter Analysis Set)

| **Dose**  **(mg/kg)** | **Regimen** | **N** | **Cmax**  **(ng/mL)** | **AUClast**  **(ng* day /mL)** | **Tmax**  **(day)** | **t1/2**  **(day)** | **ARCmax** | **ARAUC** |
| --- | --- | --- | --- | --- | --- | --- | --- | --- |
| **Cycle 1** |  |  |  |  |  |  |  |  |
| 0.1 | Q4W | 3 | 0.0394 (52.94) | 0.0943 (50.33) | 1.03 (1.02, 2.03) | 1.58 (49.98) | NA | NA |
| 0.4 | Q4W | 5 | 0.0688 (54.80) | 0.603 (41.99) | 2.04 (1.05, 3.05) | 7.21 (32.61) | NA | NA |
| 0.6 | Q4W | 7 | 0.0865 (33.50) | 0.886 (35.42) | 4.04 (1.04, 4.05) | 8.42 (23.47) | NA | NA |
| 0.8 | Q4W | 4 | 0.0681 (59.90) | 0.861 (54.49) | 3.05 (1.05, 7.03) | 7.98 (26.43) | NA | NA |
| 1.0 | Q4W | 6 | 0.210 (67.22) | 2.71 (61.97) | 3.55 (1.04, 5.05) | 8.05 (24.66) | NA | NA |
|  | Q3W | 3 | 0.118 (47.80) | 1.53 (28.12) | 1.05 (0.984, 8.03) | 7.54 (12.79) | NA | NA |
|  | （Pooled） | 9 | 0.173 (71.92) | 2.24 (66.98) | 2.07 (0.984, 8.03) | 7.92 (22.09) | NA | NA |
| 1.3 | Q4W | 6 | 0.217 (128.54) | 2.79 (77.15) | 3.04 (1.03, 7.04) | 7.87 (30.92) | NA | NA |
|  | Q3W | 4 | 0.251 (63.75) | 3.07 (56.69) | 3.05 (1.05, 7.03) | 5.82 (30.35) | NA | NA |
|  | （Pooled） | 10 | 0.230 (108.54) | 2.90 (66.12) | 3.04 (1.03, 7.04) | 6.97 (33.27) | NA | NA |
| 1.7 | Q4W | 5 | 0.357 (106.98) | 4.86 (80.47) | 5.03 (1.03, 9.06) | 7.45 (18.91) | NA | NA |
|  | Q3W | 4 | 0.570 (153.41) | 6.80 (129.15) | 5.04 (1.06, 5.06) | 5.37 (48.90) | NA | NA |
|  | （Pooled） | 9 | 0.439 (164.67) | 5.64 (126.56) | 5.03 (1.03, 9.06) | 6.44 (32.40) | NA | NA |
| 2.3 | Q3W | 14 | 0.533 (59.72) | 5.53 (46.48) | 5.04 (1.04, 7.05) | 7.65 (45.43) | NA | NA |
| 3.0 | Q3W | 9 | 0.628 (44.29) | 7.40 (39.97) | 7.03 (2.04, 21.0) | 9.77 (20.24) | NA | NA |
| 3.5 | Q3W | 1 | 0.334 (-) | 3.90 (-) | 7.04 (7.04, 7.04) | 18.9 (-) | NA | NA |
| **Cycle 3** |  |  |  |  |  |  |  |  |
| 0.1 | Q4W | 1 | 0.0250 (-) | 0.0755 (-) | 1.02 (1.02, 1.02) | 1.53 (-) | 1.12 (-) | - (-) |
| 0.6 | Q4W | 6 | 0.0870 (50.49) | 1.08 (51.95) | 1.54 (1.04, 7.05) | 9.84 (23.51) | 1.09 (33.32) | 1.31 (26.23) |
| 0.8 | Q4W | 2 | 0.0798 (26.47) | 1.23 (34.97) | 1.04 (1.04, 1.05) | 11.7 (27.94) | 1.08 (22.98) | 1.23 (15.15) |
| 1.0 | Q4W | 1 | 0.201 (-) | 3.27 (-) | 7.06 (7.06, 7.06) | 9.20 (-) | 1.10 (-) | 1.22 (-) |
|  | Q3W | 3 | 0.175 (38.57) | 2.18 (40.41) | 1.06 (1.03, 3.05) | 10.4 (17.41) | 1.49 (28.01) | 1.43 (25.11) |
| 1.3 | Q4W | 3 | 0.137 (41.84) | 2.34 (36.08) | 5.06 (5.03, 5.06) | 8.05 (10.72) | 0.540 (70.38) | 0.812 (46.34) |
|  | Q3W | 2 | 0.257 (29.56) | 4.05 (36.26) | 3.54 (2.05, 5.03) | 6.61 (29.90) | 0.920 (62.31) | 1.20 (48.59) |
| 1.7 | Q4W | 4 | 0.267 (10.41) | 4.63 (15.18) | 3.05 (1.03, 9.06) | 10.2 (23.58) | 1.06 (8.01) | 1.25 (10.21) |
|  | Q3W | 3 | 0.433 (81.99) | 5.97 (76.66) | 7.06 (5.03, 9.04) | 8.80 (47.78) | 0.724 (106.83) | 0.934 (76.83) |
| 2.3 | Q3W | 11 | 0.338 (40.72) | 3.89 (50.22) | 5.03 (1.06, 5.04) | 15.6 (77.87) | 0.687 (72.40) | 0.991 (57.97) |
| 3.0 | Q3W | 8 | 0.911 (53.56) | 11.8 (32.59) | 5.04 (2.03, 17.0) | 18.8 (80.13) | 1.36 (37.49) | 1.76 (30.65) |
| 3.5 | Q3W | 1 | 0.212 (-) | 4.06 (-) | 11.1 (11.1, 11.1) | 19.4 (-) | 0.634 (-) | 0.941 (-) |

C_max_, maximum concentration, AUC, area under the serum concentration-time curve; CL, total body clearance; ARCmax, accumulation ratio for C_max_; ARAUC, accumulation ratio forAUC; Q3W, once every 3 weeks; Q4W, once every 4 weeks; NA, not applicable; -, no available.

Data were expressed as mean (CV%).

ARCmax was ratio of Cmax of Cycle 3 to Cycle 1.

ARAUC was ratio of AUC0-t of Cycle 3 to Cycle 1.

AUClast was AUC0-21day (Q3W) or AUC0-27day (Q4W).

Supplementary Figure 1. Forest plot of subgroup analysis in HER2-positive breast cancer patients Data of objective response was presented as response rate (95% CI) for each subgroup. 95% CIs were obtained using Clopper–Pearson method. CI: confidence interval; ECOG: Eastern Cooperative Oncology Group.


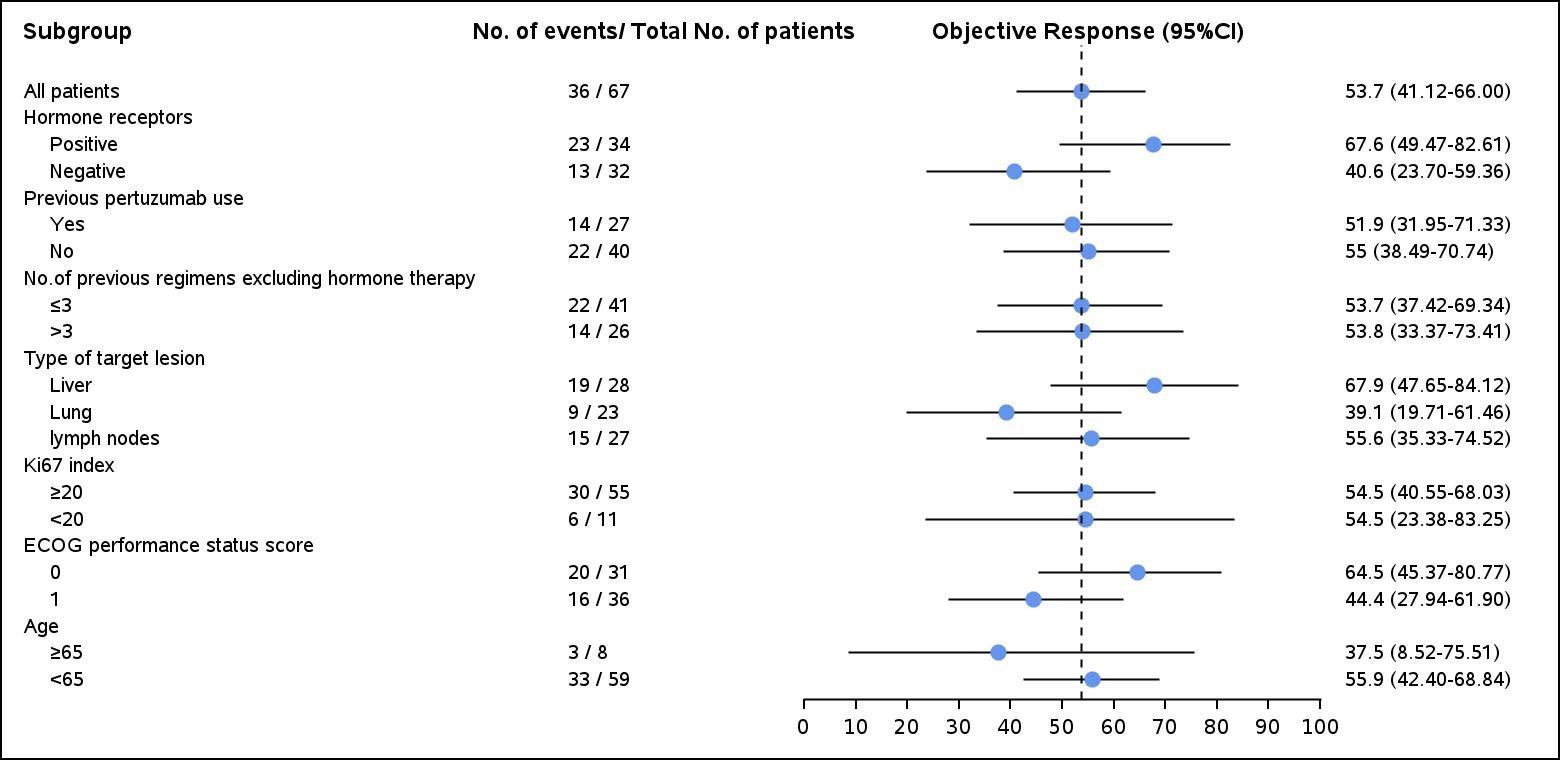


## Supplementary Figure 2. Serum concentration of FS-1502 at (A) Cycle 1 and (B) Cycle 3 (PK Concentration Analysis Set)

FS-1502 concentration results of 71 patients were included for Cycle 1, and results of 46 patients were included for Cycle 3. Data was presented as mean (standard deviation). Q3W: once every 3 weeks; Q4W: once every 4 weeks.

Cycle 1


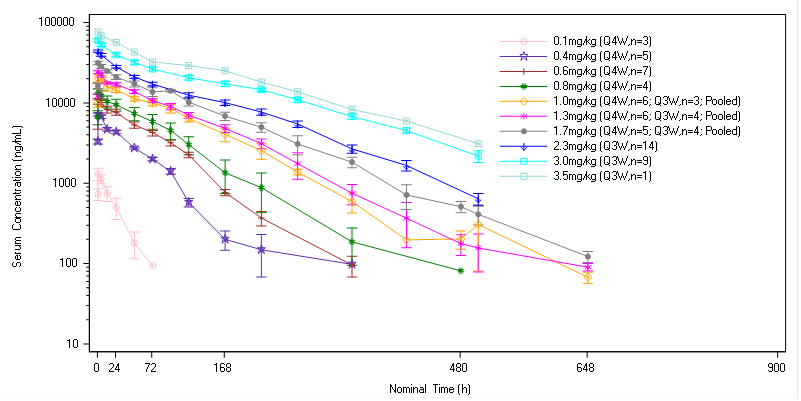


Cycle 3


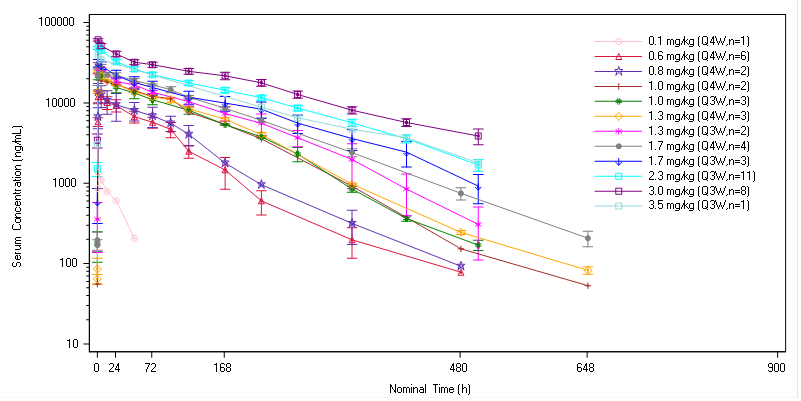


## Supplementary Figure 3. Serum concentration of total antibody at (A) Cycle 1 and (B) Cycle 3 (PK Concentration Analysis Set)

Total antibody concentration results of 71 patients were included for Cycle 1, and results of 46 patients were included for Cycle 3. Data was presented as mean (standard deviation). Q3W: once every 3 weeks; Q4W: once every 4 weeks.

Cycle 1:


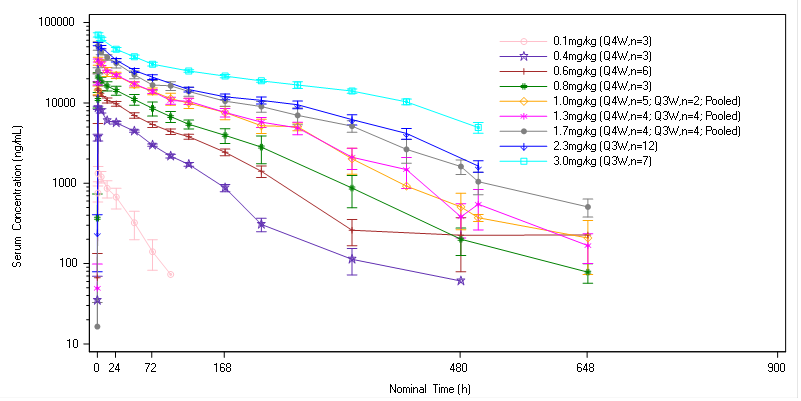


Cycle 3:


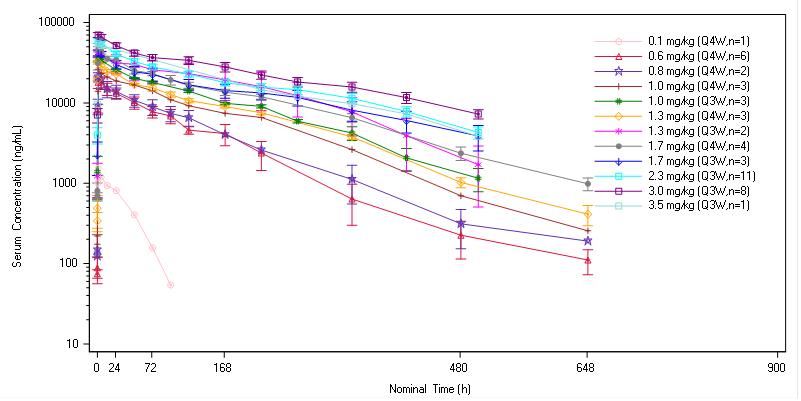


## Supplementary Figure 4. Serum concentration of unconjugated MMAF at (A) Cycle 1 and (B) Cycle 3 (PK Concentration Analysis Set)

Unconjugated MMAF concentration results of 71 patients were included for Cycle 1, and results of 46 patients were included for Cycle 3. Data was presented as mean (standard deviation). Q3W: once every 3 weeks; Q4W: once every 4 weeks.

Cycle 1:


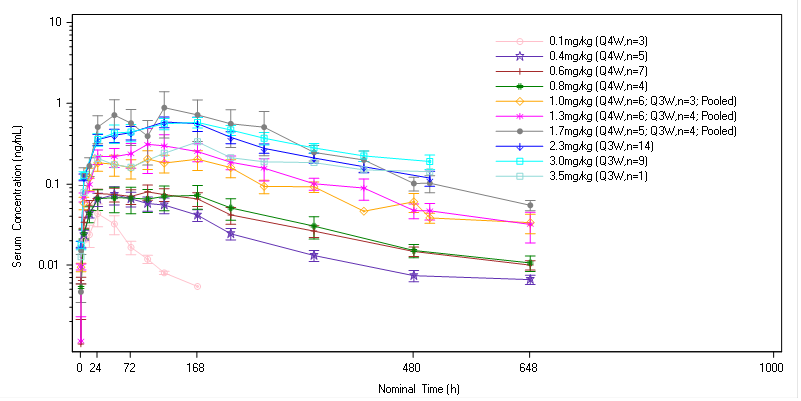


Cycle 3:


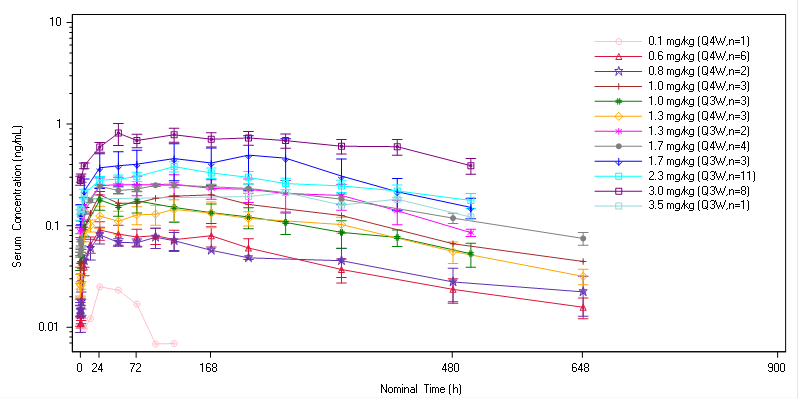


## Supplementary Note 5. Study protocol and amendments

| **Clinical Study Protocol** | |
| --- | --- |
| **Protocol Title:** | **A multicenter, open-label, single-arm phase Ia/Ib clinical study: a dose-escalation of FS-1502 in patients with HER2-expressing advanced malignant solid tumors and a dose-expansion in patients with HER2-positive locally advanced or metastatic breast cancer** |
| **Protocol No.** | FS-CY1502-Ph1-01 |
| **Investigational Product:** | FS-1502 |
|  |  |
| **Sponsor:** | Shanghai Fosun Pharmaceutical Industry Development Co., Ltd.  1289 Yishan Road, Xuhui District, Shanghai 200233, China |
| **Sponsor Contact:** | Wang Xuesong  Tel: 021 - 33987588 |
| **Principal Investigator:** | Xu Binghe  Tel: 010 - 87788826 |
| **Protocol version:** | Version 5.0 |
| **Version Date:** | January 27, 2022 |

**Confidentiality Statement**

All information in this protocol is confidential and is the property of the Sponsor.

**The Table of Contents**

[FS-1502Clinical Trial Plan 23](#_Toc2317)

[Investigator Signature Page 25](#_Toc12280)

[Protocol Summary 26](#_Toc924)

[1Research background 41](#_Toc16947)

[1.1medical background 41](#_Toc25051)

[1.2Introduction to Study Drug 41](#_Toc6348)

[1.3Preclinicalpharmacological studies 45](#_Toc19989)

[1.4PreclinicalToxicology studies 49](#_Toc20364)

[1.5Preclinicalpharmacokinetic study 53](#_Toc25218)

[1.6Theoretical basis of this clinical study 56](#_Toc32635)

[2Study Objectives and Endpoints 60](#_Toc29573)

[2.1Study Objectives 60](#_Toc28631)

[2.2Study Endpoints 62](#_Toc25024)

[2.3Definition of study endpoints: 62](#_Toc27707)

[3Research plan 64](#_Toc12900)

[3.1Overall Study Plan 64](#_Toc15885)

[3.2Study Design 65](#_Toc15637)

[3.3End of study 67](#_Toc78)

[4Selection of study subjects 68](#_Toc12090)

[4.1Inclusion criteria 68](#_Toc18942)

[4.2Exclusion criteria 69](#_Toc2313)

[4.3Discontinuation criteria 70](#_Toc19477)

[5Study Flow 72](#_Toc6715)

[5.1 Iaperiod 72](#_Toc12236)

[5.2 Ibperiod 75](#_Toc22332)

[6Study Drug and Study Method 79](#_Toc15)

[6.1Study Drug 79](#_Toc5352)

[6.2Packaging and Labeling of Study Drug 79](#_Toc98)

[6.3Storage and Transport of Study Drug 79](#_Toc27857)

[6.4Medication management 79](#_Toc21536)

[6.5Medication Dispensation 79](#_Toc11474)

[6.6Dosage and cycle administered 80](#_Toc31112)

[6.7With theMethod of medicine 80](#_Toc18159)

[6.8Concomitant medications/Treatment 80](#_Toc23634)

[6.9Permitted and prohibited medications and non-pharmacological treatments 81](#_Toc6869)

[6.10Drug overdose 81](#_Toc12207)

[6.11Treatment Compliance 81](#_Toc12190)

[6.12Principles for Handling Adverse Reactions during the Study 82](#_Toc22335)

[7Study Assessment 88](#_Toc23691)

[7.1Safety Assessment 88](#_Toc21410)

[7.2Efficacy evaluation 95](#_Toc23209)

[7.3Pharmacokinetic and Immunogenicity Assessment 96](#_Toc22681)

[8Data management 100](#_Toc891)

[8.1Data Entry 100](#_Toc5643)

[8.2Database Lock 100](#_Toc5346)

[9Statistical and Statistical Analysis 101](#_Toc14650)

[9.1Calculation of sample size 101](#_Toc23626)

[9.2Analysis set 101](#_Toc10942)

[9.3Safety Analysis 102](#_Toc29315)

[9.4Efficacy Analysis 102](#_Toc29261)

[9.5Pharmacokinetic and Immunogenicity Analysis 103](#_Toc17325)

[9.6Interim Analysis 103](#_Toc31171)

[10Study management 104](#_Toc12470)

[10.1Ethical Considerations 104](#_Toc28289)

[10.2Informed Consent 104](#_Toc14031)

[10.3Protocol Amendment 105](#_Toc26546)

[10.4Protocol Deviations 105](#_Toc4242)

[10.5Patient Confidentiality and Privacy 106](#_Toc18217)

[10.6Monitoring 106](#_Toc19517)

[10.7Quality Assurance and Quality Control 107](#_Toc14185)

[10.8Direct Reference 107](#_Toc7412)

[10.9Data Recording and Retention 107](#_Toc577)

[10.10Insurance and Patient Compensation 108](#_Toc31618)

[10.11Storage and use of biological specimens 108](#_Toc29057)

[10.12Study Interruption and Early Termination 108](#_Toc25903)

[10.13Study Summary Report 108](#_Toc22613)

[10.14Information Disclosure and Data Publication Policy 108](#_Toc26303)

[10.15Statement of Conflict of Interest 109](#_Toc19618)

[Attachments1 Pharmacokinetic and Immunogenic Blood Sample Collection Time and Volume 110](#_Toc19847)

[Attachments2Dose ramp-up phase assessment form 112](#_Toc16734)

[Attachments3Dose Expansion Phase Assessment Form 115](#_Toc6070)

[Attachments 4ECOGphysical condition 118](#_Toc32456)

[Attachments5New York College of Cardiology Cardiac Function Classification (NYHA) 119](#_Toc19503)

[Attachments6 May prolongQTcof drugs 120](#_Toc22865)

**FS-1502 Clinical Trial Plan**

FS-1502 is developed by Shanghai Fosun Pharmaceutical Industry Development Co., Ltd. FS-1502 is a recombinant HER2 humanized monoclonal antibody MMAF conjugate (freeze and lyophilized powder) intended for development for the indication of HER2-positive advanced malignant solid tumors. According to the Technical Guidelines for Clinical Trials of Antineoplastic Drugs, FS-1502 plans to conduct phase I clinical trial in human. (Human safety tolerability testing, human pharmacokinetics, and preliminary antitumor activity exploration), Phase II clinical trial (exploratory trial) and Phase III clinical trial (confirmatory trial). After obtaining the clinical trial approval document, the Company will entrust professional qualified institutions to draft the trial protocol for each phase in accordance with the clinical trial management practice (GCP) and relevant national technical guidelines. After the approval of the Hospital Ethics Committee, the Company will organize and implement the trial protocol in accordance with the relevant principles of GCP. Qualified data management teams and statistics teams complete data analysis.

**Sponsor Signature Page**

| Protocol Title: | A multicenter, open-label, single-arm phase Ia/Ib clinical study: a dose-escalation of FS-1502 in patients with HER2-expressing advanced malignant solid tumors and a dose-expansion in patients with HER2-positive locally advanced or metastatic breast cancer | |
| --- | --- | --- |
| Protocol No.: | FS-CY1502-Ph1-01 | |
| Version Date and Version Number: | January 27, 2022, Version 5.0 | |
|  |  | |
| Name of Sponsor: | Shanghai Fosun Pharmaceutical Industry Development Co., Ltd. | |
|  |  | |
|  |  | |
| Sponsor's address: | 1289 Yishan Road, Xuhui District, Shanghai, 200233 | |
|  |  | |
| Name of approver: |  | |
| Position: |  | |
|  |  | |
| Signature of the approver: ______________ | | Signature Date: _________________ |

**Investigator Signature Page**

| Protocol Title: | A multicenter, open-label, single-arm phase Ia/Ib clinical study: a dose-escalation of FS-1502 in patients with HER2-expressing advanced malignant solid tumors and a dose-expansion in patients with HER2-positive locally advanced or metastatic breast cancer |
| --- | --- |
| Protocol No.: | FS-CY1502-Ph1-01 |
| Version Date and Version Number: | January 27, 2022, Version 5.0 |

I have read the protocol and agree to conduct the clinical study in accordance with all the provisions of the protocol, the current regulations and the ethical principles of the Declaration of Helsinki.

Principal Investigator Name:

| Signature of Principal Investigator: ________________ | Date: ________________ |
| --- | --- |

**Protocol Synopsis**

| **Protocol No.:** | FS-CY1502-Ph1-01 |
| --- | --- |
| **Protocol Title:** | A multicenter, open-label, single-arm phase Ia/Ib clinical study: a dose-escalation of FS-1502 in patients with HER2-expressing advanced malignant solid tumors and a dose-expansion in patients with HER2-positive locally advanced or metastatic breast cancer |
| **Study Drug:** | FS-1502 |
| **Clinical Study Stage:** | Phase Ia/Ib |
| **Number of patients:** | Expected to enroll approximately 92 patients |
| **Clinical Research Site:** | Ia: 1 – 5 sites  Ib: 1 – 10 sites |
| **Study Period:** | **Stage Ia:**   - Screening period (Day -28 - Day -1); - FS-1502 single-agent continuous use: - Patients enrolled based on the 1.2 and 2.0 protocols: IV every 4 weeks, 28-day once a cycle; - Patients enrolled based on the Version 3.0 and Version 4.0 regimens: IV every 3 weeks starting at 1.0 mg/kg for 21-day once a cycle; - End of treatment follow-up (30 days after the last dose); - Survival follow-up. (Telephone follow-up every 3 months until the end of the study).   **Stage Ib:**   - Screening period (Day -28 - Day -1); - Treatment period: FS-1502 2.3mg/kg IV, 21 days once a cycle; - End of treatment follow-up (30 days after the last dose); - Survival follow-up. (Telephone follow-up every 3 months until the end of the study).   All patients will continue treatment until no clinical benefit, intolerable toxicity, death, investigator's decision, or patient's voluntary withdrawal from the study.  End of study was defined as 1 year after the start of treatment for the last enrolled patient or 50% death of patients, whichever was earlier.  At the end of the study, patients without disease progression will continue to use FS-1502 at the discretion of the investigator based on clinical benefit. |
| **Study Objectives:** | **Phase Ia Primary Objectives:**   - To observe the safety and tolerability of continuous intravenous infusion of FS-1502 single agent in patients with advanced HER2-expressing malignant solid tumors. - To determine the maximum tolerated dose (MTD), the recommended phase 2 dose (recommend phase 2 dose, RP2D) and dose-limiting toxicity (DLT) of FS-1502 monotherapy in patients with advanced HER2-expressing solid tumors.   **Phase Ia Secondary Objectives:**   - To evaluate the antitumor activity of FS-1502 single-agent administered continuously by intravenous infusion in patients with HER2-expressing advanced malignant solid tumors. - To observe the pharmacokinetic (PK) characteristics and immunogenicity of FS-1502 single agent administered continuously intravenously in patients with HER2-expressing advanced malignant solid tumors.   **Phase Ib Primary Objectives:**   - To evaluate the efficacy of continuous intravenous infusion of FS-1502 single agent in patients with HER2-positive locally advanced or metastatic breast cancer.   **Phase Ib Secondary Objectives:**   - To evaluate the safety and tolerability of continuous intravenous infusion of FS-1502 single agent in patients with HER2-positive locally advanced or metastatic breast cancer. - To evaluate the population pharmacokinetics (Pop PK) profile and immunogenicity of continuous intravenous infusion of FS-1502 single agent in patients with HER2-positive locally advanced or metastatic breast cancer. |
| **Study Endpoints:** | **Phase Ia Primary Endpoints:**   - Safety: occurrence of dose limiting toxicities (DLTs) in the first treatment cycle after the first dose of a single agent. - The maximum tolerated dose (MTD) of FS-1502 monotherapy and the phase 2 dose (RP2D) recommended.   **Phase Ia Secondary Endpoints:**   - Other safety endpoints: - Type and frequency of treatment-emergent adverse events (AEs), with toxicity grade evaluated according to the National Cancer Institute Common Toxicity Criteria for Adverse Events version (national cancer institute common terminology criteria for adverse events, NCICTCAE) 5.0 - Serious adverse events (SAEs) and AE leading to treatment discontinuation. - Frequency and cause of death within 30 days after the last dose. - The most severe laboratory safety results according to NCICTCAE version 5.0. - Changes in vital signs and ECOG scores. - Other efficacy endpoints: progression free survival (PFS), tumor response rate (ORR), overall survival (OS), 1-year OS rate, duration of response (DOR) and clinical benefit rate (CBR). - PK parameters for FS-1502, total antibody, and MMAF. - FS-1502 Anti-Drug Antibody (ADA) and Neutralizing Antibody (NAb).   **Phase Ib Primary Endpoint:**   - ORR   **Phase Ib Secondary Endpoints:**   - Other efficacy endpoints: PFS, DOR, CBR, 1-year OS rate and OS; - Type and frequency of AEs, grade evaluated according to NCI CTCAE version 5.0; SAEs occurring during the study and AE leading to permanent discontinuation; - Frequency and cause of death within 30 days of last dose; - Laboratory safety test results graded according to NCI CTCAE version 5.0. - Population Pop PK parameters for FS-1502, Total Antibodies, and MMAF. - FS-1502 Anti-Drug Antibody (ADA) and Neutralizing Antibody (NAb). |
| **Study Design:** | **Phase Ia**  According to the main dosage level designed, different volumes of FS-1502 were drawn, added into 100 ml of 0.9% normal saline, and intravenous drip was completed within 60-90 minutes. The frequency and treatment period of FS-1502 were as follows:   - Patients enrolled based on the protocol version 1.2-2.0: IV every 4 weeks for 28-day once a cycle; - Based on the Version 3.0 and Version 4.0 regimens: dosing by intravenous drip every 3 weeks starting at 1.0 mg/kg for 21-day once a cycle;   **Note: The dosing frequency will be adjusted while the dose escalation continues, and new patients will be given every 3 weeks for a 21-day cycle starting from the effective date of the version 3.0 protocol; The safety, efficacy, and PK data for the 3-week dosing regimen will be fully evaluated subsequently, if necessary, the dosing frequency would adjustto once every 2 weeks.**  **Dose escalation principle:**   - According to the available non-clinical data, 0.1 mg/kg is tentatively used as the starting dose, and the dose is titrated by 3 + 3. Seven dose groups are preset in this study (as shown in the following table). If 1.3 mg/kg is safe and well tolerated and meets the linear kinetic characteristics, dose escalation will continue with a subsequent 33% escalation (proportional dose escalation may be adjusted based on previous data) up to the MTD or RP2D, based on analysis of PK/PD, Safety and efficacy results.,. A total of 21 to 42 evaluable patients are expected to be enrolled. However, the doses for the escalation phase were not limited to these dose groups and the number of patients was not limited to 21-42. - If 1 of 3 patients in a dose group had a DLT, additional 3 patients were added to that dose group. If none of the newly added patients experienced a DLT (1/6), escalate to the next dose. If 2 or more patients have DLTs, enrollment in that dose group should be stopped and escalation to the next dose group should not be allowed; - DLT events were observed in <33% of patients evaluable for DLT events (Up to 1 out of 6 patients or 0 out of 3 patients). The highest dose group was considered as the MTD; If no MTD was observed, the appropriate treatment exposure dose or RP2D was determined based on the antitumor efficacy evaluation, safety data, and PK data. - If the MTD is reached at the starting dose, a lower dose will be considered. - During the DLT observation period, if the patient was given less than 80% of the planned dose due to non-DLT reasons, case replacement was required. - After each DLT observation for a dose group is completed, the investigator and the sponsor will review the safety data for the dose group, efficacy data and PK data that have occurred in all previously enrolled patients. - When more than 3 eligible patients were screened in a dose group, all patients were allowed and patients with expanded enrollment were not included in the DLT assessment.   **Main Dose Design:**   \| Dosage \| 0.1  mg/kg \| 0.2  mg/kg \| 0.4  mg/kg \| 0.6  mg/kg \| 0.8  mg/kg \| 1.0  mg/kg \| 1.3  mg/kg \| \| --- \| --- \| --- \| --- \| --- \| --- \| --- \| --- \| \| Increment \|  \| 100% \| 100% \| 50% \| 33% \| 25% \| 30% \| \| Number of cases \| 3 - 6 \| 3 - 6 \| 3 - 6 \| 3 - 6 \| 3 - 6 \| 3 - 6 \| 3 - 6 \|   **Stage Ib:**  An SMC meeting was held in January 2022, and after a comprehensive assessment of safety, PK data and efficacy, 2.3 mg/kg every 3 weeks was determined as RP2D to enter the Phase Ib clinical study. Phase Ib will enroll approximately 50 HER2-positve advanced breast cancer patients who have received at least 2 prior lines of treatment and have failed anti-HER2 therapy.  **Dose setting basis:**  The ICH S9 guidance recommends that the starting dose could be calculated on the basis of 1/6 of the highest non-severe toxic dose (HNSTD) in non-rodents or 1/10 of the STD10 (10% of animals exhibit a serious toxic effect) in rodents. Based on the results of the FS-1502 nonclinical repeat-dose toxicology study, the monkey HNSTD was 2.5 mg/kg, and a safety index of 1/10 was used according to the FDA Guidance: Estimating the Maximum Safe Starting Dose in Initial Clinical Trials for Therapeutics in Adult Healthy Volunteers 2005 " The human starting dose is 0.085 mg/kg. According to the principle of dose escalation and considering the clinical practice, the starting dose is accurate to one decimal place. The dose escalation regimen is set as follows: 0.1 mg/kg, 0.2 mg/kg, 0.4 mg/kg, 0.6 mg/kg, 0.8 mg/kg, 1.0 mg/kg, 1.3 mg/kg. Based on the PK/PD, and safety data, the MTD and RP2Dwill be determined by agreement between investigators and sponsor.  According to the lowest effectivedose in HCC1954 tumor-bearing mice and JIMT-1 tumor-bearing mice was 0.5 mg/kg and the highest non-severe toxic dose in cynomolgus monkeys was 2.5 mg/kg, the safety window for FS-1502 is approximately 6-fold based on the animal plasma AUC calculation method (AUC_HNSTD_/AUC_MED_), indicating adequate safety window for FS-1502 in clinical studies.  **Dose modification:**  Dose adjustments were not allowed during the DLT observation period. Dose interruptions due to non-DLT-related toxicities were allowed during the first cycle.  In dose escalation stage, dose interruption was allowed after DLT observation period until the toxicity resolved to Grade 0-1. Dose reduction was allowed a maximum of 3 times, onse dose grade once time. The lowest dose in Phase Ia was 0.2 mg/kg, and the lowest dose in Phase Ib was 1.0 mg/kg.  After determination of RP2D, investigators may adjust the treatment to RP2D for Phase Ia patients who are still on treatment based on patients’ clinical benefit and patients' consent.  **DLT Assessment:**  To evaluate the incidence of DLTs within the first cycle (21 or 28 days) according to NCI-CTCAE (Version 5.0).  DLTs are defined as dose-limiting toxicity that occur during the DLT observation period, which are AEs or laboratory abnormalities judged by the Investigator and/or the Sponsor to be at least possibly related to FS-1502 and according to NCI-CTCAE V5.0 that meet any of the following criteria.  **NCI-CTCAE 5.0 Grade 4 or higher hematological toxicity:**   - Grade 4 neutropenia, lasting for ≥ 3 days after symptomatic treatment; - Grade 4 febrile neutropenia (fever≥ 38.0℃ for 1 hour or ≥ 38.3℃); - Grade 4 thrombocytopenia or Grade 3 thrombocytopenia with significant bleeding;   **NCI-CTCAE 5.0 Grade 3 or higher non-hematologic toxicities:**   - Any Grade 3 or higher elevated ALT, AST, total bilirubin; - QTc interval ≥ 501 ms (average of at least 2 ECG measurements) or QTc interval prolongation from baseline ≥ 60 ms; - Grade 3 nausea, vomiting, diarrhoea, and electrolyte disturbances (with the best supportive treatment lasting for 3 days); - Grade 2 or higher interstitial pneumonia; - Grade 3 and higher nephrotoxicity that did not recover within 7 days. (including nephrogenic hematuria, proteinuria, decreased creatinine clearance) - Grade 3 or higher amylase elevation or Grade 2 or higher pancreatitis - Toxicity events that, in the opinion of the investigator, require a dose adjustment or treatment suspension, or clinically unacceptable.   After each DLT observation for a dose group is completed, the investigator and the sponsor will review all safety, efficacy and PK data occurred in all enrolled patients.  **Tumor Assessment:**  Tumor assessments will be performed every 2 cycles (42/56 days ±7 days) according to RECIST v1.1, regardless of dose interruption or discontinuation, until disease progression, death, investigator decision, or patient withdrawal from the study.  **End of study:**  1 year after the start of treatment for the last enrolled patient or 50% death of patients, whichever was earlier. |
| **Subjects:** | Phase Ia: patient with HER2 expressing advanced malignant solid tumor;  Phase Ib: HER2 positive breast cancer patients who are relapsed after anti-HER2 therapy and at least 2 prior lines of prior treatment. |
| **Inclusion criteria:** | All of the following conditions must be met for patient enrollment:   1. ≥18 years, male or female; 2. **Phase Ia Dose Esacalation Stage:** Patients with HER2 Expressing Advanced Malignant Solid Tumors who have failed to Prior Standard of Care (including surgery, chemotherapy, radiotherapy, or biological therapy) or are unable to receive standard treatment or no standard treatment;   a HER2 overexpression: IHC3+, IHC2+/FISH+, or FISH+  b HER2 low expression: IHC1+, IHC2+/FISH-  **Phase Ib Dose Expansion Stage:** Histologically or cytologically confirmed HER2-positive advanced breast cancer patients who had received at least 2 prior lines of treatment and had failed anti-HER2 therapy, detail as follows:  a HER2 overexpression: IHC3+, IHC2+/FISH+;  b Patients with advanced breast cancer who have failed prior anti-HER2  therapy and have received at least 2 lines of therapy. If disease  progression happened during or within 12 months after the end of  adjuvant or neoadjuvant treatment, adjuvant or neoadjuvant treatment  could be considered as one therapy line;  c Evidence of disease progression or intolerable toxicity confirmed by the investigator or documented in medical history prior to enrollment;  d Patients can provide a written HER2 test report from local laboratory, and patients without HER2 test report must provide sufficient paraffin sections or fresh tumor tissue specimens to the study center or central laboratory for confirmation.   1. ECOG 0 or 1; 2. Expected survival of at least 12 weeks; 3. Patients with adequate organ and bone marrow function: absolute neutrophils   ≥1.0×109/L; Hb ≥ 90 g/L (no red blood cell transfusion within 14 days);  Platelet ≥100×109/L; Serum total bilirubin ≤1.5 x ULN,and ≤3.0 x ULN in  patients with Gilbert's syndrome. Aspartate aminotransferase (AST), alanine  aminotransferase (ALT) ≤ 2.5×ULN; In patients with liver metastases, AST  and ALT should be ≤ 5×ULN. Creatinine < 1.5 x ULN and creatinine clearance  ≥ 60 mL/min (calculated by the Cockroft-Gault equation); Blood potassium  ≥3.5 mmol/L; albumin ≥ 3 g/dL; Known left ventricular ejection fraction  (LVEF) >50%; Urinary protein ≤ 1 + or 24-hour urinary protein quantitation <  1.0 g;   1. At least one non-cranial measurable lesion assessed by RECIST 1.1; 2. Male or female patients of childbearing potential must agree to use effective methods of contraception, such as double barrier methods, condoms, oral or injectable contraceptives, intrauterine devices, etc., during the study and within 30 days of the last dose of study medication; 3. Be able to understand and voluntarily sign a written informed consent form. |
| **Exclusion criteria:** | Patients who meet any of the following conditions will not be enrolled:   1. Chemotherapy, targeted therapy, radiotherapy, etc. received 14 days or 5 half-lives (whichever is shorter) prior to the start of administration; Patients who have received major surgery, tumor immunotherapy, and monoclonal antibody therapy within 4 weeks before starting dosing; 2. Patients who have participated in other clinical trials within 4 weeks or 5 drug half-lives, whichever is shorter, prior to the start of dosing; 3. Patients previously treated with other anti-HER2 ADCs other than T-DM1; 4. Unstable CNS metastases lesions, patients with the following conditions were permitted    1. Having received local treatment and disease stable for more than 2 months;    2. No glucocorticoid or other dehydration therapy is required and the dose of antiepileptic drugs is stable (if applicable);    3. No meningeal metastases; 5. Patients with uncontrolled and stable diabetes. (Patients on stable insulin regimens or antidiabetic regimens who are assessed by the specialist as being well controlled were allowed to enroll); 6. The toxicities of prior anti-cancer therapy have not recovered (> NCI-CITCAE 5.0 Grade 2); Neurotoxicity in patients previously treated with chemotherapy requiring recovery to NCI-CTCAE 5.0 Grade 2 or less; 7. with corneal epitheliopathy (except mild punctate keratopathy); 8. Patients take drugs that prolong the QTc interval. (primarily Class Ia, Ic, III antiarrhythmic drugs) or the presence of risk factors for prolonged QTc interval, such as uncorrectable hypokalaemia, hereditary long QT syndrome; Medications with the potential to prolong the QTc interval are listed in Attachment 6. 9. Cardiac function and disease are one of the following: 10. QTc > 470 ms, the average of three times 12-lead electrocardiogram (ECG) measurements calculated by the instrument's QTc formula which were performed at the site during the screening period; 11. New York Heart Association (NYHA) class ≥ 3 congestive heart failure; 12. Clinically significant arrhythmias, including but not limited to complete left bundle branch conduction abnormality, Grade II atrioventricular block. 13. Pregnant or lactating women; 14. Known hypersensitivity to any of the excipients of FS-1502; 15. Clinically significant active bacterial, fungal, or viral infection, including hepatitis B (HBV surface antigen positive and HBV DNA over 1000 IU/ml) or Hepatitis C (HCV RNA positive), Human Immunodeficiency Virus Infection (HIV positive); 16. Any other disease or condition that, in the opinion of the Investigator, may affect compliance with the protocol or clinically significant for the patient to sign the ICF. (e.g., active or uncontrollable infection, etc.). |
| **Study Drug and Dosage:** | FS1502 dosage form and specification: FS-1502 frozen solution 60mg/12ml; FS-1502 lyophilized powder 30 mg/bottle.  **Phase Ia**  **Main dose:** 0.1 mg/kg, 0.2 mg/kg, 0.4 mg/kg, 0.6 mg/kg, 0.8 mg/kg, 1.0 mg/kg, 1.3 mg/kg. Dosage adjustment is allowed according to test data.  **Phase Ib:** 2.3 mg/kg  **Dosing method:** Dose was calculated according to the patient's body weight as specified in the protocol;  Different volumes of FS-1502 were drawn and added to 100 ml of 0.9% normal saline for intravenous drip in 60-90 minutes. If infusion-related reactions (IRRs) occur, slow the infusion rate or suspend the IV drip rate or decrease the IV drip rate for no more than 30 minutes, and antihistamine or corticosteroid therapy as clinically indicated. |
| **Safety Evaluation:** | These included monitoring and recording of AEs and SAEs, performing protocol-defined laboratory tests (including routine blood, chemistry, and urine tests), 12-lead ECG, and vital signs. The extent of toxic reactions will be evaluated according to the NCICTCAE Version 5.0 criteria. |
| **Efficacy evaluation:** | Tumor assessments were to be performed every 2 cycles (42/56 days ±7 days) per RECIST 1.1 until disease progression, death, investigator decision, or patient's voluntary withdrawal from the study. The final study analysis will be conducted at the end of treatment. |
| **Pharmacokinetic Evaluation:** | To investigate the PK profile of FS-1502 in humans, serum concentrations of FS-1502, total antibody, and MMAF will be measured during the dose escalation The blood samples were collected at the following time points:  **Phase Ia**  **Patients with 28-day once a cycle:**  Phase Ia 1st treatment cycle:   - D1: within 60 minutes before administration; 45 min (± 2 min) after the start of IV infusion, immediately after the end of administration (+ 2 min), 4 h (± 3 min), 12 h (± 3 min) after the end of administration; - D2:24 hours after the end of administration (±0.5 hours); - D3:48 hours after the end of administration (±0.5 hours); - D4:72 hours after the end of administration (±0.5 hours); - D5:96 hours after the end of administration (±0.5 hours); - D6:120 hours after the end of administration (±0.5 hours); - D8:168 hoursafter the end of administration (±0.5 hours); - D10:216 hours after the end of administration (±0.5 hours); - D15:336 hours after the end of administration (±0.5 hours); - D21:480 hoursafter the end of administration (±0.5 hours); - D28:648 hoursafter the end of administration (±0.5 hours);   Phase Ia 3rd treatment cycle:   - D1: within 60 minutes before administration; 45 min (± 2 min) after the start of IV infusion, immediately after the end of administration (+ 2 min), 4 h (± 3 min), 12 h (± 3 min) after the end of administration; - D2:24 h ±0.5 h after the end of administration; - D3:48 h ±0.5 h after the end of administration; - D4:72 h ±0.5 h after the end of administration; - D5:96 h ±0.5 h after the end of administration; - D6:120 h ±0.5 h after the end of administration; - D8:168 h ±0.5 h after the end of administration; - D10:216 h ±0.5 h after the end of administration; - D15:336 hours after the end of administration (±0.5 hours); - D21:480 hoursafter the end of administration (±0.5 hours); - D28:648 hoursafter the end of administration (±0.5 hours);   Phase Ia Cycle 4 and beyond:   - D1: within 60 minutes prior to administration.   **Patients in the 21-day once a cycle:**  Phase Ia 1st treatment cycle:   - D1: within 60 minutes before administration; Immediately after the end of dosing (+ 2 minutes), 4 hours after the end of dosing (± 3 minutes); - D2:24 hours after the end of administration (±0.5 hours); - D3:48 hours after the end of administration (±0.5 hours); - D4:72 hoursafter the end of administration (±0.5 hours); - D6:120 hours after the end of administration (±0.5 hours); - D8:168 hoursafter the end of administration (±0.5 hours); - D10:216 hours after the end of administration (±0.5 hours); - D12:264 hours after the end of administration (±0.5 hours); - D15:336 hoursafter the end of administration (±0.5 hours); - D18:408 hours after the end of administration (±0.5 hours);   Phase Ia 2nd treatment cycle:   - D1: within 60 minutes before administration;   Phase Ia 3rd treatment cycle:   - D1: within 60 minutes before administration; Immediately after the end of administration (+ 2 minutes), 4 hours after the end of administration (± 3 minutes); - D2:24 h ±0.5 h after the end of administration; - D3:48 h ±0.5 h after the end of administration; - D4:72 h±0.5 h after the end of administration; - D6:120 h±0.5 h after the end of administration; - D8:168 h ±0.5 h after the end of dosing; - D10:216 hours ±0.5 hours after the end of administration; - D12:264 hours after the end of administration (±0.5 hours); - D15:336 hours (± 0.5 hours) after the end of administration; - D18:408 hours after the end of administration (±0.5 hours);   Phase Ia Cycle 4 and beyond:   - D1: within 60 minutes prior to administration.   Phase Ib  Blood will be collected for Pop PK analysis within 60 minutes prior to D1 dosing in each treatment cycle. |
| **Immunogenicity Evaluation** | Immunogenicity blood collection: serum samples were collected for anti-drug antibodies (ADA) prior to D1 dosing in each treatment cycle until the last dosing. ADA-positive samples were further tested for neutralizing antibodies (NAb). |
| **Statistical Analysis:** | |
| **Sample size:** | The number of effective cases required for stage Ia is estimated to be about 21-42 cases.  The expected number of extended cases in Phase Ib is approximately 50, and the expected observed ORR is 50%, with a 95% confidence interval (35.5-64.5%). |
| **Analysis population:** | **Phase Ia**   - DLT Analysis Set: Includes patients in the ramp phase who experienced a DLT during the DLT observation period and patients who took at least 80% of the planned medication and completed the DLT observation period without a DLT. - Safety Analysis Set: Any patient with at least one use of FS-1502. - Efficacy Analysis Set: Patients with at least one FS-1502 use, baseline tumor assessment data and at least one post-baseline tumor assessment data. - PK Concentration Analysis Set: Patients who receivedFS-1502 treatment at least once and had at least 1 PK blood sample collected as scheduled with study drug concentration data. - PK Parameter Analysis Set: Patients who meet the protocol and received FS-1502 treatment and had at least 1 PK parameter during the trial. The patients who have significant protocol violations that affected the results of PK parameters or could not be estimated were not included in the PK parameter analysis set. - Immunogenicity Analysis Set: Patients with at least once FS-1502 treatment and at least one scheduled blood sample with anti-drug antibody test results.   **Phase Ib**   - Efficacy Analysis Set: Patients with at least one dose of FS-1502, with at least one postbaseline tumor assessment. - Safety Analysis Set: Any patient with at least one use of FS-1502. - PK Concentration Analysis Set: Patients who received FS-1502 treatment at least once and had at least 1 PK blood sample collected as scheduled and with study drug concentration data available. - PK Parameter Analysis Set: Patients who meet the protocol and received FS-1502 treatment and had at least 1 PK parameter during the trial. The patients who have significant protocol violations that affected the results of PK parameters or could not be estimated were not included in the PK parameter analysis set. - Immunogenicity Analysis Set: Patients with at least once FS-1502 treatment and at least one scheduled blood sample with anti-drug antibody test results. |
| **Safety Analysis:** | Safety was evaluated by a summary of DLTs, AEs, changes in clinical laboratory measurementsand vital sign measurements.  The incidence of DLTs will be assessed to determine the MTD. Adverse events will be summarized separately by the first cycle of the consecutive dosing phase and by the entire treatment period, and will be counted for drug-related AEs, SAEs, AEs with toxicity grade 3 or greater, and AEs leading to discontinuation.  Changes in clinical laboratory test results will be summarized according to the NCI-CTCAE (version 5.0) standard grading. For laboratory indicators, the maximum toxicity occurring during the study will be summarized in the form of counts and percentages. Descriptive statistics will be provided for changes in vital signs and ECOG scores compared to baseline levels. |
| **Efficacy Analysis:** | **Phase Ia**  Confirmed ORR will be calculated and ClopperPearson confidence intervals of 90% will be calculated in the Efficacy and Safety Analysis Sets. Survival analysis will be performed using the Kaplan-Meier method for PFS, DOR, and OS, and descriptive analysis will be performed for 1-year OS rate and DCR based on the investigator's assessment.  **Stage Ib**  Confirmed ORR will be calculated and a 95% ClopperPearson confidence interval will be calculated for the efficacy and safety analysis sets, respectively.  PFS and DOR were analyzed for survival, and 1-year OS rate and DCR were analyzed descriptively using the Kaplan-Meier method. |
| **Interim Analysis** | **Phase Ia**  This study plans to conduct an interim analysis after the end of the dose **esacalation** to assess the safety, metabolism, and preliminary antitumor activity of the drug.  **Stage Ib**  An interim analysis using Bayesian posterior probability will be conducted when approximately 20 patients complete 2 tumor assessments. If the predicted Pr (ORR < 20%) is >80%, i.e. fewer than 6 responses are observed in 20 evaluable patients, then an 80% probability of drug ORR being lower than standard of care is considered and early discontinuation of the cohort may be considered, otherwise enrollment will continue to approximately 50 patients. Bounds will be adjusted based on the actual number of patients in the Efficacy Analysis Set. The interim analysis margins are non-binding and the sponsor will consider the safety and efficacy data in the final decision. |
| **Pharmacokinetic and Immunogenicity Analysis:** | **PK Analysis:**  Using individual concentration-time data for FS-1502, total antibody, and MMAF, the following parameters were calculated: AUC0-t, AUC0-_-∞_, Cmax, Tmax, t1/2, Vd (or Vd/F), CL (or CL/F) and accumulation ratio (RAUC, RCmax), etc. Descriptive statistics were also provided for plasma concentration and PK parameters between dose groups.  **Pop PK Analysis:**  Concentration data obtained in this study or obtained in previous studies for the Pop PK analysis will be included in the Pop PK analysis and will be presented in a separate analysis report.  **Immunogenicity Analysis:**  The positive rates of ADA and neutralizing antibody (NAb) were summarized. |
| **Protocol Date:** | January 27, 2022 |

**Abbreviations**

| **Abbreviations** | **Full Name** |
| --- | --- |
| AE | Adverse Event |
| AIDS | acquired immunodeficiency syndrome |
| ALT | alanine aminotransferase |
| AST | glutamate aminotransferase |
| AUC | area under the concentration-time curve |
| AUC0-last /AUC0-t | area under the concentration-time curve from time 0 (predose) to the last time the concentration was measurable |
| AUC0-∞ | Area under the concentration-time curve from time 0 (predose) extrapolation to time infinity |
| DOR | Duration of Remission |
| Cmax | peak plasma concentration |
| CL/F | Drug clearance |
| CR | Complete remission |
| CrCL | creatinine clearance |
| CT | computed tomography |
| CYP | cytochrome P450 enzyme |
| DCR | disease control rate |
| DLT | Dose Limiting Toxicity |
| EC | Ethics Committee |
| ECG | Electrocardiogram |
| ECOG | Eastern Cooperative Oncology Group |
| eCRF | Electronic Case Report Form |
| FDA | U.S. Food and Drug Administration |
| GCP | Good Clinical Practice |
| HBV | Hepatitis B virus |
| HCV | Hepatitis C virus |
| HIV | human immunodeficiency virus |
| HNSTD | Maximum No Serious Side Effect Dose |
| IC50 | 50% maximum inhibitory concentration |
| ICF | Informed Consent Form |
| IEC | Independent Ethics Committee |
| INR | international normalized ratio |
| IRB | Institutional Review Board |
| LVEF | Left Ventricular Ejection Fraction |
| MAD | Maximum dose administered |
| MED | minimum effective dose |
| PET-CT | positron emission computed tomography |
| MTD | maximum tolerated dose |
| NCI-CTCAE | National Cancer Research Common Toxicity Criteria for Adverse Events |
| NOAEL | nontoxic response dose |
| NMPA | State Drug Administration |
| NYHA | New York Society of Cardiology |
| ORR | Objective response rate |
| OS | Overall Survival |
| PFS | Progression Free Survival |
| PK | pharmacokinetics |
| PLT | platelet |
| Pop PK | Population pharmacokinetics |
| PR | Partial Remission |
| RAS | murine sarcoma oncogene |
| RAUC | Accumulation ratio of area under the drug-hour curve |
| RCmax | Accumulation ratio of plasma peak concentration |
| RECIST | Response Evaluation Criteria in Solid Tumors |
| RP2D | Recommended Dosage in Phase II Clinical Studies |
| SAE | Serious Adverse Event |
| SD | Stable disease |
| Tmax | Time to peak plasma concentration |
| t1/2 | Half-life |
| TTP | Time to disease progression |
| ULN | upper limit of normal |
| Vd | apparent volume of distribution |

**1 Research Background**

**1.1 Medical background**

The human epidermal growth factor receptor (EGFR) family plays an important role in regulating the growth, differentiation and metastasis of epidermal cells. There are four members in the family: HER1 (EGFR), HER2, HER3 and HER4. Members of the family interact to generate heterologous or homodimers, which activate multiple signal transduction pathways in cells. The epidermal growth factor receptor 2 (HER2, c-erbB-2, HER2/neu) is a transmembrane protein with tyrosine kinase activity, and is composed of extracellular domain (ECD), transmembrane domain and intracellular domain (ICD), The extracellular domain consists of two ligand-binding domains and two cysteine-rich domains. The intracellular domain consists of a near membrane domain, a tyrosine kinase domain and a carboxyl terminal with rich tyrosine residues. HER2 is involved in signal transduction through a variety of intracellular molecules, which initiate chain reactions by activating key downstream substances including mitogen-activated protein (MAP) kinase, phosphatidylinositol 3-kinase (PI-3K), and seric-threonine kinase (AKT). Promote cell proliferation and differentiation and inhibit cell apoptosis. The overexpression of HER2 protein is associated with the development and development of many epithelial cancers. The positive rates of HER2 expression are 20-30% in breast cancer, 15% in gastric cancer, 23-80% in bladder cancer and 9-38 percent in ovarian cancer. HER2 positive indicates a dangerous disease, a high recurrence rate, easy metastasis and short survival period.

Currently, several drugs targeting HER2 have been approved worldwide. Lapatinib (Lapatinib, Tykerb), neratinib (Neratinib, Nerlynx), trastuzumab (Trastuzumab, Herceptin, Herceptin), Pertuzumab (Perjeta) and the antibody-drug conjugate (ADC) T-DM1 (Kadcyla). Among them, trastuzumab and lapatinib have been approved by CFDA in China for marketing. The antibody-conjugated drug T-DM1, consisting of trastuzumab conjugated with the tubulin polymerization inhibitor DM1, was approved by the FDA in 2013 for the treatment of HER2-positive metastatic breast cancer that progressed on Herceptin and Paclitaxel. The results of the registration phase III clinical EMILIA trial showed that T-DM1 significantly prolonged progression-free survival and overall survival compared with lapatinib plus capecitabine, with lower toxicity. However, T-DM1 has not yet been marketed in China.

**1.2 Introduction to Study Drug**

The FS-1502 developed by Fosun is a new generation of site-directed coupling cleavage antibody-conjugated drugs, which are site-directed coupling linkers and cytotoxins at the end of the light chain of monoclonal antibodies. It is more stable and controllable in production process and quality. FS-1502 cut off the β-glucuronidase in the acidic environment of tumor cells, thus releasing the active tubulin inhibitor MMAF from FS-1502 completely, and jointly exerting the tumor inhibitory activity. The FS-1502 thus has the property of being fixed and cleavable.

Preclinical test data showed that FS-1502 has obvious high tumor tissue distribution characteristics, can specifically release toxin MMAF in HER2-positive tumor tissue, kill tumor cells, and has sufficient safety window, the safety risk of rapid climbing in clinical dose exploration stage is controllable.

**1.2.1 FS-1502 specifically releases toxin MMAF in HER2-positive tumor tissues**

FS-1502 binds to HER2 on the membrane of tumor cells and is hydrolyzed by -glucuronidase in the lysosome after endocytosis, releasing the toxin MMAF. The enzyme needs acidic conditions to be active (see Fig. 1-1). Thus, FS-1502 rarely releases the toxin MMAF in the neutral PH environment of normal tissues.β

Figure 1 - 1 Activity of **β-**glucuronidase under different PH conditions

This was also confirmed in preclinical testing: no MMAF formation was detected in FS-1502 (1 mg/mL) incubated with normal human hepatocytes at 37oC (study 405186-2017122701-MID); FS-1502 (10 g/mL) was incubated with HER2-positive tumor cells BT-474 at 37oC and lyzed to produce MMAF (Study 405186-2017122703-MID); Intracellular concentrations of MMAF released by incubation of FS-1502/LCB14-0110 (1g/mL) with HER2-positive tumor cells SK-BR3 cells at 37oC (see Figure 1-2) (LCB14-0110-20160520-CTR study). Therefore, it can be preliminarily concluded that FS-1502 releases MMAF in HER2-positive tumor cells relatively specifically, but not in normal cells.μμ


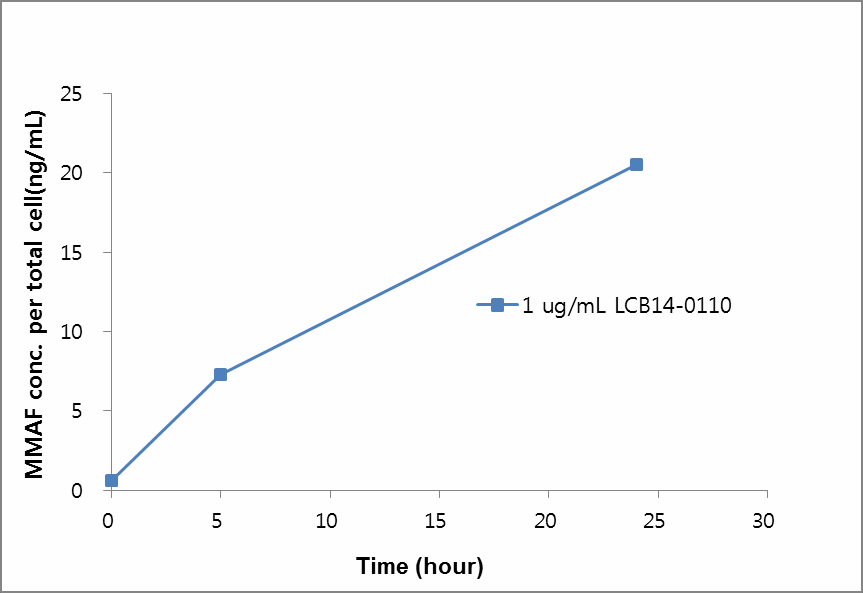


**Figure 1-2 FS-1502/LCB14-0110 (1μg/mL) Intracellular concentration of MMAF released by incubation with HER2-positive tumor cell SK-BR3 cells at 37℃**

**1.2.2 FS-1502 has sufficient safety windows**

According to the lowest onset dose obtained in JIMT-1 tumor-bearing mice was 0.5 mg/kg and the highest non-severe toxic dose in cynomolgus monkeys was 2.5 mg/kg, The safety window calculated by the animal plasma AUC calculation method (AUCHNSTD/AUCMED) for FS-1502 is approximately 6-fold, suggesting that FS-1502 has an adequate safety window for clinical studies: see Table 1-1.

**Table 1-1 Dosing Trials of FS-1502 in JIMT-1 Tumor-bearing Mice and Cynomolgus Monkeys**


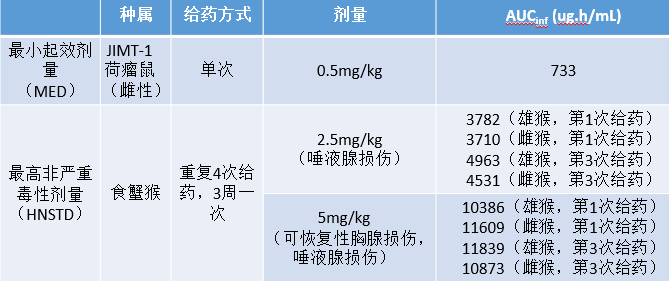


**1.2.3 FS-1502 has obvious characteristics of high distribution of tumor tissue:**

The distribution of FS-1502 in BT474 tumor-bearing mice (without thyroid gland) showed that FS-1502 was mainly distributed in spleen and tumor tissues 72 hours after entering tumor-bearing mice, and in tumor tissues 14 days after entering tumor-bearing mice (see Fig. 1-3).


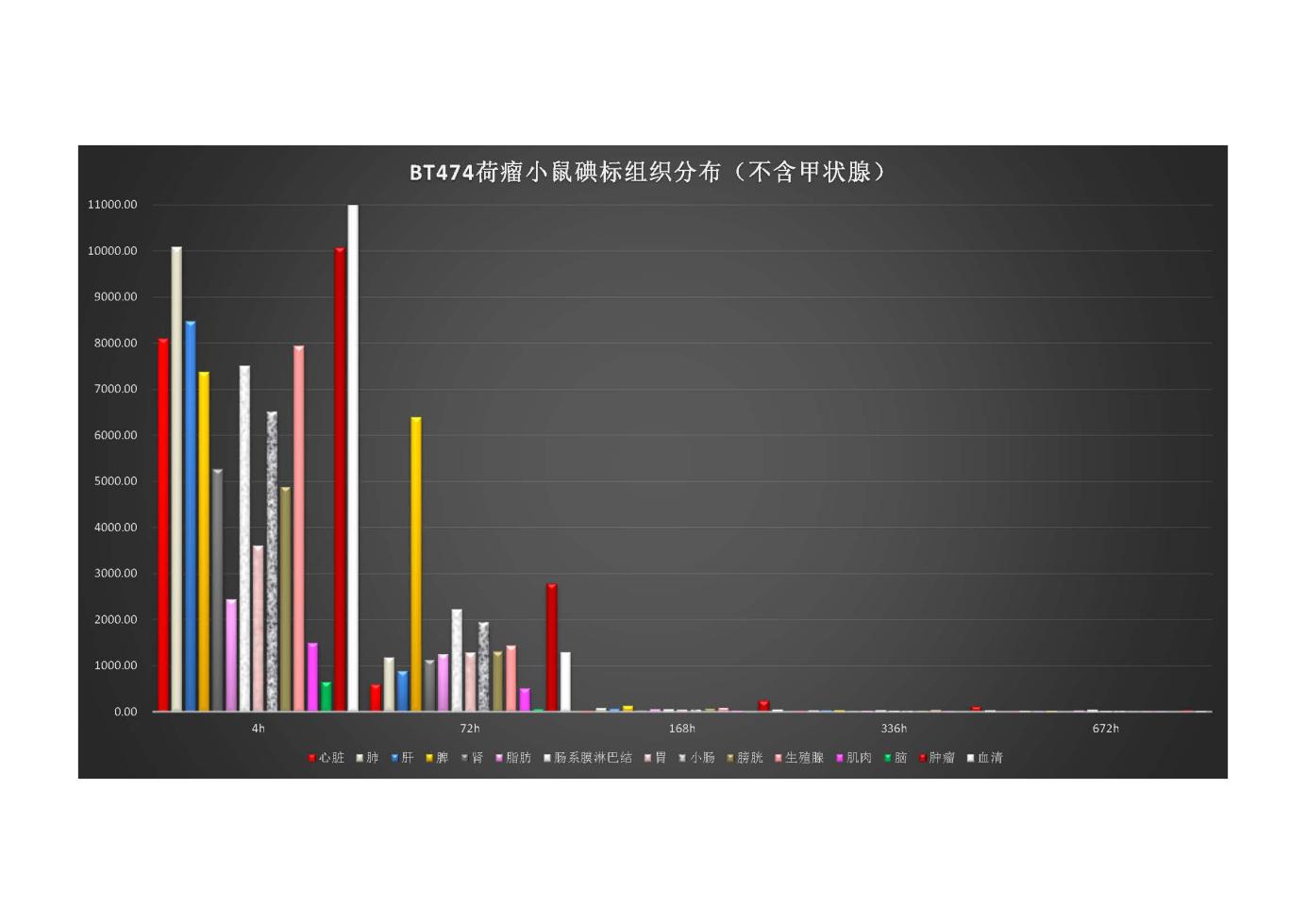


**Fig. 1-3 Distribution of Iodine Labelled FS-1502 in BT474 Tumor-bearing Mouse Tissue (without Thyroid)**

Literature data (from: Genetics and Molecular Research 13 (3): 6804-6812 (2014)) indicate that iodized isotope-labeled trastuzumab (Herceptin) is mainly distributed in the kidney, tumor, liver, and spleen at 96 hours in mice (see Table 1-2). FS-1502 also showed higher distribution in spleen before 96 h, but lower distribution in kidney and liver. The toxicological test of FS-1502 cynomolgus monkey showed no obvious toxic and side effect on spleen.

Monkey, 2.5 mg/kg, 9 weeks administration and 6 weeks recovery euthanasia: spleen 1 male monkey with enlarged spleen. (1) Slight hyperplasia of red pulp in male monkey;

Monkeys, 5 mg/kg, euthanized after 9 weeks of dosing: 2 male monkeys, 1 female monkey with enlarged spleen. The number of lymphocytes in white pulp of female was moderately reduced, and the number of red pulp in all genders was slightly to slightly hyperplasia.

Monkeys, 5 mg/kg, euthanized after recovery at 6 weeks after 9 weeks of dosing: 1 male monkey with enlarged spleen. slight to mild hyperplasia of red pulp in 1 male monkey and 1 female monkey

Monkey, 10 mg/kg, Dead anatomy: Decreased number of white pulp lymphocytes, hyperplasia of red pulp.

**Table 1-2 Distribution of Iodine-labeled trastuzumab (Herceptin) in BT474 tumor-bearing mice**

**1.3 Preclinical pharmacological studies**

**1.3.1 In vitro pharmacodynamic studies**

FS-1502 is an antibody-conjugated drug formed by the trastuzumab biosimilar HLX45-mAb, which is linked by enzyme-linked and coupled to a small molecule Linker-MMAF (LCB14-0645) with a toxin MMAF. In vitro pharmacodynamic studies, FS-1502 and HLX45-mAb, Linker-MMAF, and HLX45-mAb combined with Linker-MMAF were used in 8 tumor cell lines: breast cancer BT-474 and lung cancer Calu-3. HCC1954, JIMT-1, NCI-N87, SK-BR-3, SK-OV-3, MDA-MB-468, and T-DM1 are selected, Herceptin and Paclitaxel were used as reference test articles.

The results of two rounds of experiments showed that FS-1502 had obvious dose-dependent inhibitory effect in 7 (BT-474, Calu-3, HCC1954, JIMT-1, NCI-N87, SK-BR-3, SK-OV-3) cells. However, it did not inhibit the proliferation of MDA-MB-468 breast cancer cells. In addition, HLX45-mAb and Linker-MMAF alone or in combination did not inhibit the proliferation of these 8 cells.

The reference test substance T-DM1 showed some dose-dependent inhibition of (BT-474, HCC1954, NCI-N87, SK-BR-3, SK-OV-3, MDA-MB-468) in 6 of these cells, while it had no obvious antiproliferative effect in Calu-3 and JIMT-1 cells. The reference test substance paclitaxel showed relatively obvious dose-dependent inhibition of (BT-474, Calu-3, HCC1954, JIMT-1, NCI-N87, SK-BR-3, SK-OV-3, MDA-MB-468) in these 8 cell lines.

**1.3.2 In vivo pharmacodynamic studies**

Using a subcutaneous xenograft tumor mouse model, six different tumor strains were selected, including HER2-positive breast cancer BT-474, breast cancer HCC1954, gastric cancer NCI-N87, ovarian cancer SK-OV3, and breast cancer JIMT-1 tumor strain, Two rounds of confirmatory experiments were conducted on the efficacy of FS-1502 and HER2-negative MDA-MB-468 breast cancer tumor strain. Meanwhile, the HLX45-mAb and the linker-toxin Linker-MMAF of the monoantibody fraction of FS-1502 were treated separately and in combination to compare the differences with FS-1502. Paclitaxel, Herceptin and T-DM1 were studied as comparators in the experiment. The results of the two rounds of experiments were basically consistent. FS-1502 showed antitumor activity against HER2-positive tumor strains, but not against HER2-negative MDA-MB-468 breast cancer strains.

1. **Subcutaneous xenograft of human breast cancer BT-474 cells**

The mean tumor volume in the vehicle group was 1,064 mm3 21 days after the start of the experiment. Compared with the vehicle control group, FS-1502 significantly inhibited tumor growth at 1, 2, and 5 mg/kg, with mean tumor volumes of 299, 166, and 2 mm3, respectively, on day 21 postdose. T/C was 28.09%, 15.55% and 0.21%, TGI was 84.28%, 98.95% and 116.47%, respectively, p values were 0.002, < 0.001 and < 0.001, respectively. The efficacy of FS-1502 was significantly better than that of T-DM1 (T/C = 55.04%, TGI = 53.67%, p = 0.088) at 5 mg/kg, and the p value of FS-1502 was 0.030. Equivalent to T-DM1 at 15 mg/kg (T/C = 0.30%, TGI = 116.82%, p < 0.001).

The monoclonal antibody HLX45-mAb was given at 5 mg/kg (T/C = 58.37%, TGI = 48.98%, p = 0.055). Linker-MMAF was dosed alone at 0.09 mg/kg (T/C = 81.69%, TGI = 21.51%, p = 0.455); The (T/C = 57.82%, TGI = 49.69%, p = 0.066) mean tumor volume was 619, 869 and 612 mm3 in the HLX45-mAb and Linker-MMAF groups, respectively. The antitumor activity of FS-1502 was significantly lower than that of FS-1502.

The mean tumor volume of (T/C = 59.18%, TGI = 49.51%, p = 0.045) was 448 and 610 mm3 in the control Herceptin 5 mg/kg (T/C = 43.71%, TGI = 67.25%, p = 0.010) and Paclitaxel 15 mg/kg (T/C = 59.18%, TGI = 49.51%, p = 0.045) groups, respectively. The antitumor activity of FS-1502 was significantly lower than that of FS-1502 at 5 mg/kg.

1. **Subcutaneous xenograft tumor of human breast cancer HCC1954 cells**

The tumor volume of the vehicle control tumor-bearing mice reached 1,140 mm3 at 28 days after the start of the experiment. FS-1502, (T/C = 1.59%, TGI = 113.75%, p = 0.001) at 5 mg/kg, (T/C = 16.35%, TGI = 96.69%, p = 0.002) at 1 mg/kg, and (T/C = 27.79%, TGI = 83.50%, p = 0.002) at 0.5 mg/kg had significant tumor inhibitory effects compared with the vehicle control group, with tumor volume of 18,186 and 316 mm3, respectively. FS-1502 was as effective as positive comparator T-DM1 (T/C = 5.71%, TGI = 108.89%, p = 0.001) at 5 mg/kg. Test Article HLX45-mAb, (T/C = 26.63%, TGI = 84.93%, p = 0.002) in the 5 mg/kg group, HLX45-mAb, 5 mg/kg and Linker-MMAF, (T/C = 44.89%, TGI = 63.77%, p = 0.012) in the 0.09 mg/kg combination group and Linker-MMAF, Tumor volumes for (T/C = 63.59%, TGI = 42.24%, p = 0.084) were 301 mm3, 511 mm3, and 723 mm3 at 0.09 mg/kg, respectively. The antitumor activity of FS-1502 was significantly lower than that of FS-1502.

The control drugs Herceptin 5 mg/kg group (T/C = 27.92%, TGI = 83.40%, p = 0.002) and Paclitaxel 15 mg/kg group (T/C = 44.72%, TGI = 64.04%, p = 0 .043) also showed significant tumor inhibition compared with the vehicle control group, tumor volume was 317 and 508 mm3 respectively. The antitumor activity of FS-1502 was significantly lower than that of FS-1502 at 5 mg/kg.

1. **Subcutaneous xenograft tumor of human gastric cancer NCI-N87 cells**

The tumor volume of the vehicle control tumor-bearing mice reached 1,526 mm3 35 days after the start of dosing. Compared with the vehicle control group, the test article, FS-1502, significantly inhibited tumor growth at both 2 and 5 mg/kg, with mean tumor volumes of 424 and 87 mm3, respectively, on Day 35 postdose. T/C was 27.84% and 5.71%, TGI was 80.28% and 104.80%, respectively, p values < 0.001. FS-1502 was significantly more potent than the equivalent dose of positive comparator T-DM1 (T/C = 16.79%, TGI = 92.50%, p <0.001) at 5 mg/kg (p = 0.004). The mean tumor volume of FS-1502 was 957 and 823 mm3 at (T/C = 62.54%, TGI = 41.49%, p = 0.001) at 0.5 mg/kg and (T/C = 54.00%, TGI = 51.19%, p < 0.001) at 1 mg/kg, respectively.

The monoclonal antibody HLX45-mAb was administered at a dose of 5 mg/kg alone (T/C = 63.51%, TGI = 40.62%, p = 0.004), The mean tumor volume at the end of the experiment was 969 mm3 for (T/C = 77.38%, TGI = 24.95%, p = 0.093) and (T/C = 56.36%, TGI = 48.55%, p = 0.001) for Linker-MMAF alone at 0.09 mg/kg and HLX45-mAb combined with Linker-MMAF, respectively. 1,184 mm3 and 860 mm3. The antitumor activity of FS-1502 was significantly lower than that of FS-1502.

The mean tumor volume for the comparator Herceptin 5 mg/kg group (T/C = 63.12%, TGI = 40.87%, p = 0.002) and paclitaxel 15 mg/kg group (T/C = 47.96%, TGI = 57.94%, p <0.001) was 966 and 731 mm3, respectively. The antitumor activity was significantly lower than that of FS-1502 at 5 mg/kg.

1. **Subcutaneous xenograft tumor of human ovarian cancer SK-OV-3 cells**

The tumor volume in the vehicle control group tumor-bearing mice reached 1,270 mm3 at 28 days after the start of dosing. FS-1502, 5 mg/kg group (T/C = 5.35%, TGI = 107.48%, p < 0.001), 2 mg/kg group T/C = 21.71%, TGI = 88.84%, p < 0.001) and 1 mg/kg group (T/C = 55.99%, TGI = 49.96%, p = 0.011) had a significant antitumor effect compared with the vehicle control group, Tumor volume was 68, 277 and 711 mm3, respectively. FS-1502 at 5 mg/kg was superior to that of the positive comparator T-DM1 at 5 mg/kg (T/C = 11.88%, TGI = 100.06%, p < 0.001)) with a p-value of 0.042. For FS-1502, the tumor volume at 0.5 mg/kg (T/C = 85.38%, TGI = 16.53%, p = 0.258) was 1,085 mm3 on Day 28 postdose.

HLX45-mAb, (T/C =57.91%, TGI = 47.62%, p = 0.014) in the 5 mg/kg group, HLX45-mAb, 5 mg/kg and Linker-MMAF, (T/C = 63.24%, TGI = 41.41%, p = 0.045) in the 0.09 mg/kg combination group, and Linker-MMAF, Tumor volumes for (T/C = 88.68%, TGI = 12.44%, p = 0.529) were 738 mm3, 808 mm3, and 1,132 mm3 at 0.09 mg/kg, respectively. The antitumor activity of FS-1502 was significantly lower than that of FS-1502.

The tumor volume of the control drug Herceptin 5 mg/kg (T/C = 41.26%, TGI = 66.50%, p < 0.001) and Paclitaxel 15 mg/kg (T/C = 46.60%, TGI = 60.62%, p = 0 .001) was 527 mm3 and 592 mm3, respectively. The antitumor activity of FS-1502 was significantly lower than that of FS-1502.

1. **Subcutaneous xenograft of human breast cancer MDA-MB-468 cells**

FS-1502 did not exhibit antitumor effects in the MDA-MB-468 xenograft model, and T-DM1 and Herceptin did not exhibit tumor inhibitory effects at 5 mg/kg.

In the first experiment, 28 days after the start of treatment, the tumor volume of the vehicle control tumor-bearing mice reached 532 mm3. FS-1502, (T/C = 103.77%, TGI = -5.61%, p = 0.830) at 5 mg/kg, (T/C = 88.05%, TGI = 16.77%, p = 0.319) at 2 mg/kg, and (T/C = 93.16%, TGI = 9.57%, p = 0.676) at 1 mg/kg did not inhibit tumor compared to vehicle control, with tumor volumes of 554, 470 and 497 mm3, respectively. The tumor volume of (T/C = 14.57%, TGI = 121.47%, p < 0.001) in the positive control drug Paclitaxel 15 mg/kg group was 78 mm3, which showed a significant tumor inhibition effect compared with the vehicle control group.

HLX45-mAb, 5 mg/kg plus Linker-MMAF, 0.09 mg/kg (T/C = 88.27%, TGI = 16.65%, p = 0.395), HLX45-mAb, 5 mg/kg (T/C = 114.72%, TGI = -20.82%, p= 0.350) and Linker-MMAF, Tumor volumes for (T/C = 116.03%, TGI = -22.73%, p = 0.297) were 470, 610, and 617 mm3 at 0.09 mg/kg, respectively. T-DM1, T-DM1, 5 mg/kg group (T/C = 86.36%, TGI = 19.26%, p = 0.407) and Herceptin 5 mg/kg group (T/C = 84.20%, TGI = 22.54%, p = 0.207) tumor volume of 461 and 448 mm3, respectively, did not significantly inhibit tumor compared with the vehicle control group.

1. **Subcutaneous xenograft tumor of human breast cancer JIMT-1 cells**

FS-1502 had a significant antitumor effect on the JIMT-1 xenograft model, and FS-1502 was significantly more potent than T-DM1 at 5 mg/kg than the positive control drug at 15 mg/kg.

In the first experiment, 28 days after the start of treatment, the tumor volume of the vehicle control tumor-bearing mice reached 1,671 mm3. FS-1502, (T/C =40.85%, TGI = 65.25%, p < 0.001) at 0.5 mg/kg, (T/C = 10.21%, TGI = 101.01%, p < 0.001) at 1 mg/kg, and (T/C = 2.22%, TGI = 110.19%, p < 0.001) at 5 mg/kg showed significant tumor inhibition compared with the vehicle control group, with tumor volumes of 709 mm3, 176 mm3, and 38 mm3, respectively. FS-1502 was significantly more potent at 5 mg/kg than the positive comparator T-DM1 (T/C= 32.08%, TGI= 75.46%, p < 0.001) at 15 mg/kg.

HLX45-mAb, 5 mg/kg alone (T/C = 63.43%, TGI = 39.55%, p < 0.001), HLX45-mAb, 5 mg/kg and Linker-MMAF, 0.09 mg/kg combination (T/C = 61.95%, TGI = 41.30%, p = 0.001) and Linker-MMAF, Tumor volumes for (T/C = 92.58%, TGI = 4.78%, p = 0.635) were 1,088 mm3, 1,062 mm3, and 1,607 mm3, respectively, in the 0.09 mg/kg alone group. The antitumor activity of FS-1502 was significantly lower than that of FS-1502.

The tumor volume of the control drug Herceptin 5 mg/kg (T/C = 57.14%, TGI = 46.61%, p < 0.001) and Paclitaxel 15 mg/kg (T/C = 53.96%, TGI = 50.03%, p < 0.001) was 984 mm3 and 934 mm3 respectively. The antitumor activity was significantly lower than that of FS-1502 at 5 mg/kg.

**1.4 Preclinical toxicology studies**

**1.4.1 Acute toxicity test**

The acute toxicity experiments of FN-1502 were studied in both rats and cynomolgus monkeys and the results are described in the following sections.

**1.4.1.1 Rats**

FS-1502 was administered as a single intravenous infusion to SD rats at doses of 20, 40, and 80 mg/kg. Animal body weight and food consumption were measured regularly for 21 days of clinical observation, and the animals were euthanized on 21 days after administration. FS-1502 At 80 mg/kg, a female rat was found dead on Day 7 with bilateral adrenal enlargement.

Weakness in hind limbs was observed in 1 female rat in the 40 mg/kg dose group. Body weight of male animals decreased on D7-D20 and body weight gain decreased on W1-W3. Decreased body weight and decreased body weight gain on W2 were observed in female rats. The food consumption of male rats was significantly reduced from 7th to 14th day. After euthanasia, 2/5 male rats and 2/5 female rats showed bilateral kidney discoloration, yellowish brown and spleen enlargement.

In the 80mg/kg group, hindlimb weakness (3/5), fluffy coat (5/5) and bowed back (4/5) were observed successively. One female, D7, was found dead, and the surviving female showed successively bowed back (4/4), fluffy coat (4/4), emaciation (1/4), bowed back (4/4), hindlimb weakness (2/4), and hindlimb swelling (1/4). The body weight of male rats decreased, and the body weight gain of W1 and W2 decreased. Body weight gain in female rats decreased on W1 and increased on W2. The consumption of D1-D7 and D7-D14 in male rats was significantly reduced. The consumption of D1-D7 in female rats was significantly reduced. In 5/5 male animals and 3/5 female animals, bilateral kidney discolored, yellowish brown and enlarged spleen were seen.

Histological changes corresponding to the yellowish-brown appearance of bilateral kidneys were characterized by mesangial proliferative glomerulonephritis under the microscope, Hyaloid casts, tubular degeneration/regeneration, tubular dilation, interstitial inflammatory cell infiltration, mitotic phase of tubular epithelial cells. Histological changes corresponding to splenomegaly were diffuse extramedullary hematopoiesis in the spleen.

The maximum tolerated dose (MTD) was 40 mg/kg.

**1.4.1.2 Cynomolgus Monkey**

Cynomolgus monkeys were given FS-1502 by intravenous infusion at doses of 3, 6, and 12 mg/kg, respectively. FS-1502 was administered as a single intravenous infusion to cynomolgus monkeys and observed for 21 days. Animals were euthanized on D21. Clinical observation, body weight, body temperature, electrocardiogram, blood pressure, blood oxygen saturation and clinical pathology were performed during the test. (Hematology, Chemistry, Hemagglutination and Urinalysis) 2. Gross anatomy and histopathology were examined.

FS-1502 middle dose group (6mg/kg): Soft stool was seen in male animals D7-D8; FS-1502 high dose group (12 mg/kg): thinning coat began to be seen in male animal D7; During the trial, treatment-related changes were observed in hematological parameters in each FS-1502 dose group, mainly increased Mono, LUC, and Retic, and decreased PLT. In FS-1502 groups, coagulation function indexes were significantly changed, mainly FIB indexes were increased. In FS-1502 groups, blood biochemical indexes were significantly increased, including ALT, AST, ALP, GGT, CK, TBil and LDH. For FS-1502 at doses ≥ 6 mg/kg, test article-related gross lesions were small thymus with corresponding thymic atrophy microscopically. In addition, red pulp hyperplasia and erosion of gastric mucosa were seen in the spleen at 12 mg/kg, but it was not possible to determine whether it was related to FS-1502.

Maximum tolerated dose (MTD) was 12 mg/kg.≥

**1.4.2 Long-term toxicity test**

FS-1502 was administered to SD rats by repeated intravenous infusion at doses of 10, 20 and 40 mg/kg, once every 3 weeks for 9 weeks. Animals in each group died, and body weight gain in animals at doses of 20 mg/kg or more was reduced. Neut, Mono and LUC were increased in animals treated with ≥10 mg/kg, PT was prolonged in animals treated with ≥20 mg/kg, FIB was increased in animals treated with ≥10 mg/kg, and Alb and A/G were decreased in males treated with ≥20 mg/kg. Elevated UREA and Cre in males at 40 mg/kg, elevated CHO in animals at ≥10 mg/kg, elevated TG in animals at ≥20 mg/kg, and elevated urinary protein and white blood cells in animals at ≥10 mg/kg. The weights of the main bronchus and spleen in the lungs and spleens were increased at 10 mg/kg or more, the weights of the livers in males and females at 10 mg/kg or more, and the weights of the kidneys in the 40 mg/kg or more. Testis and epididymis weights decreased in males at or above 10 mg/kg and thymus weights decreased in females at 40 mg/kg. The main toxic target organs were bone marrow, reproductive organs (testis, epididymis and breast), kidney, liver, lung, stomach, thymus, and teeth. The major toxicities of FS-1502 were similar to those of MMAF, except for changes in the lung.

FS-1502 was administered to SD rats by intravenous infusion at 10, 20, and 40 mg/kg. The mean peak concentrations and mean AUC of FS-1502, total antibody, and MMAF increased with increasing dose after the first and third doses. After the third dose, the systemic exposures of FS-1502, total antibody and MMAF were not significantly increased in the high dose group.

FS-1502 was administered to SD rats by intravenous infusion at 10, 20, and 40 mg/kg. Systemic exposure in females was slightly higher than that in males after the first dose, FS-1502, total anti- and MMAF after the first and third doses, except that in females at 10 mg/kg. There was no significant gender difference in systemic exposure (P>0.05).

FS-1502 was administered to SD rats by intravenous infusion at doses of 10, 20, and 40 mg/kg, once every 3 weeks. After three consecutive doses, the systemic exposure of FS-1502 and total antibody in animals was higher than that in low-dose male animals. The total anti-accumulation in other animals was not obvious. The accumulation of MMAF in animals decreased with increasing dose. The conjugated toxin MMAF was administered to SD rats by intravenous infusion at a dose of 10 mg/kg, once every 3 weeks, and after 3 consecutive administrations, the systemic exposure accumulated slightly.

Systemic exposure to FS-1502 in Sprague-Dawley rats by intravenous infusion at doses of 10, 20, and 40 mg/kg was generally consistent with systemic exposure to total antibodies.

FS-1502 was administered to cynomolgus monkeys by repeated intravenous infusions at doses of 2.5, 5, and 10 mg/kg every 3 weeks for 9 weeks. Animals in the 10 mg/kg group died, and body weight gain decreased in the 2.5 mg/kg or more group. Food consumption decreased in 10 mg/kg, QTc increased in 10 mg/kg, Mono and LUC increased in 2.5 mg/kg or more, and HGB decreased in 10 mg/kg males. HGB decreased in females at 5 and 10 mg/kg, RBC decreased in females at 10 mg/kg, HCT decreased in females at 5 and 10 mg/kg, and Retic increased in males at 10 mg/k, PLT was increased in males at or above 5 mg/kg, PT and APTT were increased in 10 mg/kg, and AST, CK and LDH were increased in females and males at or above 2.5 mg/kg. ALP and GGT were increased in all genders animals at 10 mg/kg, Alb decreased in all genders animals at 5 mg/kg or more, and Glb increased and A/G decreased in all genders animals at 2.5 mg/kg or more. Cre increased in males at 10 mg/kg, urinary protein increased in females at 2.5 mg/kg or more, IL-6 increased in 10 mg/kg, spleen and liver weight increased and thymus weight decreased in 2.5 mg/kg or more. Adrenal gland, bone marrow (femur and sternum), male reproductive system (testis, epididymis, prostate, and seminal vesicle glands), gallbladder, heart, gastrointestinal tract (colon, duodenum, jejunum, rectum, and stomach), kidney, liver, lung and bronchus, lymph nodes (groin, mesentery), salivary glands (submandibular, parotid), spleen, thymus, and bladder were found with test article-related lesions.

Based on the results of the repeated-dose toxicity test of FS-1502 in preclinical cynomolgus monkeys, the highest non-serious toxic dose was considered by the tester and Fosun to be 5 mg/kg. Following discussion with the CDE, the clinical phase used cynomolgus monkey 2.5 mg/kg as the highest non-serious toxic dose to calculate the human clinical starting dose.

**1.4.3 Antidrug Antibody**

A total of 228 samples from 38 rats undergoing a 9-week repeat-dose toxicity test were screened and analyzed, and 9 screening positive samples were confirmed. Five of these samples (from 2 animals each) were confirmed to be specific for anti-FS-1502 and anti-HLX45-mAb antibodies and titers were determined.

No positive antibody was detected in any animal in 10mg/kg and 20mg/kg groups. Two animals in the 40 mg/kg group were detected, and 18-644 animals were detected as positive antibodies on D42 and D63, respectively, with titers ranging from 32 to 64. Due to the late death of the animal, the 18-649 animal collection was replaced, and no positive antibodies were subsequently detected. The 18-654 animals detected positive antibodies on D42, D63 and D106, but titers were low, between <1 and <2.

Overall, FS-1502 was administered to animals by intravenous infusion, and antibodies were detected in the animals. With the increase of the number of drug administrations, the detection rate and titer of positive antibody samples increased, but the overall positive rate was low, which may be related to the higher concentration in animals and the lower drug resistance of the test method.

A 6-week recovery trial for 9-week repeat-dose administration of FS-1502 or HLX45-mAb to cynomolgus monkeys by intravenous infusion, screened 240 samples from 50 cynomolgus monkeys and confirmed 23 screening positive samples, nine samples (from 5 animals each) confirmed specific antibodies against FS-1502 or HLX45-mAb.

In the 2.5 mg/kg FS-1502 dose group, animal No. 1726253 detected antibodies at D22, D43, and D64 in a rate of 1 in 10, with a titer of 16, 64, and 256 on D64. No positive antibodies were detected in the 5 mg/kg FS-1502 group, and the detection rate was 0. In the 10 mg/kg FS-1502 dose group, 1726272 animals detected positive antibodies on D22 and 1726274 on D43, respectively, and no further samples were collected due to the late death of this animal. The titer ranges between < 1 and 8.

In the 10 mg/kg HLX45-mAb group, positive antibody was detected in D43 samples of 1726289 animals with titer <1; 1726285 animal D64, D85 and D106 were positive for antibody with titers ranging from 4 to 16.

Among them, in the confirmation of the screening positive samples of each dose group of FS-1502, it was found that the confirmation results of FS-1502 and HLX45-mAb were not exactly the same, and some samples were positive for FS-1502. HLX45-mAb was confirmed negative. For animals treated with HLX45-mAb, FS-1502 and HLX45-mAb were confirmed respectively, and the results were consistent. It is suggested that antidrug antibodies against the conjugated region or small molecular fraction of FS-1502 may be produced after administration of FS-1502 to animals.

In conclusion, antibodies were detected in animals given intravenous infusion of FS-1502 or HLX45-mAb. With the increase of the number of administrations, the detection rate of positive antibody samples increased gradually, and the titer increased gradually with time.

**1.5 Preclinical Pharmacokinetic Studies**

**1.5.1 Absorption**

The AUC0-last ratios of FS-1502 were 1.25, 0.984, and 1.09 in female and male SD rats after intravenous administration at three different doses, respectively, without gender differences. The ratios of AUC0-last for total antibody after intravenous administration of FS-1502 in female and male SD rats were 1.21, 0.868 and 1.05, respectively, without gender differences. Systemic exposure (AUC0-last) of FS-1502 increased substantially dose-proportionally in male and female Sprague-Dawley rats as the intravenous dose increased from 0.5 to 3 mg/kg. MMAF was not detected in serum of male and female rats at three different doses.

MMAF was not detected in SD rat serum following a single intravenous administration of FS-1502 at 0.5, 0.1, and 3 mg/kg to SD rats (limit 20.0 pg/mL).

Following a single intravenous administration of 0.25, 1, and 2 mg/kg FS-1502 to male and female cynomolgus monkeys, the peak concentration (Cmax) of FS-1502 was 3920 ±611, 21000 ±4270, and 59100 ±12400 ng/mL, respectively. Corresponding time to peak (Tmax) were 0.525 +/- 520, 1.00 and 0.842 +/- 388 h, respectively. The serum clearance rates (CL) were 0.0384 +/- 00792, 0.0177 +/- 0.00363 and 0.00874 +/- 0.00365 mL/min/kg respectively. The steady-state apparent volume of distribution (Vdss) were 0.0964 +/- 0.210, 0.0656 +/- 0170 and 0.0490 +/- 00142 L/kg, respectively. The elimination half-life (T1/2) was 56.1±4.81, 88.3±28.5 and 110±28.3 h, respectively. The area under the serum concentration curve (AUC0-last) values from the last quantifiable time point at time 0 were 110000 ±23100, 975000 ±207000 and 4210000 ±1180000 h·ng/mL, respectively.

Systemic exposure Cmax and AUC0-last in male and female cynomolgus monkeys increased linearly with dose increasing from 0.25 mg/kg to 1 mg/kg, However, the increase in systemic exposure AUC0-last in female cynomolgus monkeys was more than dose proportional; Systemic exposure Cmax and AUC0-last in male and female cynomolgus monkeys increased linearly as the dose increased from 1 to 2 mg/kg, However, systemic exposure to AUC0-last in male cynomolgus monkeys increased more than dose-proportionally; Overall, Cmax of systemic exposure increased linearly with dose increase from 0.25 to 2 mg/kg in male and female cynomolgus monkeys, but AUC0-last increased more than dose proportionally in both male and female cynomolgus monkeys. There were no apparent gender differences in systemic exposure (AUC0-last and Cmax) in male and female cynomolgus monkeys at three different doses.

Following a single intravenous injection of 0.25, 1 and 2 mg/kg FS-1502 in male and female cynomolgus monkeys, the peak concentrations (Cmax) of total antibody were 2990 ±511, 12800 ±1600 and 36300 ±6870 ng/mL, respectively. Corresponding time to peak (Tmax) were 0.208 + + 388, 0.683 + 491 and 1.18 + 1.46 h, respectively. The serum clearance rates (CL) were 0.0704 +/- 0.211,0.0311 +/- +/- 00503 and 0.0153 +/- 0.00565 mL/min/kg respectively. The steady-state apparent volume of distribution (Vdss) were 0.0907±0.0191, 0.0793±0.00663 and 0.0586±0.0144 L/kg, respectively. The elimination half-life (T1/2) was 15.6±2.14, 39.9±8.60 and 65.0±12.7 h, respectively. The area under the serum concentration curve from time 0 to the last quantifiable time point (AUC0-last) values were 61400 ±18600, 544000 ±87600 and 2370000 ±644000 h·ng/mL, respectively. Systemic exposure Cmax and AUC0-last in male cynomolgus monkeys increased linearly with dose increases from 0.25 mg/kg to 1 mg/kg, However, the increase in systemic exposure AUC0-last in female cynomolgus monkeys was more than dose proportional; Systemic exposure Cmax and AUC0-last in male and female cynomolgus monkeys increased linearly as the dose increased from 1 to 2 mg/kg, However, systemic exposure to AUC0-last in male cynomolgus monkeys increased more than dose-proportionally; Overall, Cmax of systemic exposure increased linearly with dose increase from 0.25 to 2 mg/kg in male and female cynomolgus monkeys, but AUC0-last increased more than dose proportionally in both male and female cynomolgus monkeys. There were no apparent gender differences in systemic exposure (AUC0-last and Cmax) in male and female cynomolgus monkeys at three different doses.

MMAF was detected only in the serum of mid- and high-dose (2 mg/kg) animals after a single intravenous administration of 0.25, 1, and 2 mg/kg FS-1502 in male and female cynomolgus monkeys, and at concentrations below 20 pg/mL.

**1.5.2 Distribution**

[14C]-labeled FS-1502 was administered a single intravenous dose to rats at a dose of 15 mg/63 .mu.Ci/kg for in vivo tissue distribution studies. Blood samples and tissue tests were performed at 2 hours, 72 hours, 9 days and 42 days after administration, and the total radioactivity was widely distributed in rats, mainly in lung, liver, ovary and uterus, kidney, heart and spleen.

The total radioactivity in the test tissues and plasma reached or approached the peak concentration (Cmax) at 2 hours after administration, except for skeletal muscle, body fat, ovary uterus, testicular epididymis, and thymus, which reached or approached the peak concentration (Cmax) at 72 hours after intravenous administration. On 9 days postdose, the total radioactivity in the tissues tested and plasma, except the spleen, decreased from 16.58% (thymus) to 54.23% (intestinal wall) compared to 72 hours. At the last collection time point (42 days), the total radioactivity in skeletal muscle and whole brain was below the lower limit of detection, and the distribution of radioactivity remained in the rest of the test tissues and plasma, accounting for less than 1.53% of the dose.

**1.5.3 Metabolism**

FS-1502 was incubated in vitro with human, monkey, and rat hepatocytes for 120 minutes and the FS-1502 was stable with no metabolites detected. Linker-MMAF in vitro was incubated with human, monkey and rat hepatocytes for 120 minutes and was not stable enough to cause a mediator effect and did not produce MMAF. FS-1502 was incubated with tumor cells BT-474 for 24 hours and results showed that FS-1502 produced MMAF at tumor cells BT-474. This indicates that FS-1502 metabolizes in HER2-positive cells to produce MMAF, and also supports the mechanism of action of FS-1502, which targets HER2-positive cells and produces cytotoxins in tumor cells to suppress tumor.

After a single intravenous administration of [14C]-labeled FS-1502 to rats (15 mg/63 μCi/kg), the radioactivity in plasma was mostly composed of [14C]-labeled FS-1502 and its related protein macromolecules (non-small molecules). Compared with the retention time of the [14C]-labeled FS-1502 standard, the radioactivity in the plasma samples at 2 hours, 72 hours and 9 days was predominantly unchanged ([14C]-labeled FS-1502), and in the plasma samples at 42 days. The original drug has been markedly degraded.

MMAF was detectable in animal serum at 2.5, 5, and 10 mg/kg in cynomolgus monkeys and 10, 20, and 40 mg/kg in rats and increased with increasing doses of FS-1502.

Thus, FS-1502 slowly metabolizes MMAF in animals.

**1.5.4 Excretion**

[14C] labeled FS-1502 was administered intravenously to rats (15 mg/63 μCi/kg) and the total excretion was 19.34% of the dose between 0 and 72 hours after administration. The total amount of bile excreted accounted for 15.88% of the dose. Urine was 1.58%, feces 1.46%, cage rinse and wash solution 0.42%. The results showed that the total radioactivity was mainly excreted through bile.

The excretion rate and amount of total radioactivity were similar in all genders rats, and the total recovery was 102.10% of the dose in 052 days. Mainly excreted from feces, accounting for 84.69% of the dose. Excretion occurred primarily within 28 days after dosing and accounted for approximately 86.51% of the dose.~

Therefore, the excretion mode of drug isotopes is mainly through bile into feces, and since the isotope is marked on MMAF, it indicates that MMAF is mainly through bile into feces.

**1.6 Theoretical basis of this clinical study**

This clinical study is a multicenter, open-label, single-arm Ia dose-finding and Ib dose-expansion study to evaluate the safety, tolerability, pharmacokinetic profile of FS-1502 in patients with HER2-expressing advanced malignant solid tumors and to assess the presence of FS-1502 in HER2-positive patients. Efficacy in breast cancer patients who have failed prior trastuzumab therapy, including patients with locally advanced or metastatic breast cancer who have relapsed after more than 3 months of prior adjuvant chemotherapy.

The ICH-S9 guidelines recommend that the single-dose starting dose be calculated as 1/6 of the highest non-serious toxic dose (HNSTD) in non-rodents or 1/10 of the STD10 in odontophagophages (10% of animals exhibit a serious toxic effect). Based on the results of the FS-1502 nonclinical repeated-dose toxicology study, the monkey HNSTD is 2.5mg/kg, and the safety factor of 1/10 is used according to the FDA 2005 "Estimation of the Maximum Safe Starting Dose in Humans" The human starting dose is 0.085 mg/kg. According to the principle of dose ramping and considering the practical clinical operability, the dose is accurate to one decimal place. The final dose is set as follows: 0.1 mg/kg, 0.2 mg/kg, 0.4 mg/kg, 0.6 mg/kg, 0.8 mg/kg, 1.0 mg/kg, 1.3 mg/kg. Based on the PK, PD, and safety data from the clinical trial data, the MTD and the recommended dose for Phase II were determined by agreement between the investigator and the sponsor.

According to the lowest onset dose obtained in HCC1954 tumor-bearing mice and JIMT-1 tumor-bearing mice was 0.5 mg/kg and the highest non-severe toxic dose in cynomolgus monkeys was 2.5 mg/kg, The safety window for FS-1502 calculated using the animal plasma AUC calculation method (AUCHNSTD/AUCMED) is approximately 6-fold, indicating that FS-1502 has an adequate safety window for clinical studies.

Dose ramp will be used to enroll patients in a 3 + 3 mode, starting at 0.1 mg/kg, with the main dose tentatively set at 0.1 mg/kg, 0.2 mg/kg, 0.4 mg/kg, 0.6 mg/kg, 0.8 mg/kg, 1.0 mg/kg, 1.3 mg/kg. Upon completion of a dose-limiting toxicity (DLT) observation for a dose group, the investigator and the sponsor determined the dose level and number of enrolled patients in the next dose group based on previously available safety, efficacy, and PK data for the dose group. Or determine the MTD/appropriate exposure dose, allowing dose adjustment based on experimental data.

If the MTD is not observed at the maximum preset dose, the investigator and the sponsor will determine the appropriate exposure dose or continue the dose ramping study based on preclinical and clinical safety, PK, and efficacy data. If the MTD is reached at the starting dose, a lower dose will be considered. This study will be conducted in accordance with the protocol, Good Clinical Practice (GCP) and the regulatory requirements used.

**1.6.1 Justification for Dosing Frequency Update**

This is a multicenter, open-label, single-arm, first-in-human clinical study of FS-1502: a dose-finding study of FS-1502 in patients with HER2-expressing advanced malignant solid tumors and a dose-expansion study in patients with HER2-positive locally advanced or metastatic breast cancer. The study was based on the 3 + 3 principle, the dosage was ramped up, and the administration frequency was 4 weeks/time, with a 28-day cycle. As of December 4, 2020, 25 patients had been enrolled, with the dosage of 0.1 mg/kg (3 patients), respectively. 0.2mg/kg (4 cases), 0.4mg/kg (5 cases), 0.6mg/kg (7 cases), 0.8mg/kg (4 cases), 1.0mg/kg (2 cases) The 1.0 mg/kg dose group was still in the DLT observation period.

Currently available clinical PK data suggest that the mean half-life (t1/2) of FS-1502 at 0.1-0.6 mg/kg is 0.63-1.66 days, Concentrations of FS-1502 decreased below the limit of detection approximately 9-14 days postdose (0.6 mg/kg group). Compared with PK data in tumor-bearing mice: Clinical PK data showed that the half-life in humans was significantly shorter than in mice, supporting the change from once every 4 weeks to once every 3 weeks.

As of 05 November 2020, the clinical safety profile was good, with no patients at 0.6 mg/kg and below achieving an objective response, considering the low dose and the long dosing interval. The 0.8 mg/kg dose group completed the DLT observation period and no DLT events occurred. Therefore, the dosing frequency was adjusted while the dose ramp was continued, and from the date of the version 3.0 protocol effective (25 December 2020), newly enrolled patients were given every 3 weeks for a 21-day cycle.

**1.6.2 Determination of the recommended dose in Phase II**

As of December 30, 2021, 67 patients were enrolled, including 64 breast cancer patients and 3 non-breast cancer patients. (1 case of non-small cell lung cancer, 1 case of ampullary adenocarcinoma, 1 case of submandibular adenocarcinoma, all in 3.0mg/kg group), accumulatively, 11 dose groups were climbed. The number of patients in each dose group was: 0.1 mg/kg (3 Q4W), 0.2 mg/kg (4 Q4W), 0.4 mg/kg (5 Q4W), 0.6 mg/kg (7 Q4W), 0.8 mg/kg (4 Q4W), 1.0 mg/kg (3 Q3W), 6 Q4W), 1.3 mg/kg (4 Q3W, 6 Q4W), 1.7 mg/kg (4 Q3W, 4 Q4W, Q4W (5 cases), 2.3 mg/kg (6 cases Q3W), 3.0 mg/kg (9 cases Q3W), 3.5 mg/kg (1 case Q3W), 20 cases were treated in group.

**1.6.2.1 Phase Ia Safety Data**

As of December 30, 2021, all 67 patients had reported at least one treatment-emerging adverse event (TEAE). The majority of TEAEs were Grade 1 to 2, with the most common AEs being transaminase increased, hypokalemia, and thrombocytopenia. Grade 3 TEAEs were reported in 27 patients (40.3%), of which 22 patients (32.8%) reported Grade 3 TEAEs related to study drug, the most common being hypokalaemia (10.4%), followed by thrombocytopenia (9.0%). There were no TEAEs of Grade 4 and higher. Six patients (9.0%) reported serious TEAEs, of which 3 patients (4.5%) reported serious TEAEs related to study drug. In the 3.0 mg/kg dose group, 1 of the first 3 patients experienced a DLT (grade 2 decreased creatinine clearance) and none of the subsequent patients. The first patient in the 3.5 mg/kg dose group had a DLT event on Cycle 1 Day 7, characterized by Grade 3 thrombocytopenia with subcutaneous bleeding, and the enrollment in this dose group was stopped and the dose escalation continued, with no DLT observed in the remaining dose groups.

**1.6.2.2 Phase Ia pharmacokinetic characteristics**

In the range of 0.1-3.0mg/kg, ADC concentration increased with the increase of the dose after the first administration, and the elimination speed of the terminal drug concentration in plasma was faster in low dose group than in high dose group. The plasma concentration of MMAF was much lower than that of ADC and was close in the 1.7-3.0 mg/kg dose groups. The plasma concentration of total antibodies also increased with increasing dose, and the elimination characteristics of terminal plasma concentration at the same dose were similar to those of ADC.

FS-1502 reached its peak almost immediately after intravenous drip administration, and the mean Cmax and AUC0-21day of FS-1502 after the first (i.e., first cycle) administration were 1.25-62.71 micrograms/mL and 1.26-329.59 micrograms*day/mL, respectively The mean Cmax was linear with dose, and the mean AUC0-21day was higher than the dose-increasing ratio in the low dose range (0.1-0.8 mg/kg) and close to the dose-increasing ratio in the high dose range (1.0-3.0 mg/kg); In the third cycle, the Q3W administration of FS-1502 in the 1.3-2.3 mg/kg dose groups accumulated, and the mean AUC accumulation ratio was 1.2-1.7.

Plasma concentration of MMAF peaked approximately 1 to 7 days after administration, with exposure levels much lower than those of FS-1502, and overall plasma concentration in the 0.1 to 3.0 mg/kg dose ranged between 0.01 and 1.00 ng/mL, with low plasma concentration.

The total antibody peaked almost immediately after intravenous infusion, and the mean Cmax and AUC0-21day after the first administration were 1.28-69.35 μg/mL and 1.60-478.71 μg*day/mL, respectively. The exposure in vivo was higher than that in FS-1502, and the dose variation was consistent with that in FS-1502. In vivo accumulation after the third cycle was also similar to FS-1502.

**1.6.2.3 Phase Ia Clinical Efficacy Data**

To support the recommended dose selection, clinical efficacy in 31 breast cancer patients in four dose groups, 1.3 mg/kg, 1.7 mg/kg, 2.3 mg/kg, and 3.0 mg/kg, was analyzed as of December 30, 2021. The confirmed ORRs for the 4 dose groups were 20%, 44.4%, 66.7% (4/6) and 16.7%, respectively.

**Table 1-3 Overall best response evaluation for the four dose groups**

|  | **1.3 mg/kg(N=10)** | **1.7 mg/kg(N=9)** | **2.3mg/kg(N=6)** | **3.0 mg/kg(N=6)** |
| --- | --- | --- | --- | --- |
| **CR** | **0** | **0** | **0** | **0** |
| **PR** | 2 (20.0%) | 4 (44.4%) | 4 (66.7%) | 1 (16.7%) |
| **SD** | 2 (20.0%) | 2 (22.2%) | 2 (33.3%) | 4 (66.7%) |
| **PD** | 6 (60.0%) | 3 (33.3%) | 0 | 0 |
| **NE** | 0 | 0 | 0 | 1 (16.7%) |
| **Confirmed ORR** | 2 (20.0%) | 4 (44.4%) | 4 (66.7%) | 1 (16.7%) |

**
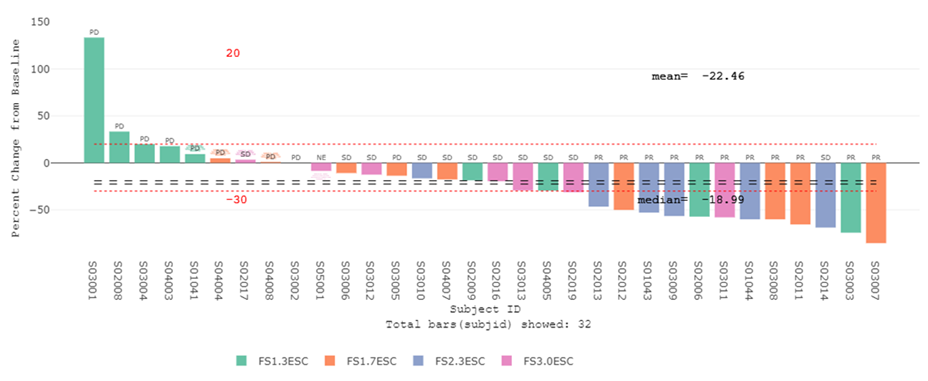
**

**Figure 1-4 Waterfall Plots of the Best Percent Change in Target Lesions Compared to the Baseline Sum of Diameters in Four Dose Groups**

Based on the above data, the SMC meeting was held on January 17, 2022, to discuss the recommended dose in Phase II. After discussion, experts at the meeting reached a consensus that the in vivo exposure of FS-1502 increased with the increase of dose, and the relationship between Cmax and dose was linear, and the relationship between clearance and dose was nonlinear. Exposure to FS-1502 was significantly associated with the incidence of TEAEs of Grade 3 or higher, and AEs of liver toxicity, thrombocytopenia, and ocular toxicity (e.g., dry eye, blurred vision) of any grade. The efficacy of the 3 mg/kg dose group was not significantly superior to that of the 2.3 mg/kg dose group, but thrombocytopenia, ocular toxicity and hepatotoxicity tended to be further increased. Therefore, based on the efficacy, safety and PK data, the efficacy of the 2.3 mg/kg dose was positive and the overall safety was tolerable and manageable. The SMC agreed to extend the dose of 2.3 mg/kg as the recommended dose for the Phase II clinical studies.

**2 Study Objectives and Endpoints**

**2.1 Objectives of the study**

**2.1.1 Phase Ia**

**Main Purpose:**

- To observe the safety and tolerability of continuous intravenous infusion of FS-1502 single agent in patients with advanced HER2-expressing malignant solid tumors.
- To determine the maximum tolerated dose (MTD) of FS-1502 monotherapy in patients with advanced HER2-expressing solid tumors, the recommended phase 2 dose (recommend phase 2 dose, RP2D) and dose-limiting toxicity (DLT).

**Secondary Objectives:**

- To investigate the antitumor activity of FS-1502 single-agent administered continuously by intravenous infusion in patients with advanced HER2-expressing malignant solid tumors.
- To observe the pharmacokinetics (PK) characteristics and immunogenicity of FS-1502 single drug administered continuously intravenously in patients with advanced HER2-expressing malignant solid tumors.

**2.1.2 Phase Ib**

**Main Purpose:**

- To evaluate the efficacy of continuous intravenous infusion of FS-1502 single agent in patients with HER2-positive locally advanced or metastatic breast cancer.

**Secondary Objectives:**

- To evaluate the safety and tolerability of continuous intravenous infusion of FS-1502 single agent in patients with HER2-positive locally advanced or metastatic breast cancer.
- To evaluate the population pharmacokinetics (Pop PK) profile and immunogenicity of continuous intravenous infusion of FS-1502 single agent in patients with HER2-positive locally advanced or metastatic breast cancer.

**2.2 Study Endpoints**

**2.2.1 Phase Ia**

**Primary Endpoint:**

- Safety: occurrence of dose limiting toxicities (DLTs) in the first cycle of treatment after the first dose of FS-1502 alone.
- Efficacy: objective response rate (ORR).

**Secondary Endpoints:**

- Other safety endpoints:
- Type and frequency of adverse events (AEs) occurring during the treatment period, with toxicity grade evaluated according to the National Cancer Institute Common Toxicity Criteria for Adverse Events version (national cancer institute common terminology criteria for adverse events, NCICTCAE) 5.0;
- Serious adverse events (SAEs) and toxicities leading to permanent discontinuation during treatment;
- Frequency and cause of death within 30 days after last dose;
- Laboratory safety examination results graded according to NCICTCAE version 5.0;
- Changes in vital signs and ECOG scores.
- Other efficacy endpoints were progression free survival (PFS), overall survival (OS), 1-year OS rate, duration of response (DOR), and clinical benefit (CBR).
- PK parameters for FS-1502, total antibody, and MMAF.
- The occurrence of anti-drug antibodies (ADA) and neutralizing antibodies (NAB) for FS-1502.

**2.2.2 Phase Ib**

**Primary Endpoint:**

- ORR

**Secondary Endpoints:**

- Other efficacy endpoints: PFS, DOR, and CBR, and 1-year OS rate and OS.
- Type and frequency of AEs, toxicity grade evaluated according to NCI CTCAE version 5.0; SAEs occurring during the study and toxicities leading to permanent discontinuation;
- Frequency and cause of death within 30 days after last dose;
- Laboratory safety test results graded according to NCI CTCAE version 5.0.
- PK Parameters for FS-1502, Total Antibodies, and MMAF.
- FS-1502 Anti-Drug Antibody (ADA) and Neutralizing Antibody (NAb).

**2.3 Definitions of Study Endpoints:**

DLTs were defined as toxic events occurring during the DLT observation period (21 days/28 days after the first dose).

The MTD was defined as a DLT event observed in 33% of patients with evaluable DLT events. (i.e. up to 1 out of 6 patients or 0 out of 3 patients) The highest dose of.<

ORR is defined as the proportion of patients with a confirmed complete response (CR) or partial response (PR) as assessed by RECIST version 1.1.

PFS is defined as the time from the start of study drug until disease progression or death, whichever occurs first. Patients without an event (no progression or death) will be cut off from the date of last tumor evaluation. For patients missing post-baseline efficacy assessments, the date of first dose was used as the cut-off point.

OS was defined as the time from the start of study drug administration to death from any cause. For patients with no observed death event, the date on which the patient's survival information was last available will be used as the cut-off point.

DOR is defined as the time from the first CR or PR to tumor progression or death from any cause, whichever occurs first. If there is no tumor progression or death, the date of last tumor evaluation will be used as the cut-off point.

The 1-year OS rate is defined as the proportion of patients who are alive within 1 year after the start of dosing.

DCR is defined as the proportion of patients with confirmed CR, PR, and SD lasting ≥ 6 weeks.

The CBR is defined as the proportion of patients with confirmed CR, PR, and SD lasting ≥ 24 weeks as evaluated by RECIST version 1.1.

**3 Study plan**

**3.1 Overall Study Plan**

This is a single-arm, open-label, nonrandomized, dose-escalation, and dose-expansion phase I clinical study to evaluate the safety, tolerability, pharmacokinetic profile, and preliminary antitumor activity of continuous intravenous infusion of FS-1502 in patients with HER2-expressing advanced malignant solid tumors. and efficacy and safety in patients with HER2-positive locally advanced or metastatic breast cancer.

The phase Ia dose ramping study will enroll patients with advanced, HER2-expressing malignant solid tumors who have failed or are unable to receive standard treatment. FS-1502 was administered as follows and treatment cycle:

- Patients enrolled based on the 1.2 and 2.0 protocols: IV every 4 weeks for 28-day cycles;
- Patients enrolled based on the Version 3.0 and Version 4.0 regimens: intravenous drip every 3 weeks starting at 1.0 mg/kg for 21-day cycles;

**Note:** The dosing frequency will be adjusted while the dose ramp continues, and new patients will be treated every 3 weeks for a 21-day cycle starting from the effective date of the version 3.0 protocol; The safety, efficacy, and PK data for the 3-week dosing regimen will be fully evaluated subsequently, and the dosing frequency will be adjusted to once every 2 weeks if necessary.

Phase Ib included patients with advanced breast cancer with high HER2 expression and failure of anti-HER2 therapy who had received at least 2 prior lines of treatment. Dosage and frequency for Phase Ib will be based on the RP2D obtained in Phase Ia.

FS-1502 Safety observations included monitoring and recording of AEs and SAEs, and performing laboratory tests as specified in the protocol. (including routine blood test, blood biochemistry and urine test, etc.), 12-lead electrocardiogram (ECG), and vital signs. The severity of AEs will be evaluated according to the NCICTCAE version 5.0 criteria.

Based on the efficacy assessment per RECIST 1.1, FS-1502 using imaging methods. (computed tomography [CT] or nuclear magnetic resonance [MRI]) Patients were assessed for tumor. Tumor assessments will be performed every 2 cycles (42 days/56 days ±7 days) until the patient has progressed, died, the investigator's decision, or the patient voluntarily withdraws from the study.

Blood samples were collected in Phase Ia to assess the concentrations and parameters of FS-1502, MMAF, and total antibodies. In Phase Ib, blood will be drawn for Pop PK analysis prior to dosing on the first day of each cycle. Serum samples were collected prior to dosing on the first day of each treatment cycle and tested for anti-drug antibodies (ADA) until the last dose was reached. Samples tested positive for ADA were tested for neutralizing antibodies (NAb).

**3.2 Study Design**

**3.2.1 Dose ramp-up design in phase Ia**

In this study, it is planned to start climbing from the 0.1 mg/kg dose group, and tentatively, 0.1 mg/kg, 0.2 mg/kg, 0.4 mg/kg, 0.6 mg/kg, 0.8 mg/kg, 1.0 mg/kg, 1.3 mg/kg, Patients were enrolled in the traditional 3 + 3 mode, with the following dose ramping principles:

- According to the available non-clinical data, the starting dose is tentatively 0.1mg/kg, and the dose is titrated by 3 + 3. The pre-set 7 dose groups in this study (as shown in the following table), if 1.3mg/kg is safe and tolerated and meets the linear kinetic characteristics, and combined with PK and PD, Safety and efficacy results, combined assessment for continued dose escalation, with a subsequent 33% dose escalation (proportionally adjusted based on previous data results) up to the MTD or RP2D. A total of 21 to 42 evaluable patients are expected to be enrolled. However, the dose for the climbing phase was not limited to these dose groups and the number of enrolled was not limited to 21-42.
- If one of the 3 patients in dose group n had a DLT, add another 3 patients to that dose group. If none of the newly added patients experienced a DLT (1/6), escalate to the next dose. If 2 or more patients have a DLT, enrollment in that dose group should be stopped and escalation to the next dose group should not be allowed;
- DLT events were observed in <33% of patients evaluable for DLT events. (Up to 1 out of 6 patients or 0 out of 3 patients) The highest dose group was considered as the MTD; If the MTD was not observed, the appropriate treatment exposure dose or RP2D was determined based on the antitumor efficacy evaluation, safety data, and PK data.
- If the MTD is reached at the starting dose, a lower dose will be considered.
- The patient was given less than 80% of the planned dose during the DLT observation period due to non-DLT reasons and required case replacement.
- The investigator and the sponsor will review the safety data for each dose group after completing the DLT observation for each dose group, and the efficacy and PK data that have occurred in all patients enrolled.
- When more than 3 eligible patients were screened in a dose group, patients with more than 3 patients were allowed to be expanded and patients with expanded enrollment were not included in the DLT assessment.

The dose ramping study is expected to enroll a total of approximately 21 to 42 evaluable patients (Table 3-1), but is not limited to 21 to 42 patients.

**Table 3-1 Dose Esacalation design**

| Dose group | 1 | 2 | 3 | 4 | 5 | 6 | 7 |
| --- | --- | --- | --- | --- | --- | --- | --- |
| Increase from previous dose (%, tentative) | / | 100 | 100 | 50 | 33 | 25 | 30 |
| Dose Level (mg/kg, tentative) | 0.1 | 0.2 | 0.4 | 0.6 | 0.8 | 1.0 | 1.3 |
| Number of patients (projected) | 3 - 6 | 3 - 6 | 3 - 6 | 3 - 6 | 3 - 6 | 3 - 6 | 3 - 6 |

**3.2.2 Dose Modification Protocol**

Dose adjustments were not allowed during the DLT observation period. Dose interruptions due to non-DLT-related toxicities were allowed during the first cycle.

Dose ramping phase Patients were allowed dose interruptions following the DLT observation period based on the toxicity side effect of FS-1502 until the toxicity resolved to Grade 0-1 or a maximum of 3 dose reductions, each by a dose grade. The lowest dose in Phase Ia was not less than 0.2 mg/kg, and the lowest dose in Phase Ib was not less than 1.0 mg/kg.

After obtaining the recommended dose for clinical Phase II, the investigator may adjust the treatment to the recommended dose for Phase Ia patients who continue treatment in the trial group according to the patient's clinical benefit and after obtaining the patient's full informed consent.

**3.2.3 Definition and Assessment of Dose-Dependent Toxicity**

DLTs are defined as AEs or laboratory abnormalities that occur during the DLT observation period and are judged by the Investigator and/or the Sponsor to be at least possibly related to FS-1502 and graded using NCICTCAE Version 5.0 criteria for toxicity that meet any of the following.‑

**Table 3-2 Dose-dependent toxicity criteria**

| **Toxicity** | **Any of the following is a DLT** |
| --- | --- |
| hematology | Grade 4 hematological toxicity, as follows:   - Grade 4 neutropenia, lasting ≥ 3 days after symptomatic treatment; - Grade 4 neutropenia with fever. (≥ 38.0 degrees Celsius for 1 hour or ≥ 38.3 degrees Celsius); - Grade 4 thrombocytopenia or Grade 3 thrombocytopenia with significant bleeding |
| Non-hematologic | Grade 3 or higher nonhematologic toxicities not due to disease progression   - Any Grade 3 or higher elevated ALT, AST, bilirubin; - Grade 3 nausea, vomiting, diarrhoea, and electrolyte disturbances that persist for more than 3 days after best supportive care); - QTc interval ≥501 ms (average of at least two ECGs) or QTc interval ≥60 ms from baseline; - Grade 2 or higher interstitial pneumonia; - Grade 3 or higher renal toxicity that did not recover within 7 days, including nephrogenic hematuria, proteinuria, decreased creatinine clearance, etc. - Grade 3 or higher amylase elevation or Grade 2 or higher pancreatitis |
| Other: toxicities that, in the opinion of the investigator and the sponsor, require a dose adjustment or suspension of study drug treatment, or clinically unacceptable; | |
| Toxicity events and severity classifications were defined using NCI-CTCAE version 5.0; | |
| DLT=dose-limiting toxicity; Investigator and Sponsor = Data Safety Review Board. | |

The investigator and the sponsor will review the safety data, efficacy data, and PK data for each dose group after the DLT observation is completed for each dose group.

DLT-evaluable patients in this study were required to meet one of the following conditions:

- Use of at least 80% of the planned medication and completion of the DLT observation period without a DLT;
- DLTs occurred during the observation period of continuous dosing DLT.

**3.2.4 Follow-up of Dose-Dependent Toxicity**

After the occurrence of a DLT, the DLT event was recorded, dose interruption or delay was allowed, and once the toxicity recovered to Grade 1 or below, the dose level was reduced by one grade to resume. If a patient is permanently discontinued for ≥ 2 cycles (42/56 days) due to drug-related toxicities, the patient must be followed weekly for 3 weeks and then at maximum intervals of 3 weeks (can be followed by telephone) until recovery or stabilization.

**3.2.5 Ib Dose Expansion Study**

Fifty patients with advanced breast cancer with high HER2 expression and failure of anti-HER2 therapy who had received at least 2 prior lines of treatment were enrolled.

**3.2.6 Tumor Assessment**

Tumor assessments will be performed every 2 cycles (42/56 days ±7 days) according to RECIST v1.1 criteria until disease progression, death, investigator decision, or patient voluntary withdrawal from the study.

**3.3 End of study**

Death occurred 1 year after the start of treatment for the last enrolled patient or 50% of the patients, whichever was earlier. Patients with non-progressive disease at the end of the trial will be determined by the investigator whether to continue FS-1502 or switch to another treatment regimen based on clinical benefit and patient selection.

**4 Selection of study subjects**

**4.1 Inclusion criteria**

1. ≥ 18 years of age, regardless of gender;
2. **Phase Ia Dose Climbing Study:** Patients with HER2-expressing advanced malignant solid tumors who have failed or are unable to receive standard or no standard of care after prior standard of care (including surgery, chemotherapy, radiotherapy, or biologic therapy):

a HER2 overexpression: IHC3+, IHC2+/FISH+, or FISH+

b HER2 low expression: IHC1+, IHC2+ but FISH-

**Phase Ib Dose Expansion Phase:** Histologically or cytologically confirmed HER2-high-expressing patients with advanced breast cancer who have received at least 2 prior lines of treatment and who have failed anti-HER2 therapy, as follows:

a HER2 overexpression of IHC3+, IHC2+/FISH+, or FISH+;

b Patients with advanced breast cancer who have failed prior anti-HER2 therapy and have received at least 2 lines of therapy, with postoperative adjuvant therapy such as disease progression during treatment and within 12 months after the end of treatment as one line of treatment;

c Evidence of disease progression or intolerable toxicity confirmed by the investigator or documented in medical history prior to enrollment;

d Patients can be enrolled by providing a written HER2 test report from the local laboratory, and patients without a HER2 test report must provide sufficient paraffin sections or fresh tumor tissue specimens to the study center or central laboratory for confirmation.

1. Eastern Cooperative Oncology Group (ECOG) performance status score of 0 or 1;
2. Expected survival of at least 12 weeks;
3. Patients had adequate organ and bone marrow function: absolute neutrophils ≥1.0×109/L; hemoglobin ≥ 90 g/L (no red blood cell transfusion within 14 days); platelet ≥ 100 x 109/L; Serum total bilirubin ≤ 1.5 x upper limit normal (ULN) and ≤ 3.0 x ULN in patients with Gilbert's syndrome; Aspartate aminotransferase (AST), alanine aminotransferase (alanine aminotransferase, ALT) ≤ 2.5×ULN; In patients with liver metastases, AST and ALT should be less than or equal to 5×ULN. Creatinine < 1.5 x ULN and creatinine clearance ≥ 60 mL/min (calculated by the Cockroft-Gault equation); Blood potassium ≥3.5 mmol/L; albumin ≥ 3 g/dL; Known left ventricular ejection fraction (LVEF) >50%; Urinary protein ≤ 1 + or 24-hour urinary protein quantitation < 1.0 g;
4. At least one non-cranial measurable lesion as assessed according to RECIST version 1.1;
5. Male or female patients of childbearing potential must agree to use effective methods of contraception, such as double barrier methods, condoms, oral or injectable contraceptives, intrauterine devices, etc., during the study and within 30 days of the last dose of study medication;
6. Be able to understand and voluntarily sign a written informed consent form.

**4.2 Exclusion criteria**

1. Chemotherapy, targeted therapy, radiotherapy, etc. within 14 days of administration or 5 half-life, whichever is shorter; Patients who have received major surgery, tumor immunotherapy, and monoclonal antibody antineoplastic therapy within 4 weeks before starting administration;
2. Patients who have participated in other clinical trials within 4 weeks or 5 drug half-lives, whichever is shorter, prior to the start of dosing; Patients who have received prior treatment with similar drugs;
3. Patients previously treated with other anti-HER2 ADC drugs other than T-DM1;
4. Uncontrolled stable central nervous system metastases or lesions were allowed to enroll patients who met the following conditions:
5. Having received local treatment and stable disease for more than 2 months;
6. No corticosteroid or other dehydration therapy is required and the dose of antiepileptic drugs is stable (if applicable);
7. No meningeal metastases;
8. Patients with uncontrolled and stable diabetes. (Patients on stable insulin regimens or antidiabetic regimens, specialist assessed stable glycaemic control, were allowed to enroll)
9. The toxicities of prior antineoplastic therapy have not recovered (> NCI-CITCAE 5.0 Grade 2); Neurotoxicity in patients previously treated with chemotherapy requiring a return to NCI-CTCAE 5.0 Grade 2 or less;
10. with corneal epitheliopathy (except mild punctate keratopathy);
11. Patients take drugs that prolong the QTc interval. (primarily class Ia, Ic, III antiarrhythmic drugs) or the presence of risk factors for prolonged QTc intervals, such as uncorrectable hypokalaemia, hereditary long QT syndrome; Medications with potential QTc prolongation are listed in Attachment 6;
12. Cardiac function and disease are one of the following:
13. Three 12-lead electrocardiogram (ECG) measurements were performed at the site during the screening period, and the mean value was calculated by the instrument's QTc formula, QTc > 470 ms;
14. New York Heart Association (NYHA) grade ≥ 3 congestive heart failure;
15. Clinically significant arrhythmias, including but not limited to complete abnormal left bundle branch communication, second degree AV block.
16. Pregnant or lactating women;
17. Known hypersensitivity to any of the excipients of FS-1502;
18. Clinically significant active bacterial, fungal, or viral infections, including hepatitis B (HBV surface antigen positive and HBV DNA over 1000 IU/ml) or Hepatitis C (HCV RNA positive), Human Immunodeficiency Virus Infection (HIV positive);
19. Any other disease or condition that, in the opinion of the investigator, may affect compliance with the protocol or clinically significant for the patient to sign the ICF. (e.g., active or uncontrollable infection, etc.).

**4.3 Treatment Discontinuation Criteria**

Patients have the right to withdraw from the study at any stage of the study. Patients must discontinue study drug if they experience any of the following reasons:

1. disease progression;
2. death;
3. Intolerable toxicities related to study treatment;
4. The patient is pregnant;
5. The investigator determines that the study should be terminated in the best interests of the patient;
6. the patient or his/her legal representative requests withdrawal from the study;
7. The patient was lost to follow-up for more than 3 months;
8. Another systemic antineoplastic treatment was used;
9. The sponsor notified the closure of the clinical study.

**4.3.1 Treatment of patients discontinuing treatment**

The reason for the patient's premature discontinuation of treatment should be recorded in the original medical record and the eCRF, and the subsequent treatment after the patient's discontinuation should be recorded in the original medical record. If the patient discontinues treatment prematurely due to an AE/clinically significant abnormal laboratory test result, the investigator must follow the patient until the AE/abnormal value normalizes, or until the patient deems that further follow-up is not necessary.

Subsequent antineoplastic therapy and study-related SAEs will be collected every three months starting from treatment discontinuation.

**4.3.2 Discontinuation Patient Replacement**

A case replacement was required for the patient who received less than 80% of the planned dose prior to the DLT assessment visit due to non-DLT reasons. Substitute patient number was extended.

**5 Study Flow**

**5.1 Phase Ia**

1. **Screening/Baseline Period (-28 to - 1 day)**

All patients must provide a written ICF prior to any specific study assessment and procedure.

A complete medical history will be recorded at screening:

- Demographic data: including date of birth, sex, race, ethnicity, and birth status.
- Past medical history. (Collection of all past medical history, except for this indication, beginning before the signing of the ICF, and considered relevant to the study).
- Prior tumor history: including date of tumor diagnosis, histopathological classification and grade, ER status (including ER positive rate), PR status (including PR positive rate), HER2 status, Ki-67 proliferation index, clinical stage, presence of metastasis and site of metastasis, Whether only bone lesions were present; Performance status; Time from diagnosis to relapse, indication of chemotherapy, start/end date of previous treatment regimen, availability of targeted therapy and type of treatment (adjuvant therapy, neoadjuvant therapy, advanced therapy), number of lines of advanced therapy and treatment regimen, best treatment assessment, and disease progression date; The history of radiotherapy should include start/end date, site of radiotherapy. Previous clinically significant procedures. (e.g., invasive procedures for diagnosis or treatment such as bronchoscopy, biopsy, etc.) To be recorded on the eCRF, including start and end dates, operation name and site.
- Tumor tissue sections or fresh tissues were obtained for IHC staining and/or FISH gene amplification testing. (Previous HER2 status report can be used as the basis for enrollment, and tissue specimens without HER2 status report prior to enrollment must be sent to the site or central laboratory for confirmation).
- Physical examination: include height, weight, head, eyes, ears, nose, throat, neck, heart, chest (including lungs), abdomen, extremities, skin, lymph nodes, nervous system, and general patient condition.
- Vital signs.
- ECOG score.
- Laboratory tests: The laboratory tests for C1D1 should be performed within 7 days before the first administration. Specific laboratory test indicators include routine blood test, blood biochemistry test and routine urine test.
- Routine blood test: red blood cell count, hemoglobin, hematocrit, white blood cell count and differential. (neutrophils, lymphocytes, eosinophils, monocytes, basophils and other cells) and platelet count, etc.
- Blood chemistry tests included total protein, albumin, blood glucose, total cholesterol, low density lipoprotein, high density lipoprotein, triglyceride, urea, creatinine, alkaline phosphatase, lactate dehydrogenase, total bilirubin, direct bilirubin, indirect bilirubin, AST, ALT, Calcium, phosphorus, magnesium, potassium, sodium, chlorine, serum amylase, etc.
- Urine routine includes specific gravity, PH, urine sugar, protein, urine white blood cells, urine red blood cells and ketone bodies. If positive urine protein is grade 2 or more, a 24-hour quantitative urine protein test is required.
- Cardiac ultrasound examination
- Cardiac function NYHA classification.
- 12-lead ECG (three consecutive ECGs, each approximately 5 minutes apart).
- HBV (add HBV-DNA if surface antigen is positive), HCV, and HIV testing.
- Coagulation tests: including prothrombin time (PT), activated partial thromboplastin time (aPTT), international normalized ratio (INR), and thrombin time (TT).
- Blood HCG test, if applicable.
- Tumor Assessment: Tumors were evaluated according to RECIST 1.1. CT or MRI scans of the chest, abdomen, pelvis, and head should be performed at Screening. Where clinically indicated, any other known or suspected disease site may be examined by appropriate methods, such as bone scan or neck CT scan. Target lesion records: number of lesions, location, description, measurement of maximum diameter per lesion and measurement of minimum diameter of lymph node, sum of diameters including all target lesions.
- Concomitant medications/concomitant treatments: from 28 days prior to the start of the first medication until 30 days after the last dose or until the start of other antineoplastic therapy, whichever occurs first.
- Adverse events: Collected from the time the informed consent is signed until 30 days after the end of treatment or until the start of other antineoplastic therapy, whichever occurs first.

1. **Phase Ia Dosing Period**

- A physical examination.
- Vital signs.
- ECOG score.
- Tumor Assessment: The same imaging approach should be used for baseline and subsequent assessments. Tumor assessment must be completed prior to the next cycle. Tumor assessments were calculated by C1D1, every 2 cycles (42 days/56 days ±7 days), regardless of the impact of discontinuation. An unscheduled tumor assessment should be performed in patients with suspected disease progression prior to the start of the next scheduled tumor assessment.
- Laboratory tests: including routine blood, urine, and blood chemistry tests, should be performed within 7 days before the first administration day. (If the relevant laboratory tests for the screening period are still within the time window, the values for the screening period can be used.)
- 12-lead ECG, repeated three times, approximately 5 minutes apart, before the first dose. If the test results within 7 days prior to Day 1 are available, it is not necessary to repeat the test before the first dose on Day 1.
- Cardiac ultrasound.
- Cardiac function NYHA classification.
- Coagulation test. (On Cycle 1 Day 1 if the screening test results are clinically significant. Follow-up tests as clinically needed).
- Concomitant medication/concomitant therapy.
- Evaluation of AEs and SAEs.
- DLT assessment (cycle 1 only).
- Blood sampling for PK and immunogenicity (see Attachment 1).
- Patient Dosing: The study drug should be administered after the completion of an assessment, including laboratory tests, confirming the safety of the patient to continue administering the trial drug.

1. **End of treatment**

- A physical examination.
- Vital signs.
- ECOG score.
- Laboratory tests, including routine blood tests, routine urine tests, and blood chemistry.
- Coagulation tests.
- Blood HCG test, if applicable.
- 12-lead ECG.
- Cardiac ultrasound examination
- Cardiac function NYHA classification.
- Tumor assessment.
- Concomitant medication/concomitant treatment.
- Adverse events.

1. **30-day follow-up after the end of treatment**

- A full-body physical examination.
- Vital signs and ECOG scores.
- Laboratory tests, including routine blood tests, routine urine tests, and blood chemistry.
- 12-lead ECG.
- Coagulation test: as clinically required.
- Concomitant medication/concomitant therapy.
- Adverse events.
- Cardiac ultrasound examination
- Cardiac function NYHA classification

1. **Survival follow-up**

Subsequent antineoplastic treatment information and survival were collected every 3 months (± 7 days) starting from the safety visit or the end-of-treatment visit, whichever occurred later, until death, loss to follow-up, withdrawal of consent, or trial termination.

**5.2 Phase Ib**

1. **Screening/Baseline Period (-28 to - 1 day)**

All patients must provide a written ICF prior to any specific study assessment and procedure.

A complete medical history will be recorded at screening:

- Demographic data: including date of birth, sex, race, ethnicity, and birth status.
- Past medical history. (Collection of all past medical history, except for this indication, beginning before the signing of the ICF, and considered relevant to the study).
- Prior tumor history: including date of tumor diagnosis, histopathological classification and grade, ER status (including ER positive rate), PR status (including PR positive rate), HER2 status, Ki-67 proliferation index, clinical stage, presence of metastasis and site of metastasis, Whether only bone lesions were present; Performance status; Time from diagnosis to relapse, indication of chemotherapy, start/end date of prior treatment regimen, availability of targeted therapy and type of treatment (adjuvant therapy, neoadjuvant therapy, advanced therapy), number of lines of advanced therapy and treatment regimen, best treatment assessment, and disease progression date; The history of radiotherapy should include start/end date, site of radiotherapy. Previous clinically significant procedures. (e.g., invasive procedures for diagnosis or treatment such as bronchoscopy, biopsy, etc.) To be recorded on the eCRF, including start and end dates, operation name and site.
- Obtain tumor tissue sections or fresh tissues for ICH staining and/or FISH gene amplification testing. (Previous HER2 status report can be used as the basis for enrollment, tissue specimens without HER2 status report prior to enrollment must be sent to the site or central laboratory for confirmation).
- Physical examination: include height, weight, head, eyes, ears, nose, throat, neck, heart, chest (including lungs), abdomen, extremities, skin, lymph nodes, nervous system, and general patient condition.
- Vital signs.
- ECOG score.
- Laboratory tests: should be performed within 7 days before the first dose; Specific laboratory test indicators include routine blood test, blood biochemistry test and routine urine test.
- Routine blood test: red blood cell count, hemoglobin, hematocrit, white blood cell count and differential. (neutrophils, lymphocytes, eosinophils, monocytes, basophils and other cells) and platelet count, etc.
- Blood chemistry tests included total protein, albumin, blood glucose, total cholesterol, low density lipoprotein, high density lipoprotein, triglyceride, urea, creatinine, alkaline phosphatase, lactate dehydrogenase, total bilirubin, direct bilirubin, indirect bilirubin, AST, ALT, Calcium, phosphorus, magnesium, potassium, sodium, chlorine, serum amylase, etc.
- Urine routine includes specific gravity, PH, urine sugar, protein, urine white blood cells, urine red blood cells and ketone bodies. If protein 1 + is present during screening, a 24-hour quantitative urine protein test is required.
- Heart ultrasound.
- Cardiac function NYHA classification.
- 12-lead ECG (three consecutive ECGs, each approximately 5 minutes apart).
- HBV, HCV, and HIV testing.
- Coagulation tests.
- Ophthalmological examination: visual acuity and slit lamp examination.
- Blood HCG test, if applicable.
- Tumor Assessment: Tumors were evaluated according to RECIST 1.1. Contrast-enhanced CT or MRI scans of the chest, abdomen, pelvis, and head should be performed at Screening. If clinically indicated, any other known or suspected disease site may be examined by appropriate methods, such as a bone scan or a CT scan of the neck. Target Lesion Recording: number of lesions, location, description, measurement of maximum diameter per lesion and measurement of minimum diameter of lymph node, sum of diameters including all target lesions.
- Concomitant medications/concomitant treatments: from 28 days prior to the start of the first medication until 30 days after the last dose or until the start of other antineoplastic therapy, whichever occurs first.
- Adverse events: Collected from the time the informed consent is signed until 30 days after the end of treatment or until the start of other antineoplastic therapy, whichever occurs first.

1. **Ib dosing phase**

- A physical examination.
- Vital signs.
- ECOG score.
- Laboratory tests (if results available within 7 days prior to Day 1, it is not necessary to repeat the test before the first dose on Day 1;).
- 12-lead ECG. (Repeated three times, approximately 5 minutes apart, during the screening period and before the first dose). If the test results within 7 days prior to Day 1 are available, it is not necessary to repeat the test before the first dose on Day 1.
- Cardiac ultrasound (as clinically required).
- Cardiac function NYHA classification (as clinically required).
- Coagulation test. (on Day C1D1 if clinically significant at Screening; cycle 2 and beyond, as clinically needed).
- Ophthalmic examination: visual acuity and slit lamp examination, results obtained within 3 days prior to dosing in each cycle.
- Tumor Assessment: The same imaging approach should be used for baseline and subsequent assessments. Tumor assessment must be completed prior to the next cycle. Tumor assessments should be calculated as C1D1, every 2 cycles (42 days/56 days ±7 days), regardless of the impact of drug discontinuation. An unscheduled tumor assessment should be performed in patients with suspected disease progression prior to the start of the next scheduled tumor assessment.
- Concomitant medication/concomitant treatment.
- Evaluation of AEs and SAEs.
- PK blood sampling.
- Dosing to the patient: The study drug should be administered after the completion of an assessment, including laboratory tests, confirming that the patient is safe to continue administering the trial drug.

1. **End of treatment**

- A physical examination.
- Vital signs.
- ECOG score.
- Laboratory tests.
- 12-lead ECG.
- Cardiac ultrasound: as clinically required.
- Cardiac function NYHA classification: according to clinical need.
- Coagulation tests.
- Ophthalmological examination: visual acuity and slit lamp examination.
- Blood HCG test, if applicable.
- Tumor assessment.
- Concomitant medication/concomitant treatment.
- Adverse events.

1. **30-day follow-up after the end of treatment**

- A full-body physical examination.
- Vital signs and ECOG scores.
- Laboratory tests.
- 12-lead ECG.
- Coagulation test: as clinically required.
- Ophthalmological examination: visual acuity and slit lamp examination.
- Concomitant medication/concomitant treatment.
- Adverse events.
- Cardiac ultrasound: as clinically required.
- Cardiac function NYHA classification: as clinically required.

1. **Survival follow-up**

Subsequent antineoplastic treatment information and survival were collected every 3 months (± 7 days) starting from the safety visit or the end-of-treatment visit, whichever occurred later, until death, loss to follow-up, withdrawal of consent, or trial termination.

**6 Study Drug and Method**

**6.1 Study Drug**

FS-1502 was provided by the Sponsor in clinical dosage forms of FS-1502 frozen solution (60 mg/12 ml strength) or FS-1502 lyophilized powder (30 mg/bottle strength).

FS-1502 frozen solution: After thawing according to the drug manual, draw different volumes of FS-1502 according to the dosage, add 100 ml of 0.9% normal saline into intravenous drip;

FS-1502 lyophilized powder: suck 6mL sterilized water for injection with a 10mL syringe, puncture the rubber stopper, and reconstitute FS-1502 upside down until the sample is completely dissolved. The concentration after reconstitution is 5mg/mL. The FS-1502 solution was injected into 100 mL of 0.9% normal saline intravenously prior to administration.

Please refer to the Drug Brochure and the Investigator's Brochure for specific medication information and handling instructions.

**6.2 Packaging and Labeling of Study Drug**

Drug labels will be designed in accordance with national drug labeling requirements and will include information such as the package, name, strength, storage conditions and date of manufacture of the drug, but will not contain patient privacy information.

**6.3 Storage and Transportation of Study Drug**

The sponsor will provide the study drug according to the site's anticipated enrollment schedule. The study drug will be shipped to the study site via a third-party logistics company with the shipping qualification. Authorized site staff faxed the signed delivery note to the sponsor to confirm receipt of the drug.

The study drug may only be used in the study and may only be administered by a person authorized by the investigator. All empty bottles, returned medications, etc., will be recorded in detail throughout the study. The study drugs will be recovered and destroyed in accordance with the relevant regulations of China.

**6.4 Medication management**

The study drug should be kept in a safe and managed area. The frozen solution should be kept sealed at -40 ±5°C and the lyophilized powder should be kept sealed at 2-8°C. For details about the storage conditions, refer to the drug manual. The investigator/pharmacist is responsible for the preservation of the drug and the temperature monitoring.

**6.5 Medication Dispensation**

FS-1502 used in all studies was prescribed by the investigator and any dose changes must be recorded in the eCRF.

**6.6 Dosage and Period**

Phase Ia: patients enrolled based on the 1.2 and 2.0 protocols: intravenous drip every 4 weeks for 28-day cycles; Newly enrolled patients in this regimen: intravenous drip every 3 weeks starting at 1.0 mg/kg for a 21-day cycle;

Phase Ib: 2.3 mg/kg intravenously every 3 weeks for 21-day cycles.

Dosing will continue until the patient experiences loss of clinical benefit, intolerable toxicity, death, withdrawal of ICF, or other discontinuation criteria are met.

**6.7 Method of medication**

Patients must be given the following medications:

1. Try to give the drug at the same time every cycle, and the patient must come to the hospital under the guidance of the researcher;
2. Dose calculated based on patient body weight as specified in the protocol:

FS-1502 frozen solution: Extract different volumes of dissolved FS-1502, with the drug volume accurate to 0.1ml, with the second decimal point rounded off (e.g., 0.96ml, 0.9ml), add 100 ml into 0.9% NaCl, intravenous drip, Instillation is completed within 60-90 minutes.

FS-1502 lyophilized powder: Take one piece of FS-1502 lyophilized powder, extract 6mL sterilized water for injection with a 10mL syringe and dissolve it into FS-1502 solution with a concentration of 5mg/mL, slowly rotate until the powder is not completely dissolved. The appropriate FS-1502 solution was drawn from the dose calculated based on the patient's body weight and added to 100 mL of 0.9% sodium chloride injection for intravenous drip, and the infusion was completed within 60-90 minutes.

1. If the patient's body weight changes by more than 10% from baseline, the dose will need to be recalculated.
2. If infusion-related reactions (IRRs) occur, suspend the IV infusion or reduce the IV infusion rate for no more than 30 minutes, and provide antihistamine or corticosteroid therapy as clinically indicated. Any infusion-related adjustments that occur during the IV drip will be recorded in the eCRF.
3. All medications prescribed and used and dose adjustments must be recorded in the designated medication record file and entered into the eCRF.

**6.8 Concomitant medication/treatment**

All medications or concomitant treatments during the study, prophylactic medications, and reasons for medications must be recorded on the Concomitant Medication Form. Pre-enrollment antineoplastic therapy, including medication, radiotherapy, and surgery, will be recorded separately in the medical history.

Medications or treatments required for supportive care (e.g., antiemetics, antidiarrheal, etc.) are generally allowed and recorded in the eCRF.

Any medication and non-pharmaceutical therapy other than study drug (including physiotherapy or blood transfusion) during the study must be recorded on the Concomitant Medication Form.

**6.9 Permitted and prohibited medications and non-pharmacological therapies**

**6.9.1 Permitted medication and non-pharmacological therapies**

Patients should receive adequate supportive care during the study, including transfusions of blood and blood products, antibiotic therapy, antiemetics, antidiarrheal, analgesics, and other appropriate treatments in accordance with the patient's institution guidelines. Patients may receive palliative radiotherapy for bone metastases only.

**6.9.2 Prohibited medications and non-pharmacological therapies**

Patients should not receive other anticancer therapy during the treatment period in this study. Patients should not receive chemotherapy, targeted therapy, radiotherapy, etc. within 14 days or 5 half-lives before the first dose; Do not receive tumor immunotherapy, monoclonal antibody antineoplastic therapy within 4 weeks prior to the start of administration.

Medications with the potential to prolong the QTc interval should be avoided during the study unless there is no alternative treatment. If such a drug must be used, written approval from the investigator and/or the sponsor is required. Medications with the potential to prolong the QTc interval are listed in Attachment 6.

**6.10 Overdose**

This study is the first human study of FS-1502 and therefore there are no data on FS-1502 overdose, so the scope of specific overdose is not defined at this stage. At present, there are no known detoxification treatments for this overdose. It is recommended that the investigator closely observe the patient who has received more than the planned dose for the necessary supportive care and follow-up.

Upon notification of an event exceeding the planned dose, the sponsor and the contract research organization personnel must be notified within 24 hours of notification.

**6.11 Treatment Compliance**

In this study, dosing was administered by intravenous infusion according to the dose group in which the patient was enrolled. FS-1502 will be administered in an environment with emergency medical facilities and personnel trained in the monitoring and management of emergency conditions.

The FS-1502 will be administered in a 100 mL 0.9% NaCl IV infusion bag and IV infusion tube, with a product contact surface material of polypropylene, over 60-90 minutes. The product contact surface material for IV tubes is PVC or Polyethylene and 0.2 micron inline filter with polyethersulfone membrane. The investigator shall promptly and accurately record the planned dosage, date, and actual dosage of study medication administered to each patient.

Dose modifications are not allowed during the DLT observation period, and at the end of the DLT observation and the dose extension phase, for dose modifications, interruptions, or terminations, refer to Section 6.12 of the Adverse Event Handling Guidelines.

For detailed instructions on the preparation, storage, and administration of the drug, refer to the drug instructions and the Operator's Manual.

At the end of the study, all remaining items and medications must be returned to the sponsor.

Non-compliance was defined as a patient receiving less than 80% or more than 120% of the planned medication during the study treatment period.

**6.12 Principles for Handling Adverse Reactions during the Study**

If a patient experiences an NCI CTCAE v5.0 Grade 3 or higher and/or unacceptable toxic event, the study drug will be discontinued and supportive care will be given according to local routine. If the toxicity recovered to an NCI CTCAE grade 1 or less within 2 cycles (42/56 days) and the patient did not progress, the original dose of the study drug could be continued or the dose could be reduced after discussion between the investigator and the sponsor. If the toxicity recovers to ≤ NCI CTCAE Grade 1 after 2 cycles (42/56 days), the investigator will discuss with the sponsor's medical monitoring, and the decision to continue the medication will be made based on the patient's clinical benefit. If the patient recovered within 2 cycles (42/56 days) and did not progress at the time of recurrence of a Grade 3 toxic event, the study drug could be re-administered by one dose reduction. At the third occurrence of a Grade 3 toxicity event, if the patient recovered within 2 cycles (42/56 days) and did not progress, then the dose was reduced by one additional dose and re-administered. Patients were allowed a maximum of 3 dose modifications after which no further dose modifications were allowed, but a dose hold was allowed. Dose downregulation was not allowed during the DLT observation period during the dose ramp phase. Refer to Table 6-1 for the guidelines for dose reduction.

**Table 6-1 FS-1502 Dose Reduction Principles**

| **Start Dose** | **Dose Grade 1** | **Dose Grade 2** | **Dose Grade 3** |
| --- | --- | --- | --- |
| 2.3 mg/kg | 1.7 mg/kg | 1.3 mg/kg | 1.0 mg/kg |

If the patient has any toxic side effect during the study, the treatment and dose suspension and dose reduction of the toxic side effect should follow the following principles.

**6.12.1 Hematological Toxicity**

Once onset, permanent discontinuation was made with reference to the definition of DLT or per the dose titration protocol.

**Diarrhea**

If the patient has grade 1-2 diarrhea, close observation or medication to improve intestinal function can be used; If grade 3 diarrhea occurs, use symptomatic support to improve intestinal function drugs, generally within 3 days recovery, can continue to use; Diarrhoea was treated for more than 3 days and the study drug was discontinued until resolution to CTCAE Grade 1 or less, and if the patient recovered within 2 cycles (42/56 days) and did not progress, the study drug could be re-administered at a reduced dose. If diarrhoea of Grade 3 or higher recurs for more than 3 days, discontinue and give active treatment, and once diarrhoea has resolved to less than or equal to CTCAE Grade 1, the study drug may be re-administered by one dose reduction. Patients were allowed a maximum of 2 dose modifications.

**6.12.2 QTc Prolongation**

Grade 4 QTc prolongation occurred. (defined as QTc ≥ 501 ms or > 60 ms increase from baseline and dersades ventricular tachycardia, polymorphic ventricular tachycardia, or severe asymptomatic arrhythmia) FS-1502 treatment must be discontinued. Serum potassium and magnesium levels can be monitored and corrected to normal levels, if required. In the event of QTc ≥500 ms, an electrocardiogram (ECG) should be performed again within 1 hour to confirm QTc interval ≥500 ms and to assess the use of concomitant medications. Once Grade 4 QTc prolongation is confirmed, FS-1502 administration will be permanently discontinued and the patient will be hospitalized for close cardiac monitoring until the QT interval normalizes or cardiology consultation and adequate treatment is initiated. In other cases, the dose modification protocol was followed.

If the patient's QTc interval returns to normal, the dosage may be reduced by another dose level at the discretion of the investigator and the cardiologist.

**6.12.3 Decreased Left Ventricular Ejection Fraction**

Left ventricular ejection fraction (LVEF) was assessed by echocardiography. It is recommended that the same technique be used for the same patient throughout the study. Once symptoms of heart failure or radiographic changes from baseline occur, the following guidelines should be followed:

| No symptoms of heart failure and LVEF decreased to the lower limit of the normal range but ≥ 50%  or ≤ 15% reduction in absolute value compared with baseline | Continue with same dose and repeat LVEF once in a maximum of 4 weeks |
| --- | --- |
| With symptoms of heart failure  40% < LVEF < 50%  or > 15% absolute decrease from baseline | Dosing was withheld until recovery (LVEF ≥ 50% and no symptoms of heart failure). If recovered within 3 weeks, titrate to the next studied dose, otherwise permanently discontinue |
| There was no recovery or  LVEF < 40% | Permanently discontinued and patient was followed up |

**6.12.4 Acute hypertension**

Monitor for high blood pressure and treat it appropriately. In the event of an increase in blood pressure, follow the dose titration protocol.

If an adverse event (e.g. proteinuria) related to hypertension occurs, the opinion of a cardiologist and nephrologist should be sought. Whether recording medical history or adverse events, the NCI CTCAE 5.0 classification is used to determine hypertension, including blood pressure measurements and number of medications to be treated.

**Table 6-2 Hypertension classification (NCI-CTCAE [Version 5.0])**

| **Level 1** | Prehypertension (systolic blood pressure between 120 and 139 mmHg and diastolic blood pressure between 80 and 89 mmHg) |
| --- | --- |
| **Level 2** | Stage 1 hypertension (systolic blood pressure 140-159 mmHg, diastolic blood pressure 90-99 mmHg); Requires medical intervention; Repetitive or persistent (≥24 hours), symptomatic systolic blood pressure increase > 20 mmHg or prior normal range increase > 140/90 mmHg; Requires monotherapy |
| **Level 3** | Stage II hypertension (systolic blood pressure ≥ 160 mmHg, diastolic blood pressure ≥ 100 mmHg); Requires medical intervention; Requires more than one drug treatment or adds to previous treatment |
| **Level 4** | Life-threatening (e.g., malignant hypertension, transient or persistent nerve damage, hypertensive crisis); Needs urgent medical treatment |
| **Level 5** | To die |

**6.12.5 Other Cardiac Events**

ECG, echocardiography must be performed once cardiac adverse events (e.g., ischemic heart disease) are suspected; Troponin, NT-proBNP, and potassium may also be considered.

**6.12.6 Proteinuria**

In the event of a urinary protein ≥ 2 +, dosing was suspended and a 24-hour urinary protein quantification was performed simultaneously. If the 24-hour urine protein quantitation is less than or equal to Grade 1, resume medication; If the 24-hour urinary protein quantitation is ≥ Grade 2, continue withholding and aggressive treatment. Once proteinuria has resolved to grade 1 or less, reduce treatment by one dose and continue to monitor proteinuria. Recurrence of Grade 2 proteinuria occurred again, and dosing was suspended and 24-hour urinary protein quantification was performed simultaneously. When proteinuria has recovered to grade 1 or less, continue treatment at a reduced dose and continue monitoring for proteinuria.

**6.12.7 Other Renal Impairment Events**

In the event of the occurrence, permanent discontinuation of the drug was performed according to the DLT definition or according to the dose adjustment protocol. Urine routine, renal function, electrolytes were checked, and active symptomatic treatment was obtained from the nephrology department.

**6.12.8 Elevated AST/ALT**

In the event of a Grade 3 ALT/AST increase, dosing was withheld until resolution to Grade ≤ 1 or baseline. If recovered within 7 days, resume treatment at the same dose level. If recovery occurs between 7 days and 2 cycle times, treatment is continued by lowering to the next studied dose level after recovery. If there is no recovery within 2 cycles (42/56 days), the treatment is discontinued permanently. If Grade 3 ALT/AST elevation recurs, hold until resolution to Grade ≤ 1 or Baseline (If not recovered within 7 days, discontinue the drug permanently). In the event of > Grade 3 ALT/AST elevations, assessment is required:

- Detailed medical history: such as alcohol, drugs, nutrition, family history, sexual history, travel history, exposure to jaundice patients, surgery, blood transfusion, history of liver disease or allergic diseases and work environment.

- Physical examination and consideration of the possibility of tumor liver metastases.

- Liver imaging (e.g., biliary tract).

- Laboratory tests: AST and ALT, alkaline phosphatase, GGT, albumin, total bilirubin, direct bilirubin, prothrombin time, factor V, CPK, lactate dehydrogenase, and troponin. Testing for hepatitis A, hepatitis B, hepatitis C, and hepatitis E, CMV, EBV, blood glucose, and triglycerides is also recommended.

In the event of ALT >3 ULN accompanied by total bilirubin >2 ULN, no hemolysis, alkaline phosphatase <2 ULN, or no test: permanent discontinuation. In the event of other abnormal laboratory values (including blood glucose and triglycerides) as judged by the investigator to be clinically significant, dosing was withheld until resolution to Grade ≤ 1 or baseline. If complete recovery or grade 1 is achieved within 7 days, resume treatment at the same dose.

**6.12.9 Infusion Reactions**

Monitor infusion reactions, including rashes, vital signs, etc., provide appropriate treatment, and record the outcome of treatment drugs and infusion reactions. The NCI CTCAE 5.0 classification is strictly used to determine infusion reactions, regardless of the medical history or adverse event recording. The following table lists the processing principles.

| Level 1 | Decrease the infusion rate to half, and observe for 30 minutes after the infusion reaction is resolved. If no abnormality is found, consider resuming the original infusion rate |
| --- | --- |
| Level 2 | Suspend infusion, give antihistamine, antipyretic, glucocorticoid, tracheal dilation, oxygen inhalation and other symptomatic treatment, wait for infusion reaction to relieve half-speed drip, subsequent infusion process give antihistamine pretreatment |
| Level 3 or 4 | Stop infusion, active antihistamine, antipyretic, glucocorticoid, tracheal dilation, oxygen, symptomatic treatment, permanent stop trial treatment and contact the medical monitor |

**6.12.10 Pancreatitis**

Monitor amylase and treat as appropriate. In the event of an increase in blood amylase, the dose was adjusted according to the dosage regimen.

Adverse events related to pancreatitis require the advice of a digestive specialist. Regularly detect enzyme changes, detect clinical symptoms and signs, and actively give symptomatic treatment. Whether recording medical history or adverse events, pancreatitis should be judged strictly according to the NCI CTCAE 5.0 classification, and treatment medications and disease outcome should be recorded. The following table lists the processing principles.

| Grade 1 amylase increased | Continue trial medication, monitor amylase, lipase |
| --- | --- |
| Grade 2 increased amylase | Continue study medication, weekly amylase, lipase monitoring, dose reduction may be considered |
| Grade 3 increased amylase or Grade 2 pancreatitis | Discontinue the trial drug, consult the digestive department, give active symptomatic management, if recovered to grade 1 or baseline level within 2 cycles (42/56 days), reduce the dose to the next dose level, if recurrence, stop permanently |
| Grade 4 increased amylase or Grade 3 pancreatitis | Permanently stop the study drug, consult the digestive department, and give active symptomatic treatment, |

**6.12.11 Ocular Toxicity**

Visual acuity and slit lamp results were closely monitored during medication, and in the event of ocular toxicity, the dosage adjustment principles described in the table below were followed. For early prophylaxis, use preservative-free artificial tear drop eyes at the start of treatment, throughout the course of treatment, and 30 days after discontinuation.

Ocular toxicity grade was determined according to the NCI CTCAE 5.0 grading, and treatment drugs and outcomes were recorded.

| **NCI-CTCAE V5.0** | **Dose modification plan** |
| --- | --- |
| **ocular toxicity** | |
| **Dry eye disease** | |
| Level 1 | - Maintain original dose and continue treatment with close observation - Preservative-free artificial tears are recommended, and the frequency can be increased appropriately. |
| Level 2 | - Withhold until grade 1 recovery, use original dose - Use preservative-free artificial tears, calf serum deproteinized eye drops or recombinant bovine basic fibroblast growth factor eye drops |
| Level 3 | - Dose delayed until ≤ Grade 1, reduce by 1 dose - Preservative-free artificial tears, calf serum deproteinized eye drops or recombinant bovine basic fibroblast growth factor eye drops, ophthalmology specialist |
| Level 4 | - Dose was delayed until ≤ Grade 1, and after comprehensive benefit/risk assessment, the dosage was reduced and continued - Preservative-free artificial tears, calf serum deproteinized eye drops or recombinant bovine basic fibroblast growth factor eye drops, ophthalmology specialist |
| keratitis | |
| superficial punctate keratitis | - Maintain original dose and continue treatment with close observation |
| confluent superficial keratitis | - Dose delayed until ≤ Grade 1, reduce by 1 dose - Discontinue treatment if recurrence |
| Ulcerative keratitis or perforation | - Discontinuation of treatment |
| Conjunctivitis or other ocular adverse events | |
| Level 1 | - Continue treatment with original dose and observe closely |
| Level 2 | - Dose was delayed until Grade ≤ 1 after first occurrence, and original dose was resumed - Dose delayed until ≤ Grade 1 after reoccurrence, reduce by 1 dose - Discontinue treatment at third occurrence |
| Grade 3-4 | - Discontinuation of treatment |

**6.12.12 Hypokalemia**

Monitor blood potassium and treat appropriately. In the event of hypokalaemia, dose modification was performed according to the protocol.

In the event of an adverse event related to hypokalaemia, other possible causes of hypokalaemia, such as poor diet, use of diuretics, etc., should be excluded. Regularly monitor the changes of blood potassium, monitor clinical symptoms and signs, and actively give symptomatic treatment. Hypokalemia was judged according to the NCI CTCAE 5.0 grading, and treatment medication and outcome were recorded. The following table lists the processing principles.

| **NCI-CTCAE V5.0** | **Dose modification plan** |
| --- | --- |
| History of previous hypokalemia or hypokalemia found during treatment | - Weekly (or more frequent) blood potassium testing is recommended |
| Level 1 - Level 2 | - oral potassium supplementation - Maintain serum potassium levels between 3.5 and 5 mmol/L (>4 mmol/L is recommended) |
| Level 3 | - Discontinuation of FS-1502 - Intravenous potassium supplementation to maintain a serum potassium level of 3.5-5 mmol/L (>4 mmol/L is recommended) - Cardiac monitoring - Original Dose After Return to Normal - Grade 3 hypokalemia reoccurs, reduce 1 dose once normal |
| Level 4 | - Discontinuation of FS-1502 - Intravenous potassium supplementation to maintain a serum potassium level of 3.5-5 mmol/L (>4 mmol/L is recommended) - Cardiac monitoring - Reduce 1 dose after returning to normal - Discontinuation of treatment for recurrent Grade 4 hypokalemia |

**7 Study Assessment**

**7.1 Safety Assessment**

Mainly include monitoring and recording of AEs and SAEs, performing laboratory tests as specified in the protocol. (including routine blood test, blood chemistry and urine test), 12-lead ECG, and vital signs. The severity of AEs will be evaluated according to the criteria of the NCI-CTCAE (Version 5.0).

**7.1.1 Adverse Events**

**7.1.1.1 Definition of Adverse Events**

**Adverse Event (AE):** refers to any adverse medical event that occurs in a patient or clinical study patient. It does not necessarily have a causal relationship with medication. An adverse event may be an unfavorable sign (including abnormal laboratory tests, etc.), symptom, or disease that is not related to the purpose of the drug, regardless of causal relationship to the drug.

Any event that was new or that worsened in severity or frequency compared to the baseline conditions, including abnormal laboratory findings, were included here.

AEs do not include:

- Medical or surgical procedures should not be captured as AE terms. (e.g., surgery, endoscopy, tooth extraction, infusion), and the conditions leading to these procedures should be reported as AEs;
- Pre-existing diseases or conditions, including abnormal laboratory findings, that were present or detected but not aggravated prior to the start of study drug;

**7.1.1.2 Serious Adverse Event (SAE)**

A serious adverse event refers to the following adverse medical events (at any dose):

- Resulting in death
- Life-threatening

(indicates that the patient was at risk of death at the time of the event. Events that could theoretically lead to death if the adverse event was more severe)

- Requires hospitalization or prolonged hospitalization

**Note:** In general, hospitalization refers to the patient's stay in the hospital or emergency room (usually at least one night overnight) and/or receiving treatment that is not appropriate in the doctor's office or outpatient department. The complication that occurred during the hospitalization was an adverse event (AE). The event was serious if the complication prolonged the hospitalization or met any other serious criteria. This AE should also be considered serious when it is uncertain whether "hospitalization" or if "hospitalization" is required. Hospitalization for elective treatment or for pre-existing conditions that did not worsen since baseline was not an AE.

- Resulting in permanent or significant disability/loss of function

**Note:** The term disability refers to a significant impact on the ability of an individual to perform normal life functions. This definition does not include events of relatively small clinical significance, such as simple headache, nausea, vomiting, diarrhoea, influenza, and accidental trauma (e.g., ankle sprains) that may affect daily functioning but do not result in significant loss of function.

- Resulting in congenital malformations/birth defects in offspring
- Other important medical events

Other important medical events: medical and scientific judgment must be used in certain circumstances to decide whether to expedite reporting, such as important medical events that may not be immediately life-threatening, resulting in death or hospitalization, but are generally considered serious if medical action is required to prevent one of the above.

Note: The following hospitalizations are not considered serious adverse events because there are no "adverse events" related to the hospitalization (i.e., no adverse medical events):

- Temporary care hospitalization;
- Hospitalization for social reasons, e.g., for the convenience of care;
- Planned hospitalizations required by the protocol, e.g., hospitalizations for the administration of study drug or insertion of study drug or protocol-required laboratory tests;
- Hospitalization, elective surgery, or examination for pre-existing conditions planned prior to informed consent. (In this case, the condition requiring hospitalization does not worsen or develop into a new disease after the study drug administration and is documented in the original documentation);
- Hospitalization for routine maintenance (e.g., battery replacement) of devices that were in place prior to study participation.

**7.1.1.3 Adverse Drug Reaction (ADR)**

Refers to any harmful or undesirable reaction that may be related to the investigational product in a clinical trial. There is at least one reasonable possibility of a causal relationship between the investigational product and the adverse event, i.e. an association cannot be excluded.

**7.1.2 Collection and Recording of Adverse Events**

Starting point for AE/SAE collection records: self-signed ICF.

Note: Clinical adverse events that occur after the signing of the ICF and before the first dose are recorded as medical history/concomitant conditions in the CRF and are not recorded as AEs unless one of the following conditions is met:

- Any adverse event related to the procedures specified in the clinical study protocol (e.g., exercise testing, laboratory tests, etc.);
- Adverse events resulting from discontinuation of treatment related to the study regimen, such as changes or discontinuation of previous/concomitant treatments;
- Adverse events caused by drugs other than the investigational product taken as part of the treatment regimen.

Endpoint for AE/SAE collection records: 30 days after the last dose of study drug or the patient started other antineoplastic therapy, whichever occurs first.

Note: AEs/SAEs related to study drug after the AE/SAE collection recording endpoint should also be recorded.

Abnormal laboratory findings (e.g., routine blood or blood chemistry) or other abnormal assessments (e.g., ECG or vital signs) that are known not to be associated with the study disease as clinically significant by the investigator, will be recorded as an AE if the definition of an AE is met. If the seriousness criteria were met, it was reported as an SAE. During the trial, patients were advised to report any adverse events and trained staff were to ask patients about any adverse events in a non-induction manner at regular intervals. All adverse events directly observed or self-reported by the patient were reported by the investigator in concise language.

It is the responsibility of the investigator to examine all laboratory test results in all patients and determine if they constitute an adverse event. Medical and scientific judgment should be exercised in determining whether an individual laboratory test marked abnormality should be classified as an adverse event.

Adverse events should be reported truthfully during the trial, including the name of the adverse event, date of onset and end, severity, whether the AE is a serious adverse reaction, the causal relationship between the study drug and the event, the actions taken and the outcome of the adverse event, etc. On the basis of comprehensive consideration of comorbidities and concomitant medications, the correlation with the study drugs was evaluated. All adverse events occurring during the trial, including the washout period, will be recorded in the CRF.

In addition, the following data elements will be additionally collected for SAEs: date of AE escalation to SAE, date of AE escalation to SAE, reason for AE as SAE, hospitalization and discharge date, possible cause of death, date of death, autopsy results, SAE Assessment of causality with study procedures, event descriptions for AEs, etc.

**7.1.3 Adverse Event Assessment**

**7.1.3.1 Assessment of Relationship between Adverse Event and Study Drug:**

The investigator should assess whether the adverse event is related to study drug based on his/her knowledge of the patient and the context in which the event occurred, as well as any potential possible causes. The causality of an AE to the study drug was as follows:

| **It's certainly irrelevant:** | No study drug was used, or the time of the AE was not related to the use of study drug, or the cause of the AE was otherwise clear. |
| --- | --- |
| **May not be relevant:** | With evidence of use of study drug, the occurrence of AEs may be more explained by other causes. Negative or ambiguous withdrawal reaction. |
| **May be related:** | There was evidence of the use of study drug and the occurrence of AEs was reasonably related in time to the use of study drug. The AE could be explained by other causes. Positive withdrawal reaction. |
| **Likely related:** | There was evidence of the use of study drug and the occurrence of AEs was in a reasonable chronological order with the use of study drug. The explanation for AEs is more reasonable than that for other reasons. Positive withdrawal reaction. |
| **It's certainly relevant:** | There is evidence of the use of study drug, the occurrence of AEs and the use of study drug has a reasonable chronological sequence, and the explanation of AEs is more reasonable by study drug than by other reasons. Positive reaction to withdrawal and positive reaction to repeat if possible. |

Positively related, likely related, and possibly related are all listed as adverse drug reactions.

**7.1.3.2 Severity Assessment of Adverse Events**

The severity of adverse events was assessed using the NCI-CTCAE 5.0 Adverse Event Severity Grading Scale.

**Table 7-1 AE Severity Grading Scale**

| Level | Severity |
| --- | --- |
| Level 1 | mild; asymptomatic or mild; Clinical or diagnostic only; no treatment required. |
| Level 2 | Moderate; requires minor, local, or non-invasive treatment; Age-appropriate limitation of instrumental activities of daily living*. |
| Level 3 | Serious or medically important but not immediately life-threatening; Leading to hospitalization or extended stay  Hospital time; disability; Self-rational activities of daily living are limited**. |
| Level 4 | Life-threatening; requires urgent treatment. |
| Level 5 | Deaths related to AEs. |

*Instrumental activities of daily living refer to cooking, buying clothing, using the telephone, financial management, etc.

** Self-rational activities of daily life refer to bathing, dressing and undressing, eating, washing, taking medicine, etc., and are not bedridden.

**7.1.3.3 Anticipatory line assessment of adverse events**

An unexpected adverse reaction is the nature, severity, consequence, or frequency of an adverse reaction that is different from the expected risk described in the current relevant data (e.g., Investigator's Brochure, etc.) of the investigational product. The Investigator's Brochure serves as the primary document to provide reference safety information for determining whether an adverse reaction is expected or not.

**7.1.3.4 Suspected Unexpected Serious Adverse Reaction (Suspected Unexpected Serious Adverse Reaction, SUSAR)**

Refers to suspicious and unexpected serious adverse reactions whose clinical manifestations are of a nature and severity beyond the available information, such as the Investigator's Brochure, the label of the marketed drug, or the Summary of Product Characteristics.

**7.1.4 Reporting system for serious adverse events**

For any SAE during the trial, whether or not related to the study drug, the investigator should actively take appropriate measures to ensure the safety of the patient. The investigator should report the SAE to the sponsor within 24 hours of learning of the SAE. (The EDC system is preferred. If the EDC system is unavailable, report the problem by email at fsadedesk@fosunpharma.com.)

In the event of a death, the investigator should provide the sponsor and the Ethics Committee with other required information, such as the autopsy report and the final medical report.

After receiving the relevant safety information of the clinical trial provided by the sponsor, the investigator shall sign and read it in time, consider the patient's treatment, make corresponding adjustments, communicate with the patient as soon as possible if necessary, and report the suspicious and unexpected serious adverse reactions provided by the sponsor to the ethics committee.

The investigator will follow up the SAEs as required by the protocol and provide a detailed, written follow-up report within 24 hours of the availability of follow-up information in the same manner as above.

SAEs (including SUSARs) from clinical trials were evaluated and reported by the Sponsor or its representatives in accordance with the most recent and applicable regulatory requirements for clinical trials of drugs.

**7.1.5 Follow-up of Adverse Events**

The investigator should follow up each adverse event, all SAEs that are still present at the AE/SAE collection endpoint and related AEs, to:

- The event is resolved or restored to the state or stability at the time of the baseline.
- The investigator determined that there would be no further improvement;
- When more information is not possible to obtain. (The patient refused to provide more information or there was evidence that the patient was lost to follow-up even after best efforts were made).

The recovery time (date) of the adverse event during the study should be recorded in the adverse event eCRF and in the patient's medical records to verify the raw data.

For serious adverse events, adverse events of special interest, and pregnancy events, additional case information may be obtained by the Sponsor or other designee by telephone, fax, e-mail, and/or monitoring to allow for independent medical assessment of these reported cases.

The AE raw data updated after the database is locked shall be recorded for future use. The SAE information shall be filled in the Serious Adverse Event Report Form as detailed as possible and submitted to the PV department.

**7.1.6 Outcome of Adverse Events**

The investigator should determine the outcome of the adverse event based on the outcome of the patient's adverse event. The outcome of the adverse event was not as follows:

- Recovered: The patient fully recovered from the AE without any residual effects or harm.
- Improving: Signs and symptoms associated with the event have decreased, but not completely resolved.
- Recovered with sequelae: The patient has recovered, but with residual effects or injury. These residual effects may be temporary but still exist at the time of reporting. If sequelae is not considered permanent, additional information will be required at follow-up when the event changes.
- Unchanged: Signs and symptoms associated with the event did not abate and the patient's condition remained unchanged.
- Disease worsening: Signs and symptoms associated with the event did not abate and the patient's condition worsened.
- Death: "Death" can be selected as the outcome only for SAEs resulting in death. All other AEs/SAEs present at the time of death should be reported.
- Unknown: When the patient is lost to follow-up and the investigator cannot determine the outcome.

**7.1.7 Pregnancy Report**

For the sake of patient safety, if a female patient or a female partner of a male patient becomes pregnant during study drug treatment, a Pregnancy Report Form A must be completed and reported to the Sponsor within 24 hours of learning, and, The pregnancy must be followed up to determine the outcome of the pregnancy (if the male patient's female partner agrees to collect information), including spontaneous or induced abortion, details of delivery, congenital malformations, or maternal or neonatal complications, and reported by completion of Pregnancy Report Form B.

Spontaneous abortion, ectopic pregnancy, induced abortion for medical and health reasons, stillbirth, neonatal SAE (not limited to neonatal death, all congenital anomalies/birth defects) All reports were considered serious adverse events (SAEs) and SAEs were reported at the same time as the Pregnancy Report Form was completed and reported. Elective abortion without complications is not considered an AE. If other SAEs occur during pregnancy, SAEs must also be reported.

**7.1.8 Overdose**

If a patient develops symptoms as a result of an overdose (total daily dose or number of days in excess of the protocol), the overdose and all associated symptoms should be recorded on the AE page of the eCRF and reported promptly to the sponsor within 24 hours. Asymptomatic overdoses also require rapid notification within 24 hours. If serious consequences occur or the patient develops symptoms following an overdose that meet the assessment criteria for an SAE, the overdose should be reported as an SAE.

**7.1.9 Disease Progression**

Disease progression is defined as the worsening of a patient's condition due to a disease treated with the study drug. Disease progression may be an increase in the severity of the disease under study and/or an increase in disease symptoms. New metastases or progression of existing metastases in the primary tumor under study should be considered progressive disease and not an AE or SAE. Events determined to be due to disease progression during the study should not be reported as AEs.

Deaths that are clearly due to disease progression should be recorded in the eCRF and should not be reported as SAEs.

**7.2 Efficacy Assessment**

Tumors were evaluated according to the RECIST v1.1 criteria, and the imaging evaluation of tumors was at the discretion of the investigator, either with contrast-enhanced CT or MRI, but the evaluation methods were consistent throughout the study. The imaging results were interpreted by the investigator at each site. Tumor evaluation may be used as baseline tumor evaluation if it has been performed within 28 days prior to the first dose. Baseline tumor evaluation should include the chest, head, abdomen, pelvis, and any other site where tumor lesions are suspected. Any other known or suspected disease site may be examined by appropriate methods, such as a bone scan or a CT scan of the neck, if clinically indicated. Target lesion records: number, location, description, maximum diameter measurement per lesion (except lymph nodes) and minimum diameter measurement of lymph nodes, sum of diameters including all target lesions.

From the start of the dosing period C1D1, patients were assessed by radiographic methods every 2 cycles (42 days/56 days ±7 days) until disease progression, death, investigator decision, or patient voluntary withdrawal from the study. Additional imaging may be scheduled by the investigator based on the patient's clinical condition. Confirmatory assessments must be completed at least 4 weeks after the efficacy has been assessed as CR or PR according to RECIST v1.1. If the patient discontinued due to an AE or other reason, the tumor evaluation remained as scheduled. If the patient withdraws from the study due to disease progression, it is not necessary to repeat the radiographic assessment step at the final visit. If the patient discontinues study drug due to toxicity, tumor evaluations will continue as scheduled until disease progression. An unscheduled tumor assessment should be performed in cases where disease progression is suspected before the start of the next scheduled evaluation.

**7.3 Pharmacokinetic and Immunogenicity Assessment**

**7.3.1 Blood Sampling**

Refer to separate laboratory manuals for specific procedures, storage conditions, and shipping instructions for blood sampling at each blood collection time point. Concentrations of FS-1502 will be tested by a designated central laboratory. Results are presented in the form of a bioanalytical report.

The actual time of blood collection for each blood sample and all questions related to the collection and processing of the blood sample will be recorded in the eCRF.

**7.3.1.1 PK Blood Sampling**

To investigate the PK profile of FS-1502 in human serum, blood samples were collected and tested for concentrations of FS-1502, total antibody, and MMAF at the following time points:

**Patients with 28-day dosing cycle:**

Phase Ia 1st treatment cycle:

- D1: within 60 minutes before administration; 45 minutes (± 2 minutes) after the start of IV infusion, immediately after the end of administration (+ 2 minutes), 4 hours (± 3 minutes), 12 hours (± 3 minutes) after the end of administration;
- D2:24 hours after the end of administration (±0.5 hours);
- D3:48 hours after the end of administration (±0.5 hours);
- D4:72 hours after the end of administration (±0.5 hours);
- D5:96 hours after the end of administration (±0.5 hours);
- D6:120 hours after the end of administration (±0.5 hours);
- D8:168 hours after the end of administration (± 0.5 hours);
- D10:216 hours after the end of administration (±0.5 hours);
- D15:336 hours after the end of administration (±0.5 hours);
- D21:480 hours after the end of administration (±0.5 hours);
- D28:648 hours after the end of administration (±0.5 hours);

Phase Ia 3rd treatment cycle:

- D1: within 60 minutes before administration; 45 min (± 2 min) after the start of IV infusion, immediately after the end of administration (+ 2 min), 4 h (± 3 min), 12 h (± 3 min) after the end of administration;
- D2:24 h ±0.5 h after the end of administration;
- D3:48 h ±0.5 h after the end of administration;
- D4:72 h ±0.5 h after the end of administration;
- D5:96 h ±0.5 h after the end of administration;
- D6:120 h±0.5 h after the end of administration;
- D8:168 h ±0.5 h after the end of administration;
- D10:216 h ±0.5 h after the end of administration;
- D15:336 hours after the end of administration (±0.5 hours);
- D21:480 hours after the end of administration (±0.5 hours);
- D28:648 hours after the end of administration (±0.5 hours);

Phase Ia Cycle 4 and beyond:

- D1: within 60 minutes prior to administration.

**Patients in 21-day dosing cycle:**

Phase Ia 1st treatment cycle:

- D1: within 60 minutes before administration; Immediately after the end of dosing (+ 2 minutes), 4 hours after the end of dosing (±3 minutes);
- D2:24 hours after the end of administration (±0.5 hours);
- D3:48 hours after the end of administration (±0.5 hours);
- D4:72 hours after the end of administration (±0.5 hours);
- D6:120 hours after the end of administration (±0.5 hours);
- D8:168 hours (± 0.5 hours) after the end of administration;
- D10:216 hours after the end of administration (±0.5 hours);
- D12:264 hours after the end of administration (±0.5 hours);
- D15:336 hours after the end of administration (±0.5 hours);
- D18:408 hours after the end of administration (±0.5 hours);

Phase Ia 2nd treatment cycle:

- D1: within 60 minutes before administration;

Phase Ia 3rd treatment cycle:

- D1: within 60 minutes before administration; Immediately after the end of dosing (+ 2 minutes), 4 hours after the end of dosing (±3 minutes);
- D2:24 h ±0.5 h after the end of administration;
- D3:48 h ±0.5 h after the end of administration;
- D4:72 h ±0.5 h after the end of administration;
- D6:120 h ±0.5 h after the end of administration;
- D8:168 hours ±0.5 hours after the end of administration;
- D10:216 h ±0.5 h after the end of administration;
- D12:264 hours after the end of administration (±0.5 hours);
- D15:336 hours after the end of administration (±0.5 hours);
- D18:408 hours after the end of administration (±0.5 hours);

Phase Ia Cycle 4 and beyond:

- D1: within 60 minutes prior to administration.

**Stage Ib**

Blood will be collected for Pop PK analysis within 60 minutes prior to D1 dosing in each treatment cycle.

**7.3.1.2 Immunogenicity Inlet Point:**

Serum samples were collected prior to dosing on the first day of each treatment cycle and tested for anti-drug antibodies (ADA) until the last dose. Samples tested positive for ADA were tested for neutralizing antibodies (NAb).

**7.3.2 Pharmacokinetic Endpoints**

A noncompartmental model approach will be used to calculate the PK parameters for FS-1502 and MMAF for each dose group. PK parameters included:

AUC0- △, AUC0t, Cmax, Tmax, t1/2, Vd (or Vd/F), CL (or CL/F) and accumulation ratio (RAUC, RCmax), etc._‑_

**7.3.3 Handling and Transportation of Blood Samples**

The blood samples were not anticoagulated, centrifuged, seperated serum, subpackaged, and stored at 70°C for testing. (Biological sample processing and storage methods will be adjusted appropriately according to the results of prior stability studies). Transportation of blood samples: The samples were shipped in a dry ice package that can hold the volume for 3 days and transported in two separate steps from the sampling point to the testing center in a cold chain mode. For specific blood sample processing methods, refer to the sample processing manual provided by the testing party.−

**8 Data management**

**8.1 Data Entry**

Patient data will be entered into the designated electronic case report form system (eCRF) and transferred to a data system confirmed by the sponsor for integration with data from other sources.

Clinical data management will be performed in accordance with applicable CDISC standards and data cleansing procedures to ensure data integrity, such as the removal of errors and inconsistent data. Adverse events and concomitant medications will be named using the medical dictionary of standard names (MedDRA and WHODrug). The eCRF will be retained by the sponsor and a copy will be sent to the investigator as a copy.

Site staff will be responsible for completing the eCRF. For all patients who sign the ICF, the investigator or authorized staff shall carefully and carefully record any items in the eCRF, without empty or missing items. (If there is no record, fill in UK/NA/ND according to the actual situation.) All data in the eCRF must be checked against the patient's source data to ensure correctness.

The investigator shall attach the original test sheet or copy to the patient's study medical record; Abnormal laboratory or test data must be verified by the investigator and described as clinically significant; The investigator should complete the eCRF in strict accordance with the instructions for completion.

**8.2 Database Locking**

Data can be locked when all the following conditions are met:

1. All data has been entered into the database;
2. All doubts have been resolved;
3. Analysis populations were defined and judgments were made.

Locked data files will not be modified.

**9 Statistics and Statistical Analysis**

A statistical analysis plan should be developed after the protocol is finalized and finalized prior to database lock. The Statistical Analysis Plan will detail and describe all planned statistical analysis in accordance with the main characteristics of the protocol. This study focuses on descriptive statistical analysis.

All variables obtained at each observation time point were statistically described by dose group, unless the protocol determines that statistical description is not required at a specific time point. In general, continuous variables (e.g. age) will be described statistically using the number of observations, mean, median, standard deviation, minimum and maximum; Categorical variables will be statistically described using the frequency and percentage of each category. Continuous safety analyses were performed on DLTs and study drug-related adverse events and other safety measures to determine how to perform a dose climb. The final analysis for the study will be based on data collected by patients throughout the study. Statistical methods will be described in detail in the Statistical Plan.

**9.1 Calculation of sample size**

The dose ramping study follows a slow titration 3 + 3 pattern and is expected to enroll approximately 92 patients, including 21-42 in Phase Ia.

Phase Ib enrolls approximately 50 patients, assuming that the ORR for FS-1502 is 50% and the type I error level is one-sided 0.025 and the 95% confidence interval for ORR is (35.5 - 65.4%).

**9.2 Analysis Set**

**9.2.1 Phase Ia Analysis Set**

- DLT Analysis Set: Includes patients who experienced a DLT during the DLT observation period and patients who took at least 80% of the planned medication and completed the DLT observation period without a DLT.
- Safety Analysis Set: Any patient with at least one use of FS-1502.
- Efficacy Analysis Set: Patients with at least one FS-1502 use, baseline tumor assessment data, and at least one postbaseline tumor assessment data.
- PK Concentration Analysis Set: Includes patients who took FS-1502 at least once and had at least 1 PK blood sample collected as scheduled and with study drug concentration data available.
- PK Parameter Analysis Set: Patients who received medication per protocol and had at least 1 PK parameter during the trial. Significant protocol violations that affected the results of the PK parameters or could not be estimated were not included in the PK parameter analysis set.
- Immunogenicity Analysis Set: Includes patients with at least one FS-1502 use and at least one scheduled blood sample with ADA data.

**9.2.2 Phase Ib Analysis Set**

- Efficacy Analysis Set: Patients with at least one dose of FS-1502, and at least one postbaseline tumor assessment.
- Safety Analysis Set: Any patient with at least one use of FS-1502.
- PK Concentration Analysis Set: Includes patients who took FS-1502 at least once and had at least 1 PK blood sample collected as scheduled and with study drug concentration data available.
- PK Parameter Analysis Set: Patients who received medication per protocol and had at least 1 PK parameter during the trial. Significant protocol violations that affected the results of PK parameters or could not be estimated were not included in the PK parameter analysis set.
- Immunogenicity Analysis Set: Includes patients with at least one FS-1502 use and at least one blood sample as scheduled with ADA data.

**9.3 Safety Analysis**

Safety was evaluated by DLTs, AEs, laboratory findings, and changes in vital signs.

To assess the occurrence of DLTs and determine the MTD/RP2D. AEs will be summarized separately by single-dose phase, first cycle of continuous single-dose phase, and entire treatment period, and will be counted for treatment-related AEs, SAEs, AEs with toxicity grade ≥ 3, and AEs leading to discontinuation.

Changes in laboratory test results will be summarized according to the NCICTCAE version 5.0 standard grading. For laboratory measures, the maximum toxicity occurring during the study will be summarized in the form of counts and percentages. Descriptive statistics will be provided for changes in vital signs and ECOG scores compared to baseline levels.

The results of the safety data analysis and PK data analysis were reviewed periodically during the study to assess the safety of the study drug. The specific submission time depends on the project progress.

**9.4 Efficacy Analysis**

**9.4.1 Phase Ia**

Confirmed ORR will be calculated and ClopperPearson confidence intervals of 90% will be calculated in the efficacy and safety analysis sets. Survival analysis will be performed using the Kaplan-Meier method for PFS, DOR, and OS, and descriptive analysis will be performed for 1-year OS rate and DCR based on the investigator's assessment.

**9.4.2 Phase Ib**

Confirmed ORR will be calculated, and a Clopper Pearson 95% confidence interval will be calculated for the Efficacy and Safety Analysis Sets, respectively.

PFS and DOR were separately analyzed using the Kaplan-Meier method for survival, and 1-year OS rate and DCR were descriptively analyzed.

**9.5 Pharmacokinetic and Immunogenicity Analysis**

The PK data will be analyzed by PK analysis software using the PK concentration analysis set based on the individual concentration-time data collected for FS-1502, total antibody, and MMAF. PK analysis will strictly follow Standard Operating Procedures (SOPs). PK parameters included:

AUC0-∞, AUC0-t, Cmax, Tmax, t1/2, Vd (or Vd/F), CL (or CL/F) and accumulation ratio (RAUC, RCmax), etc.

Individual and mean concentrations of FS-1502, total antibody, and MMAF for each sampling point will be summarized by listing and descriptive statistics including arithmetic mean, minimum, median, maximum, standard deviation, coefficient of variation, and geometric coefficient of variation. In addition, individual and mean concentrations of FS-1502, total antibody, and MMAF were scatter plotted for each sampling point. The summary PK parameters will be counted by arithmetic mean, geometric mean, median, reference range, standard deviation, and coefficient of variation. Further details will be described in the Statistical Plan. Calculations of additional PK parameters, if required, will also be detailed in the statistical plan.

Concentration data obtained in this study or obtained in previous studies for the Pop PK analysis will be included in the Pop PK analysis and will be presented in a separate analysis report.

Blood samples were collected prior to dosing on the first day of each treatment cycle and tested for anti-drug antibodies (ADA) until the last dose was reached. Samples tested positive for ADA were tested for neutralizing antibodies (NAb). The positive rates of immunogenic anti-drug antibodies (ADA) and antibodies (NAb) were calculated and tabulated.

Preliminary analysis data will be sent in Excel to the responsible person responsible for validation of the PK methodology and interpretation of the results. The final results will be transferred to the data management system.

**9.6 Interim Analysis**

**9.6.1 Phase Ia**

This study plans to conduct an interim analysis after the end of the dose ramping study to assess the safety, metabolism, and preliminary antitumor activity of the drug.

**9.6.2 Phase Ib**

An interim analysis using Bayesian posterior probability will be conducted when approximately 20 patients have completed 2 tumor assessments. If the predicted Pr (ORR <20%) is >80%, i.e. fewer than 6 responses are observed in 20 evaluable patients, then there is an 80% probability that the drug ORR is lower than standard of care and early discontinuation of the cohort may be considered, otherwise continuing enrollment to approximately 50 patients. Bounds will be adjusted based on the actual number of people in the Efficacy Analysis Set. The interim analysis margins are non-binding and the sponsor will consider the safety and effectiveness data in the final decision.

**10 Study Administration**

**10.1 Ethical Considerations**

This study will be conducted in accordance with the ethical requirements of the Declaration of Helsinki (Fortaleza 2013), International Conference on Harmonisation (ICH) E6 Good Clinical Practice, national laws and regulations related to clinical research, and the implementation of this study protocol.

The study protocol, informed consent form, medical report form, and other materials must be submitted to the Ethics Committee for approval before the start of the study. The Ethics Committee will approve these materials in strict accordance with the requirements of relevant laws and regulations. The study can only be started after the approval of the Ethics Committee has been received.

During the course of the study, any modifications made to the protocol must be reviewed and approved by the Ethics Committee before implementation.

In addition, the Ethics Committee will approve all amendments to the clinical trial protocol (except administrative changes approved by the sponsor), informed consent forms and updates, patient recruitment procedures, written information provided to patients, available safety information, information on compensation and subsidies available to patients, biographical information of the investigator and /or other certification of qualifications, and any other documentation required by the Ethics Committee and the Regulatory Authority, if applicable.

**10.2 Informed Consent**

The investigator or his designated representative will be responsible for explaining to each patient, the patient's legal representative or fair witness the background of the study, the pharmacological characteristics of the study medication, the study protocol, and the benefits and risks of participating in the study. Written informed consent, signed by the patient or his/her legal representative and the study physician, should be obtained prior to the patient's entry into the study (before the screening examination).

The final ICF text should include the following: study objectives, study procedures, patient obligations, foreseeable benefits to the patient from participating in the study, and foreseeable risks and inconveniences; Treatment and appropriate insurance reimbursement available to the patient in the event of study-related injury; Access to study data and confidentiality of patient information, etc. The ICF should be approved in writing by the relevant regulatory authority in accordance with the regulations and in a language that can be read by the patient.

The ICF is to be signed and dated by the patient or his/her legal representative, the investigator performing the informed consent process, or his/her representative. The original ICF should be retained by the investigator and 1 copy by the patient. If important new data are found concerning the investigational drug, the ICF must be modified in writing and sent to the relevant regulatory authorities for approval, and informed consent must be obtained again.

**10.3 Protocol Amendment**

Any important amendment to this protocol must be approved by the Sponsor and the investigator in writing prior to implementation, and submitted to the Ethics Committee for approval and submission to the regulatory authorities for filing.

Any changes to the protocol will require a written protocol amendment, and administrative changes must be approved by the Sponsor prior to implementation. Changes that have a special impact on patient safety, scope of study, or scientific quality of the study will require a request to the regulatory authorities and approval by the appropriate ethics committee of each site. The above requirements shall not preclude immediate action by the Investigator or the Sponsor to protect the safety interests of all patients. If the investigator determines that a change or deviation to the protocol is immediately required for safety reasons to exclude the risk to the patient, the sponsor's medical monitor and the ethics committee of the site will be notified immediately. The sponsor must notify the regulatory authorities in accordance with local regulations.

Amendments to the protocol involving only administrative or administrative aspects of the study do not need to be submitted to the regulatory authority or the Ethics Committee, but should be communicated to the regulatory authority or the Ethics Committee in accordance with local regulations.

**10.4 Protocol Deviations**

The investigator must not deviate from the study protocol without formal clinical trial protocol amendments identified and approved by the appropriate ethics committee, except to eliminate immediate harm to the patient or when the change only relates to the administrative or administrative aspects of the study and is approved by the medical monitor and/or the sponsor.

All requirements specified in the protocol must be strictly enforced. Any intentional or unintentional deviation or violation of the protocol and GCP principles can be classified as a deviation or violation of the protocol, and the investigator or the investigator's designee is required to document and explain the details and reasons for the deviation/violation and to inform the regulatory authorities or the Ethics Committee in accordance with local regulations.

Specific protocol deviations or protocol violations are described in the Medical Monitoring Plan.

**10.5 Patient Confidentiality and Privacy**

The patient's personal information and privacy will be kept strictly confidential. Personal data such as the patient's name will be replaced by a code or number during the study. The collection and processing of personal data for patients in this study will be limited to those data necessary to investigate the efficacy, safety, tolerability, quality, and utility of the study drug. Such data must be collected and processed with adequate precautions to ensure confidentiality and compliance with appropriate data privacy protection laws and regulations. Monitors, other authorized representatives of the Sponsor, ethics committees, and representatives of regulatory authorities may access these patient records. The results of the study may be published in journals, but no personal information about the patient will be disclosed.

**10.6 Monitoring**

The investigator should allow the clinical monitor to inspect the clinic, laboratory, and pharmacy facilities, access to the case report form, informed consent form, and all source data to ensure that the study meets Good Clinical Practice and local regulatory requirements.

A designated clinical monitor will conduct monitoring visits to each site per the monitoring plan. An on-site visit will occur prior to the start of the study. Visits will be made at regular intervals during the conduct of the study. Contact may be made by phone, fax, or email as needed as a supplement to the on-site visit.

The investigator will be informed of the expected frequency of monitoring visits prior to the start of the study. In addition, the investigator will be notified in advance of each monitoring visit during the study. The purpose of the visit is to ensure that the clinical study is conducted in strict accordance with the study protocol; The completeness, accuracy, and verification of the medical report form from the source documents.

The clinical monitor shall verify that all medical record report forms are filled in correctly and completely and consistent with the original data; All errors or omissions were corrected or noted, signed and dated by the investigator. At each visit, close collaboration between the investigator and the clinical monitor is required to review and verify the case report form, drug supply and inventory records, drug dispensing and collection records, and any additional records arranged.

**10.7 Quality Assurance and Quality Control**

The sponsor, investigator and contract research organization shall perform their respective responsibilities in accordance with the requirements of GCP, strictly follow the test protocol and adopt the corresponding standard operating procedures to ensure the implementation of quality control and quality assurance system for clinical trials, sample testing, statistical calculation, etc.

To ensure the quality of the study, all researchers must be trained in the trial protocol before the trial is started, and all SOPs must be strictly implemented during the trial. The sponsor shall send qualified monitors to supervise the trial process and check the test data.

The sponsor may entrust inspectors to conduct systematic audits of trial-related activities and documents to review whether the trial is conducted in accordance with the trial protocol, standard operating procedures, relevant regulations and technical guidelines, and whether the test data is recorded and reported in a timely, true, accurate and complete manner. The audit should be performed by a person independent of the clinical trial.

The relevant data and documents (including medical records) of the research centers and laboratories participating in the clinical trial shall be inspected and verified by the drug supervision and administration department.

**10.8 Direct Access**

The Principal Investigator will provide direct access to source data and documentation for the Ethics Committee personnel, monitors, and other designated personnel of the Sponsor who conduct study-related monitoring and/or review. The purpose of the monitoring or audit is to systematically and independently examine all study-related activities and documentation, to determine whether they were conducted, and to record, analyze, and accurately report data in accordance with the protocol, GCP, ICH guidelines, and any appropriate regulatory requirements. The Principal Investigator will inform the Sponsor immediately if the Agency and/or Principal Investigator are contacted by the Regulatory Authority for verification.

The investigator must inform the patient that his/her study-related records can be reviewed by the above-mentioned individuals without violating the patient's personal health information privacy.

**10.9 Data Recording and Retention**

For the purposes of evaluation and supervision by regulatory authorities and the sponsor, the investigator shall agree to maintain all study data, including confirmation records for all patients. (Be able to effectively check all records, such as medical report forms and original hospital records). All original signed patient informed consent forms, all medical report forms, detailed records of drug distribution, etc. The shelf life is 5 years after the study drug is approved for marketing or until the time limit is agreed in the clinical contract and the sponsor is notified of destruction.

All data in this clinical study shall be owned by the sponsor and shall not be provided by the investigator to third parties in any form without the written consent of the sponsor, except as requested by the regulatory authorities.

**10.10 Insurance and Patient Reimbursement**

The Sponsor will provide insurance in connection with the clinical trial in accordance with all applicable laws and regulations.

The sponsor will bear the cost of treatment and the corresponding financial compensation for the injury that is causally related to the study due to the patient's participation in the study, except for medical malpractice.

**10.11 Storage and Use of Biological Specimens**

Biological samples for this study will be stored at the central laboratory designated for testing specimens for use only in this clinical study, destroyed upon completion of testing of the test blood sample, and stored for destruction until two years after the study drug is approved for marketing.

**10.12 Study Interruption and Early Termination**

The sponsor reserves the right to stop the study at any time for medical reasons or for any other reason. In the event of premature termination or interruption of the study, the sponsor shall immediately notify the investigator that the study has been terminated or interrupted and state the reason for the termination or interruption. As required by the relevant regulations, the sponsor or investigator shall also immediately notify the Ethics Committee of the termination or interruption of the study and explain the reason.

The investigator reserves the right to determine whether the study should be discontinued. If the investigator terminates or interrupts the study without the prior consent of the sponsor, the investigator shall immediately notify the sponsor and the ethics committee and provide the sponsor and the ethics committee with a detailed written explanation for the termination or interrupt. Study records must be kept.

**10.13 Study Summary Report**

After the end of the study, the investigator and the sponsor objectively summarize the study results, conduct statistical analysis on the study data with appropriate statistical methods, objectively evaluate the safety of the drug according to the results, and make a written summary report of the clinical study after the sponsor's review and approval.

**10.14 Information Disclosure and Data Publication Policy**

The investigator shall keep the information and data related to this study confidential and shall not quote or publish the results or materials without the consent of the sponsor.

The sponsor has the right to publish or publish information or data related to this study or to submit it to the regulatory authorities. The sponsor shall obtain the investigator's consent if the investigator's name is required to appear in the publication, publication, or advertisement.

**10.15 Conflict of Interest Statement**

The investigator will provide the sponsor with sufficient and accurate financial information as required by the relevant regulatory authorities so that the sponsor can submit complete and accurate financial statements or disclosure statements to the relevant regulatory authorities. The investigator will provide information on the relevant financial benefits during the study and for 1 year after the end of the study.

**Attachment 1 Pharmacokinetic and Immunogenic Blood Sample Collection Time and Volume**

| Study Scheduled Visits | | | Collection Time Point (h) | Time window (min) | Inlet Volume (ml) |  |
| --- | --- | --- | --- | --- | --- | --- |
| Phase Ia | Cycle 1/3 | D1 | Pre-dose intravenous drip | ≤60min | 10 (PK+Immunogenic Blood Collection) |  |
|  |  |  | 0.75** after intravenous drip | ±2min | 6 (PK blood collection) |  |
|  |  |  | Immediately after the end of intravenous drip administration | +2min | 6 (PK blood collection) |  |
|  |  |  | After the end of intravenous drip administration 4 | ±3min | 6 (PK blood collection) |  |
|  |  |  | 12 * * After the end of intravenous drip administration | ±3min | 6 (PK blood collection) |  |
|  |  | D2 | 24 after the end of intravenous drip administration | ±30 min | 6 (PK blood collection) |  |
|  |  | D3 | 48 after the end of intravenous drip administration | ±30 min | 6 (PK blood collection) |  |
|  |  | D4 | 72 after the end of intravenous drip administration | ±30 min | 6 (PK blood collection) |  |
|  |  | D5** | 96 after the end of intravenous drip administration | ±30 min | 6 (PK blood collection) |  |
|  |  | D6 | 120 after the end of intravenous drip administration | ±30 min | 6 (PK blood collection) |  |
|  |  | D8 | After the end of intravenous drip 168 | ±30 min | 6 (PK blood collection) |  |
|  |  | D10 | After the end of intravenous drip 216 | ±30 min | 6 (PK blood collection) |  |
|  |  | D12* | 264 after the end of intravenous drip administration | ±30 min | 6 (PK blood collection) |  |
|  |  | D15 | 336 after the end of intravenous drip administration | ±30 min | 6 (PK blood collection) |  |
|  |  | D18* | 408 after the end of intravenous drip administration | ±30 min | 6 (PK blood collection) |  |
|  |  | D21** | 480 after the end of intravenous drip administration | ±30 min | 6 (PK blood collection) |  |
|  |  | D28** | 648 after the end of intravenous drip administration | ±30 min | 6 (PK blood collection) |  |
|  | Cycle 2 | D1* | Pre-dose intravenous drip | ≤60min | 10 (PK+Immunogenic Blood Collection) |  |
|  |  | D1** | Pre-dose intravenous drip | ≤60min | 4 (Immunogenic blood sampling) |  |
|  | Cycle 4 and beyond | D1 | Pre-dose intravenous drip | ≤60min | 10 (PK+Immunogenic Blood Collection) |  |
| Stage Ib | Each dosing cycle | D1 | Pre-dose intravenous drip | ≤60min | 10 (PK+Immunogenic Blood Collection) |  |
|  | Note: | | - Blood samples were collected prior to dosing on D1 in each cycle and tested for anti-drug antibodies (ADAs) until the last dose was reached. Samples tested positive for ADA were tested for neutralizing antibodies (NAb). - *Only for patients with 21-day dosing cycle; * *Only for patients with 28-day dosing cycle. - The blood inlet volume for PK analysis was 6 mL and the blood inlet volume for immunogenicity was 4 mL. | | | |

Note: It is best to maintain intravenous drip administration at the same time every day during treatment.

**Attachment 2 Assessment Form for the Dose Uphill Phase**

|  | Screening Period | Cycle 1 1 | | | | | Cycle 2 1 | | Cycle 3  and later 1  ± 2 days | End of treatment  ± 7 days | Follow-up 30 days after end of treatment ±7 days 12 | Survival follow-up  Once every 3 months  ± 7 days 10 |
| --- | --- | --- | --- | --- | --- | --- | --- | --- | --- | --- | --- | --- |
|  |  | All patients | | | For 28-day 1 cycle only | |  |  |  |  |  |  |
|  | D-28 to  D-1 | D1 to D5 | D8  ± 1 day | D15  ± 1 day | D21  ± 1 day | D28 | D1  ± 2 days | D15  ± 2 days |  |  |  |  |
| Informed Consent | X |  |  |  |  |  |  |  |  |  |  |  |
| Demographics | X |  |  |  |  |  |  |  |  |  |  |  |
| Exclusion criteria  Assessment | X |  |  |  |  |  |  |  |  |  |  |  |
| Past medical history | X |  |  |  |  |  |  |  |  |  |  |  |
| history of tumor disease | X |  |  |  |  |  |  |  |  |  |  |  |
| Tumor Assessment 11 (RECIST1.1) | X |  |  |  |  |  | X |  | X | X |  |  |
| General physical examination | X | X |  |  |  |  | X |  | X | X | X |  |
| Vital Signs 2 and ECOG Score | X | X | X | X | X |  | X | X | X | X | X |  |
| Cardiac ultrasound/cardiac function classification | X |  |  | X |  |  | X |  | X | X | X |  |
| Electrocardiogram 3 | X | X | X | X | X |  | X | X | X | X | X |  |
| Routine blood test 4 | X | X | X | X | X |  | X | X | X | X | X |  |
| Blood Chemistry 4 | X | X |  | X |  |  | X |  | X | X | X |  |
| Coagulation 5 | X | X |  |  |  |  | X (as clinically needed) |  | X (as clinically needed) | X | X (as clinically required) |  |
| Urine routine 6 | X | X |  |  |  |  | X |  | X | X | X |  |
| PK and Immunogenicity Blood Collection 7 |  | X | | | | | | | |  |  |  |
| Blood HCG (if applicable) | X |  |  |  |  |  |  |  |  | X |  |  |
| HBV (quantitative DNA test if surface antigen positive), HCV and HIV | X |  |  |  |  |  |  |  |  |  |  |  |
| Tumor tissue HER2 | X |  |  |  |  |  |  |  |  |  |  |  |
| DLT Assessment |  | X | | | | |  |  |  |  |  |  |
| Concomitant medication/treatment 8 | X | | | | | | | | | | |  |
| Evaluation of AEs and SAEs 9 | X | | | | | | | | | | |  |
| Patient Dosing 13 |  | X |  |  |  |  | X |  | X | X |  |  |
| Survival and antitumor status |  |  |  |  |  |  |  |  |  |  |  | X |

**Note:** 1. Patients enrolled according to protocol version 1.2 and version 2.0:28 days is a cycle; Patients enrolled in the Version 3.0 and Version 4.0 protocol: 21 days as 1 cycle;

2. Vital signs include blood pressure, heart rate, breathing, and temperature. Blood pressure was measured 5 minutes after the patient sat; On the day of dosing for the first three cycles: within 1 hour prior to dosing, intravenous infusion at 15 minutes (± 5 minutes), 30 minutes (± 5 minutes), 60 minutes (± 5 minutes) or 120 minutes (± 10 minutes) at the end of intravenous infusion, If there are clinical symptoms related to infusion reactions, please check at any time; If the patient has not had an infusion response in the first three cycles, only the vital signs 30 minutes before and at the end of dosing may be considered after cycle 4;

3. ECG is performed during the screening period, before each treatment cycle. If the test results within 7 days prior to Day 1 are available, it is not necessary to repeat the test before the first dose on Day 1;

4. Routine blood test and blood chemistry test. If the test results within 7 days before Day 1 are available, it is not necessary to repeat the test before the first administration on Day 1;

5. Coagulation function test, which is mandatory during the screening visit and the end-of-treatment visit; If the results of the screening period are not clinically significant, repeat testing on the day of the first dose is not required, and testing may be performed thereafter as clinically required;

6. Urine routine examination. If the results of the examination within 7 days before Day 1 are available, it is not necessary to repeat the examination before the first administration on Day 1;

7. For details of PK blood collection and immunogenicity, refer to Attachment 1.

8. Concomitant medication/treatment records from 28 days prior to the first dose until 30 days after the last dose of study medication or until the start of other antineoplastic therapy, whichever occurs first;

9. AEs and SAEs were recorded from the time the informed consent was signed until 30 days after the end of study treatment or until the start of other antitumor therapy, whichever occurred first;

10. Starting from the safety visit or the end-of-treatment visit (whichever occurs later), the patient underwent survival follow-up and subsequent treatment follow-up, every 3 months (± 7 days) by phone, text message, or other remote means until the patient died, and the patient withdrew consent. Lost to follow-up or end of trial;

11. Tumors were evaluated according to RECIST v1.1 criteria. The imaging evaluation of the tumor is at the discretion of the investigator, either CT or MRI, and if there are no contraindications, contrast media should be used. The imaging results were interpreted by the investigator or radiologist at each site and were considered as a baseline tumor evaluation if a tumor evaluation was performed within 28 days prior to the first dose. Baseline tumor evaluation should include the head, chest, abdomen, pelvis, and any other site where tumor lesions are suspected. Tumors were assessed by imaging methods every 2 cycles (42/56 ±7 days) starting on Day 1 of the continuous dosing period until disease progression, withdrawal of consent, or death, and the tumor assessment schedule was not affected by discontinuation. For patients with bone metastases, the investigator performed bone scans to follow up the lesions as clinically required. Additional imaging studies may be scheduled by the investigator based on the patient's clinical conditions. If the patient discontinued due to an AE or other reason, tumor evaluations remained as scheduled until disease progression. If the patient withdraws from the study due to disease progression, it is not necessary to repeat the radiographic assessment step at the final visit. For cases where disease progression is suspected before the start of the next scheduled evaluation, an unscheduled tumor assessment should be performed.

12. The last posttreatment visit needs to be completed before the start of other treatments, and if the patient has started other treatments without the knowledge of the investigator, the last visit is not required; If the patient is unable to return to the study site for the final visit due to a medical condition, a telephone visit may be made and the results of the local examination may be received.

13. The study drug should be administered after the completion of the assessment including laboratory tests, etc., confirming that the patient is safe to continue administering the study drug.

14. End of treatment refers to the day on which the investigator determines that the patient will not be given the study drug. The End of Treatment visit should be completed within 7 days after the end of treatment. If the corresponding laboratory tests were performed within 7 days prior to the End of Treatment visit, it does not need to be re-examined at the End of Treatment visit. Tumor imaging assessments should be performed at the End of Treatment Visit and need not be repeated at the End of Treatment Visit if the interval between the last assessment is less than 4 weeks.

**Attachment 3 Dose Expansion Phase Assessment Form**

|  | Screening Period  D-28-D-1 | Study Treatment Period 1  CXD1  ± 2 days | End of treatment  ± 7 days 14 | | End of treatment  After 30 days ±7 days 12 | Survival Follow-up 10  Every 3 months  ± 7 days |
| --- | --- | --- | --- | --- | --- | --- |
| **Informed Consent** | X |  |  | |  |  |
| **Demographics** | X |  |  | |  |  |
| **Assessment of Study Enrollment/Exclusion Criteria** | X |  |  | |  |  |
| **Past medical history** | X |  |  | |  |  |
| **History of tumor disease** | X |  |  | |  |  |
| **Tumor Assessment (RECIST v1.1) 11** | X | X | X | |  |  |
| **General Physical Examination 15** | X | X | X | | X |  |
| **Vital signs and ECOG score 2** | X | X | X | | X |  |
| **Cardiac ultrasound/cardiac function classification** | X | X (as clinically needed) | X (as clinically needed) | | X (as clinically required) |  |
| **12-lead ECG 3** | X | X | X | | X |  |
| **Blood routine blood chemistry test 4** | X | X | X | | X |  |
| **HBV (quantitative DNA test if surface antigen is positive), HCV and HIV** | X |  |  | |  |  |
| **Coagulation 5** | X | X (as clinically required) | X | | X (as clinically needed) |  |
| **Ophthalmological examination (visual and slit lamp examination)** | X | X | X | | X |  |
| **Urine routine 6** | X | X | X | | X |  |
| **PK and Immunogenicity Blood Collection 7** |  | X | |  |  |  |
| **Concomitant medication/treatment 8** | X | X | X | | X |  |
| **AE and SAE Assessment 9** | X | X | | | |  |
| **Tumor tissue HER2** | X |  |  | |  |  |
| **Blood HCG (if applicable)** | X |  | X | |  |  |
| **Patient Dosing 13** |  | X | X | |  |  |
| **Survival and antineoplastic treatment** |  |  |  | |  | X |

**Note:** 1.21 days is one treatment cycle;

2. Vital signs include blood pressure, heart rate, respiration, and temperature. Blood pressure was measured 5 minutes after the patient sat;

3. ECG was performed during the Screening Period, prior to each treatment cycle. If the test results within 7 days prior to dosing are available, it is not necessary to recheck before dosing;

4. Routine blood examination and blood biochemical examination. If the results of the examination within 7 days before administration are available, it is not necessary to recheck before administration;

5. Coagulation function test is mandatory during the screening visit and the end-of-treatment visit. If the screening test results are clinically unremarkable

Meaning, the first dose does not need to be repeated on the same day, and the test can be carried out according to clinical needs thereafter;

6. Urine routine examination. If the results of the examination within 7 days before Day 1 are available, it is not necessary to repeat the examination before the first administration on Day 1;

7. PK and immunogenicity blood samples were collected before dosing in each cycle, see Attachment 1 for details.

8. Concomitant medications were recorded from 28 days prior to the first dose until 30 days after the last dose of study medication or until the start of other antineoplastic therapy, whichever occurs first;

9. AEs and SAEs were recorded from the time the informed consent was signed until 30 days after the end of study treatment or until the start of other antitumor therapy, whichever occurred first;

10. Starting from the safety visit or the end-of-treatment visit (whichever occurs later), the patient underwent survival follow-up and subsequent treatment follow-up, every 3 months (± 7 days) by phone, text message, or other remote means until the patient died and the patient withdrew consent. Lost to Follow-up or End of Trial

11. Tumors were evaluated according to RECIST v1.1 criteria. The imaging evaluation of the tumor was at the discretion of the investigator, either CT or MRI, and if there are no contraindications, contrast media should be used. Imaging results were interpreted by the investigator or radiologist at each site and were considered to be the baseline tumor evaluation if a tumor evaluation had been performed within 28 days prior to the first dose. Baseline tumor evaluation should include the head, chest, abdomen, pelvis, and any other site where tumor lesions are suspected. Tumors were assessed by imaging methods every 2 cycles (42/56 days ±7 days) starting on Day 1 of the continuous dosing period until disease progression, withdrawal of consent, or death, and the tumor assessment schedule was not affected by discontinuation. For patients with bone metastases, the investigator performed bone scans to follow up the lesions as clinically required. Additional imaging may be scheduled by the investigator based on the patient's clinical condition. If the patient discontinued due to an AE or other reason, the tumor evaluation remained as scheduled. If the patient withdraws from the study due to disease progression, it is not necessary to repeat the radiographic assessment at the final visit. In cases where disease progression is suspected before the start of the next scheduled evaluation, an unscheduled tumor assessment should be performed.

12. The last posttreatment visit needs to be completed before the start of other treatments, and if the patient has already started other treatments without the knowledge of the investigator, the last visit is not required; If the patient is unable to return to the site for the final visit due to a medical condition, a telephone visit may be made and the results of the local examination may be received.

13. The study drug should be administered after the completion of an assessment including laboratory tests, etc., confirming that the patient is safe to continue administration of the study drug.

14. End of treatment refers to the day on which the investigator determines that the patient will not be given the study drug. The End of Treatment Visit shall be completed within 7 days after the end of treatment. If the corresponding laboratory tests have been performed within 7 days prior to the End of Treatment Visit, it does not need to be re-examined at the End of Treatment Visit. Tumor imaging assessments should be performed at the End of Treatment Visit and need not be repeated at the End of Treatment Visit if less than 4 weeks have elapsed since the last assessment.

15. For physical examination, height and weight will be measured at screening and weight will be measured only before dosing for subsequent treatment cycles, and dosing will need to be recalculated if the change in weight from baseline is greater than 10% (excluding edema).

**Attachment 4 ECOG Performance Status**

| Graded | ECOG |
| --- | --- |
| 0 | Completely normal and able to perform all normal activities without restrictions |
| 1 | Can't engage in vigorous physical activity, but can walk, and be able to engage in light physical activities or office work |
| 2 | Can walk, live self-catering, but can not do any work, daytime bedridden time not more than 50% |
| 3 | Life is barely self-catering, with more than 50% of the day in bed or in a chair. |
| 4 | Totally incapacitated, severely unable to take care of yourself, must be bedridden or wheelchair-bound |
| 5 | Death |

**Attachment 5 New York Cardiology Association Cardiac Function Classification (NYHA)**

| Level | Physical activity | resting state | Symptoms (fatigue, palpitations, wheezing, or angina) |
| --- | --- | --- | --- |
| I | Not restricted | Asymptomatic | General physical activity does not cause |
| II | Slightly restricted | Asymptomatic | Daily physical activity can cause |
| III | Obvious limitation | Asymptomatic | Caused by lower than daily physical activity |
| IV | lost | Symptomatic | Increase in any physical activity |

**Appendix 6 Medications that May Prolong QTc**

The following table lists drugs that are known to prolong QTc. This list does not include all drugs. For specific information on whether known compounds prolong QTc, please refer to the individual drug package insert.

| Medication Category | Generic Drug Name |
| --- | --- |
| Class IA antiarrhythmic drugs | quinidine procainamide pyramide |
| Class IC antiarrhythmic drugs | flecainide propafenone moroxizine |
| Class III antiarrhythmic drugs | amiodarone sotalol bromobenzylammonium ibutilide dofetilide |
| antipsychotic drugs | thiolidazine, mesodazine, chlorpromazine, prochlorperazine, trifluoperazine  Flufenacin, pimozitlipidone, ziprasidone  haloperidol |
| tricyclic/tetracyclic antidepressants | amitil, cypamin, doxepin, thiapine, promipramine  Maprotiline |
| Selective serotonin and norepinephrine reuptake inhibitors (SSNRIs) antidepressant | Venlafaxine |
| macrolide antibiotics | azithromycin erythromycin clarithromycin dierythromycin  Roxithromycin Tolamycin |
| fluoroquinolone antibiotics | Moxifloxacin plus tifloxacin |
| azole antifungal | Ketoconazole, fluconazole, itraconazole, posaconazole, voriconazole |
| antimalarial drugs | amodiaquine atovaquinone chloroquine doxycycline  halofontere mefloquine chloroguanidine primaquine  pyrimethamine, quinine, sulfadoxine |
| antigenic insect medicine | Pentamidine |
| Antiemetics | haloperidol dola setron granisetron ondansetron |
| anti-estrogen | tamoxifen |
| immunosuppressant | tacrolimus |

**A change summary of FS-1502 protocol amendments**

| **V1.0 to V2.0** | 1.Modification of the Study End Definition: In consideration of the survival time of patients with advanced tumors and the research plan, the definition of the study end is revised to: either two years after the start of treatment for the last patient or the end of treatment for the last patient, whichever occurs first.  2.Modification of Inclusion Criterion 5: The creatinine clearance rate is adjusted from a minimum of ≥ 60 mL/minute to a minimum of ≥ 45 mL/minute. No impact on renal function was observed in preclinical studies and in patients enrolled in the early stages, allowing more patients to be eligible for the study medication.  3.Modification of Exclusion Criterion 1: The washout period for chemotherapy and radiotherapy is revised from 4 weeks to 2 weeks. This change aims to reduce the risk of tumor progression due to an extended halt in chemotherapy or radiotherapy, taking into account the duration of the anti-tumor effects and the persistence of adverse effects associated with chemotherapy or radiotherapy. Therefore, the interval before drug administration after receiving chemotherapy or radiotherapy is shortened to 2 weeks.  4.Safety Measures: To ensure the safety of the subjects, the following additional tests are scheduled during the first cycle of medication: On Day 8 (D8): vital signs, complete blood count (CBC), blood chemistry, electrocardiogram (ECG); On Day 15 (D15): vital signs, CBC, blood chemistry, ECG, and echocardiogram; On Day 21 (D21): vital signs, CBC, blood chemistry, ECG. |
| --- | --- |
| **V2.0 to V3.0** | 1.Dosing Interval Adjustment for FS-1502: The dosing interval of FS-1502 has been changed from "once every 4 weeks" to "once every 3 weeks." Corresponding adjustments have been made to the DLT (Dose-Limiting Toxicity) observation period, tumor assessment intervals, pharmacokinetic (PK) blood sampling points, and laboratory test timings.  2.Added: If the number of patients screened and qualified for a particular dose group exceeded three, patients beyond the third are allowed to be enrolled into the study. These expanded patients are not included in the DLT assessment.  3.Clarification for Ib Phase Enrollment: The Ib phase will enroll 50 cases of HER2-positive breast cancer patients who have previously failed trastuzumab treatment (including those who relapsed after adjuvant chemotherapy, and patients with locally advanced or metastatic breast cancer).  4.Dose Adjustment During DLT Observation: No dose adjustments are permitted during the DLT observation period. After the clinical phase II recommended dose is obtained, investigators may adjust the dose to the recommended level for patients in the Ia phase who continue treatment within the trial group, provided that the clinical benefit for the patient is observed and the patient's informed consent has been obtained.  5.Standardization of Adverse Event Definitions: The definitions of adverse events (AEs) and serious adverse events (SAEs) are standardized. The collection start and end points, follow-up, and outcomes of AEs are clearly defined to provide a reference for the collection process.  6.Reporting System Update for SAEs: The reporting system for serious adverse events is updated in accordance with the requirements of the new "Good Clinical Practice (GCP)" guidelines.  7.Clarification of Reporting for Pregnancy and Disease Progression: The methods for reporting pregnancy and disease progression are clarified. |
| **V3.0 to V4.0** | 1.Addition of Specific Immunogenicity Parameters and Analysis Methods: Specific parameters for immunogenicity have been added along with the methods for immunogenicity analysis. Immunogenicity parameters include: Anti-drug antibodies (ADA) against FS-1502 and neutralizing antibodies (NAb). For immunogenicity analysis, the positive rates for ADA and neutralizing antibodies (NAb) are compiled.  2.Modification of the Definition of Renal Toxicity-Related DLT: The definition of dose-limiting toxicity (DLT) related to renal toxicity has been revised to grade 3 or higher renal toxicity that does not recover within 7 days (including renal hematuria, proteinuria, and a decrease in creatinine clearance rate). The original criteria was grade 2 or higher, which was considered too stringent.  3.Addition of PK Blood Sampling Points in the Ib Phase: Additional pharmacokinetic (PK) blood sampling points have been added for the Ib phase. This is to ensure the smooth progression of the study once the recommended dose is determined, based on the stage of research advancement. |
| **V4.0 to V5.0** | 1.Clarification of Recommended Phase 2 Dose (RP2D): The main content and decisions of the Safety Monitoring Committee (SMC) meeting are introduced. Based on the efficacy, safety, and pharmacokinetic (PK) data from the dose-escalation phase, a dose of 2.3mg/kg administered every 3 weeks (q3w) has been determined as the recommended dose.  2.Change in Dosage Form of the Study Drug: According to the progress in Chemistry, Manufacturing, and Control (CMC), the dosage form of the study drug has been changed from a frozen solution to a lyophilized (freeze-dried) powder.  3.Modification of the Study End Definition: The study end is redefined as one year after the start of treatment for the last enrolled patient or when 50% of the patients experience a death event, whichever comes first. Considering that patients with advanced breast cancer who have undergone multiple lines of treatment have a relatively short median progression-free survival time, the study will follow up until 50% of the total survival events have occurred. At that point, the data will be mature, and median Progression-Free Survival (PFS) and Overall Survival (OS) data will have been obtained, hence the adjustment to the study end definition.  4.Modification of PK Blood Sampling Purpose: Due to the Ib phase involving only sparse blood sampling for population pharmacokinetic analysis, the purpose of PK blood sampling has been modified to evaluate the population pharmacokinetic (Pop PK) characteristics and immunogenicity of continuous intravenous infusion of FS-1502 monotherapy in patients with HER2-positive locally advanced or metastatic breast cancer.  5.Modification of Dose Adjustment Principles: The minimum dose must not be lower than 1.0mg/kg. Efficacy data from the Ia phase indicate that there is no therapeutic effect below this dosage level.  6.Modification of the Ib Phase Enrollment Population: The enrollment criteria for the Ib phase have been refined to include patients with high HER2 expression who have failed anti-HER2 treatment and have previously received at least two lines of treatment for advanced breast cancer. This clarification of the enrollment population aims to explore whether the preliminary efficacy meets the requirements for single-arm registration for end-line treatment.  7.Modification of Inclusion Criterion 5: The neutrophil count requirement has been adjusted from ≥1.5×10^9/L to ≥1.0×10^9/L, as a decrease in neutrophils was not commonly observed in the initial data. The creatinine clearance rate has been revised from ≥45 mL/minute to ≥60 mL/minute, with the addition of urine protein ≤1+ or a 24-hour urine protein quantification of <1.0g to better protect the patients' renal function.  8.Addition of Exclusion Criterion 7: The presence of corneal epithelial lesions (except for mild punctate keratopathy) is added as an exclusion criterion. Considering the dry eye symptoms observed in patients in the Ia phase and the prevention and management principles of ocular toxicity associated with ADC (Antibody-Drug Conjugate) drugs already on the market, this exclusion criterion has been added.  9.Addition of Expected ORR for Ib Phase: An expected Objective Response Rate (ORR) of 50% is added for the Ib phase, with a 95% confidence interval of (35.5-64.5%), making the study design more comprehensive.  10.Addition of Safety Management and Dose Adjustment Measures for Ocular Toxicity, Hypokalemia, and Proteinuria: To better protect the safety of the subjects, measures for the management of ocular toxicity, hypokalemia, and proteinuria have been added. |
| **V5.0 to V6.0** | 1.Adjustment of Estimated Enrollment: The estimated number of participants was adjusted to 200 based on the study's enrollment figures and the subsequent expected development strategy. Later, due to adjustments in the R&D strategy, the actual number of enrolled participants was 150.  2.Expansion Enrollment in Ia Phase: The content has been expanded to include additional enrollment at the recommended dose for efficacy exploration during the Ia phase.  3.Creatinine Clearance Rate Adjustment: The creatinine clearance rate criterion has been adjusted from a minimum of ≥ 60 mL/minute to ≥ 50 mL/minute, reducing the restriction in line with clinical realities to allow more patients to be eligible for treatment.  4.Exclusion Criterion 7: The contraception period has been extended from 30 days after the last dose of medication to 3 months to better protect the safety of the subjects.  5.Removal of an Exclusion Criterion: The criterion for uncontrolled stable diabetes has been removed as no impact on blood sugar levels was observed.  6.Addition of an Exclusion Criterion: Uncontrolled stable pleural effusion, ascites, and pericardial effusion have been added as exclusion criteria. Patients with these conditions have a poor prognosis, and their inclusion could affect the treatment's efficacy.  7.Addition of an Exclusion Criterion: A history of myocardial infarction within the past 6 months has been added as an exclusion criterion to better protect the rights and safety of the subjects.  8.Addition of an Exclusion Criterion: Participants who have been diagnosed with any other malignant tumor within 3 years prior to the study are excluded, except for early-stage malignant tumors (carcinoma in situ or stage I tumors) that have been radically treated, such as adequately treated basal cell or squamous cell skin cancer or cervical carcinoma in situ. This avoids interference with the study data.  9.Modification of Interim Analysis Strategy: Based on the development strategy and plan, the interim analysis strategy has been modified. An interim analysis will be conducted once a certain amount of data has been accumulated, and the sponsor will decide whether to continue the trial based on a comprehensive consideration of safety and efficacy data.  The frequentist method will be used, instead of the Bayesian posterior probability, for the interim analysis.  10.Clarification of Ophthalmic Examinations During Treatment: The content related to ophthalmic examinations during the treatment period has been clarified.  11.Modification of AST/ALT Elevation and QTc Prolongation Management Principles: The principles for managing elevated AST/ALT levels and prolonged QTc intervals have been revised in accordance with clinical practice.  12.Addition of Ocular Toxicity Risk Management Plan: Content has been added to the ocular toxicity risk management plan annex to better manage ocular-related adverse events. |

| **FS-1502 Protocol V6.0 Amendment** | |
| --- | --- |
|  |  |
| **Protocol No.:** | **FS-CY1502-Ph1-01** |
| **Protocol title:** | **A multicenter, open-label, single-arm phase Ia/Ib clinical study: a dose-finding study of FS-1502 in patients with HER2-expressing advanced malignant solid tumors and a dose-expansion study in patients with HER2-positive locally advanced or metastatic breast cancer** |
| **Original Protocol Number and Date:** | **Protocol No. FS-CY1502-Ph1-01, Version 5.0, Version Date January 27, 2022**  **Protocol No. FS-CY1502-Ph1-01, Version 6.0, Version Date Aug 20, 2022.** |
| **Amendment Protocol No. and Date:**  **File Date:** | **August 29, 2022** |

| **Original content** | **Revision Content** | **Reason for Revision** |
| --- | --- | --- |
| **Page 7**  **Number of patients: Approximately 92 patients expected to be enrolled**  **Clinical Research Centers: Ia: 1-5 Ib: 1 - 10** | **Page 7**  **Number of patients: Approximately 200 patients expected to be enrolled**  **Clinical Research Centers: 1-12** | Increased number of patients and sites |
| **Page 7, 41**  **Study Period:**  **Stage Ia:**   - FS-1502 single-agent continuous administration: - Patients enrolled based on the 1.2 and 2.0 protocols: IV every 4 weeks; - Patients enrolled based on the Version 3.0 and Version 4.0 regimens: intravenous drip every 3 weeks starting at 1.0 mg/kg; | **Page 7, 42**  **Study Period:**  **Stage Ia:**   - FS-1502 single-agent continuous administration: - Patients enrolled based on the 1.2 and 2.0 protocols: IV every 4 weeks; - Patients enrolled based on Version 3.0 and Version 4.0 regimens: intravenous drip every 3 weeks starting at 1.0 mg/kg - Patients enrolled based on the 5.0 or higher protocol: 2.3 mg/kg intravenous drip every 3 weeks; | After the recommended dose has been determined, Phase Ia continues to expand enrollment at the same dose as Phase Ib. |
| **Page 9**  **Study Design:**  According to the main dose level designed in the scheme, different volumes of FS-1502 were drawn, added into 100 ml of 0.9% normal saline, and the infusion was completed within 60-90 minutes. The frequency and treatment period of FS-1502 were as follows:   - Patients enrolled based on the protocol version 1.2-2.0: intravenous drip every 4 weeks; - Based on Version 3.0 and Version 4.0 regimens: dosing by intravenous drip every 3 weeks starting at 1.0 mg/kg; | **Page 9**  **Study Design:**  According to the main dose level designed in the scheme, different volumes of FS-1502 were drawn, added into 100 ml of 0.9% normal saline, and the infusion was completed within 60-90 minutes. The frequency and treatment period of FS-1502 were as follows:   - Patients enrolled based on the protocol version 1.2-2.0: intravenous drip every 4 weeks; - Based on Version 3.0 and Version 4.0 regimens: dosing by intravenous drip every 3 weeks starting at 1.0 mg/kg; - Patients enrolled based on the protocol version 5.0 and higher: 2.3 mg/kg intravenous drip every 3 weeks; | As above. |
| **Pages 10, 12, 44**  Stage Ib: HER2 overexpression | **Pages 10, 12, 45**  Stage Ib: HER2 positive | According to the classification description of the guideline. |
| **Pages 12, 44**  **Inclusion criteria:**  **2. Phase Ia Dose Climbing Study: Patients with advanced HER2-expressing malignant solid tumors who have received prior standard of care. (including surgery, chemotherapy, radiotherapy, or biological therapy) Patients who fail or are unable to receive standard of care or do not receive standard of care;**  a HER2 overexpression: IHC3+, IHC2+/FISH+, or FISH+  b HER2 low expression: IHC1+, IHC2+ but FISH-  **Phase Ib Dose Expansion Study: Histologically or cytologically confirmed HER2-high expression patients with advanced breast cancer who had received at least 2 prior lines of treatment and had failed anti-HER2 therapy, as follows:**  a HER2 overexpression: IHC3+, IHC2+/FISH+, or FISH+;  b Patients with advanced breast cancer who have failed prior anti-HER2 therapy and have received at least 2 lines of therapy, with postoperative adjuvant therapy such as disease progression during treatment and within 12 months after the end of treatment as one line of treatment;  c Evidence of disease progression or intolerable toxicity confirmed by the investigator or documented in medical history prior to enrollment;  d Patients can be enrolled by providing a written HER2 test report from the local laboratory, and patients without a HER2 test report must provide sufficient paraffin sections or fresh tumor tissue specimens to the study center or central laboratory for confirmation. | **Page 12, 45**  **Inclusion criteria:**  **2. Phase Ia Dose Climbing Study: Patients with advanced HER2-expressing malignant solid tumors who received prior standard of care. (including surgery, chemotherapy, radiotherapy, or biological therapy) Patients who fail or are unable to receive standard treatment or no standard treatment;**  a HER2 expression: IHC3+, or IHC2+/FISH+  b HER2 expression: IHC1+, or IHC2+/FISH-  **Phase Ib dose-expansion study: Histologically or cytologically confirmed HER2-positive patients with advanced breast cancer who have received at least 2 prior lines of treatment and who have failed anti-HER2 therapy, as follows:**  a HER2 positive: IHC3+, IHC2+/FISH+;  b Patients with advanced breast cancer who have failed prior anti-HER2 therapy and have received at least 2 lines of treatment, with postoperative adjuvant therapy, such as disease progression during treatment and within 12 months after the end of treatment, can be considered as one line of treatment;  c Evidence of disease progression or intolerable toxicity confirmed by the investigator or documented in medical history prior to enrollment;  d Patients can provide the HER2 test report issued by the pathology department of the medical institution within 5 years as the basis for enrollment, and provide sufficient paraffin section or fresh tumor tissue specimens to the central laboratory for confirmation (if have). | As above. |
| **Pages 12-13, 45**  **Inclusion criteria**  5. Patients with adequate organ and bone marrow function: absolute neutrophils ≥1.0×109/L; hemoglobin ≥ 90 g/L (no red blood cell transfusion within 14 days); Platelet ≥100×109/L; Serum total bilirubin is less than or equal to 1.5 x upper limit normal (ULN) and less than or equal to 3.0 x ULN in patients with Gilbert's syndrome. Aspartate aminotransferase (AST), alanine aminotransferase (alanine aminotransferase, ALT) ≤ 2.5×ULN; In patients with liver metastases, AST and ALT should be less than or equal to 5×ULN. Creatinine < 1.5 x ULN and creatinine clearance **≥ 60 mL/min** (calculated by the Cockroft-Gault equation); Blood potassium ≥3.5 mmol/L; albumin ≥ 3 g/dL; Known left ventricular ejection fraction (LVEF) >50%; Urinary protein ≤ 1 + or 24-hour urinary protein quantitation < 1.0 g; | **Pages 12-13, 46**  **Inclusion criteria**  5. Patients have sufficient organ and bone marrow functions: absolute neutrophils ≥1.0×109/L (no white-increasing drugs were used within 7 days); hemoglobin ≥ 90 g/L (no red blood cell transfusion within 14 days); Platelet ≥ 100 x 109/L (no platelet-enhancing drugs were used within 7 days); Serum total bilirubin is less than or equal to 1.5 x upper limit normal (ULN) and less than or equal to 3.0 x ULN in patients with Gilbert's syndrome. Aspartate aminotransferase (AST), alanine aminotransferase (alanine aminotransferase, ALT) ≤ 2.5×ULN; In patients with liver metastases, AST and ALT should be less than or equal to 5×ULN. Creatinine < 1.5 x ULN and creatinine clearance **≥ 50 mL/min** (calculated by the Cockroft-Gault equation); Blood potassium ≥3.5 mmol/L; albumin ≥ 3 g/dL; Known left ventricular ejection fraction (LVEF) >50%; Urinary protein ≤ 1 + or 24-hour urinary protein quantitation < 1.0 g; | Better protection of subject rights and interests |
| **Pages 13 and 45**  **Inclusion criteria:**  7. Male or female patients of childbearing potential must agree to use effective methods of contraception, such as double barrier methods, condoms, oral or injectable contraceptives, intrauterine devices, etc., during the study and **within 30 days** of the last study medication; | **Page 13, 46**  **Inclusion criteria**  7. Male or female patients of childbearing potential must agree to use effective contraceptive methods, such as double barrier methods, condoms, oral or injectable contraceptives, intrauterine devices, etc., during the study and **within 3 months** of the last study medication; | As above. |
| **Pages 13 and 45**  **Exclusion criteria:**  1. received chemotherapy, targeted therapy, radiotherapy, etc. within 14 days or 5 half-life periods (whichever is shorter) before starting administration ; received major surgery, tumor immunotherapy, and monoclonal antibody therapy within 4 weeks before starting administration; | **Pages 13 and 46**  **Exclusion criteria:**  1. received Chemotherapy, small molecule targeted drug therapy, radiotherapy within 14 days or 5 half-lives (whichever is shorter) before starting administration; received major surgery, tumor immunotherapy, and macromolecular monoclonal antibody therapy within 4 weeks before starting administration; | Clearer distinction between targeted and monoclonal antibody drugs |
| **Pages 13 and 45**  **Exclusion criteria:**  5. Uncontrolled stable diabetic patients. (Patients with stable insulin or other antidiabetic regimens, blood glucose stably controlled, were allowed to enroll) | **Pages 13 and 46**  **Exclusion criteria:**  5. Uncontrolled stable pleural effusion, ascites and pericardial effusion; | Better protection of subject rights and interests |
|  |  |  |
| **Pages 13 and 46**  **Exclusion criteria:**  9. with one of the following Cardiac function and diseases:   1. QTc > 470 ms: average calculated by three times 12-lead electrocardiogram (ECG) according to the instruments’ QTc formula during the screening period; 2. ≥Grade 3 Congestive heart failure with New York Heart Association (NYHA) classification; 3. Clinically significant arrhythmias, including but not limited to complete left bundle branchblock, II^。^ AV block. | **Pages 13 and 47**  **Exclusion criteria:**  9. Cardiac function and disease are one of the following:  a. QTc > 470 ms: average calculated by three times 12-lead electrocardiogram (ECG) according to the instruments’ QTc formula during the screening period;  b. ≥Grade 3 Congestive heart failure with New York Heart Association (NYHA) classification;  c. Clinically significant arrhythmias, including but not limited to complete left bundle branchblock, II^。^AV block  **d. History of myocardial infarction within 6 months.** | Better protection of subject rights and interests |
| **Pages 14, 46**  **Exclusion criteria:**  12. Clinically significant active bacterial, fungal or viral infections, including hepatitis B. (HBV surface antigen positive and HBV DNA over 1000 IU/ml) or Hepatitis C (HCV RNA positive), Human Immunodeficiency Virus Infection (HIV positive); | **Pages 14, 47**  **Exclusion criteria**  12. Clinically significant active infection, including but not limited to hepatitis B. (HBV surface antigen positive and HBV DNA over 1000 IU/ml) or Hepatitis C (HCV RNA positive), Human Immunodeficiency Virus Infection (HIV positive); | Clearer description |
| **Pages 14, 46**  **Exclusion criteria:**  **Added one criteria** | **Pages 14, 47**  **Exclusion criteria**  13 Any other malignancy diagnosed within 3 years prior to participation in the study, other than radically treated early malignancies (carcinoma in situ or stage I), such as adequately treated basal or squamous cell skin cancer or cervical carcinoma in situ; | Better protection of subject rights and interests |
| **Page 14**  **Study Drug and Dosage:**  **Stage Ib: 2.3 mg/kg** | **Page 14**  **Study Drug and Dosage:**  **Phase Ib: 2.3 mg/kg intravenously every 3 weeks** | Clearer description |
| **Pages 14, 41, 63**  **Safety Evaluation**  These included monitoring and recording of AEs and SAEs, protocol-defined laboratory tests (including routine blood, chemistry, and urine tests), 12-lead ECG, and vital signs. The severity will be evaluated according to the NCI-CTCAE Version 5.0.‑ | **Pages 14, 42, 62**  **Safety Evaluation**  It mainly included monitoring and recording of AEs and SAEs, protocol-specified laboratory tests (including routine blood, chemistry, and urine tests), 12-lead ECG, ophthalmological examination, and vital sign. The severity will be evaluated according to the NCI-CTCAE Version 5.0. | Clearer description |
| **Pages 16, 71**  **Pharmacokinetic Evaluation:**  Stage Ib  Blood will be collected for Pop PK analysis within 60 minutes prior to D1 dosing in each treatment cycle. | **Pages 16, 71**  **Pharmacokinetic Evaluation:**  Intensive PK blood sampling is planned for approximately 12 patients at RP2D doses in Phase Ia. The rest refer to the Phase Ib procedure for PK sparse blood sampling only. | Better protection of subject rights and interests |
| **Page 16**  **Sample size:**  The number of effective cases required for stage Ia is estimated to be about 21-42 cases.  The expected number of extended cases in Phase Ib is approximately 50, and the expected observed ORR is 50%, with a 95% confidence interval (35.5-64.5%). | **Page 17**  **Sample size**  The number of effective cases required for stage Ia is estimated to be about 150;  The expected number of patients enrolled in Phase Ib is approximately 50 and the expected observed ORR is 50% with a 95% confidence interval (35.5-64.5%). | Modified based on the current enrollment. |
| **Pages 17 and 75**  **Efficacy Analysis:**  **Phase Ia**  Confirmed ORR will be calculated and Clopper Pearson confidence intervals of 90% will be calculated in the Efficacy and Safety Analysis Sets. Survival analysis will be performed using the Kaplan-Meier method for PFS, DOR, and OS, and descriptive analysis will be performed for 1-year OS rate and DCR based on the investigator's assessment.‑  **Stage Ib**  Confirmed ORR and 95% ClopperPearson confidence intervals will be calculated for the efficacy and safety analysis sets, respectively.‑ | **Pages 18, 74**  **Efficacy Analysis:**  **Phase Ia**  The confirmed ORR will be calculated and the Clopper Pearson confidence interval of 90% will be calculated in the efficacy evaluable analysis set. Survival analysis will be performed using the Kaplan-Meier method for PFS, DOR, and OS, and descriptive analysis will be performed for 1-year OS rate and DCR based on the investigator's assessment.‑  **Stage Ib**  Confirmed ORR will be calculated separately for the IRC and investigator assessments and a 95% Clopper Pearson confidence interval will be calculated in the efficacy evaluable analysis set. | Adjust Analysis Set |
| **Pages 18, 76**  **Interim Analysis:**  **Stage Ib**  An interim analysis using Bayesian posterior probability will be conducted when approximately 20 patients have completed 2 tumor assessments. If the predicted Pr (ORR < 20%) is >80%, i.e. fewer than 6 responses are observed in 20 evaluable patients, then an 80% probability of drug ORR being lower than standard of care is considered and early discontinuation of the cohort may be considered, otherwise enrollment will continue to approximately 50 patients. Bounds will be adjusted based on the actual number of people in the Efficacy Analysis Set. The interim analysis margin is non-binding and the sponsor will make the final decision by considering the safety and effectiveness data. | **Page 18, 75**  **Interim Analysis:**  **Stage Ib**  An interim analysis will be conducted as the data accumulates and the sponsor will consider the safety and effectiveness data to decide whether to continue the trial. | Adjusted based on the development strategy and plan. |
| **Page 41**  **3.1 Overall Study Design**  Phase Ib included patients with advanced breast cancer with high HER2 expression and failure of anti-HER2 therapy who had received at least 2 prior lines of treatment. Dosage and frequency of administration in Phase Ib will be based on RP2D obtained in Phase Ia. | **Page 42**  **3.1 Overall Study Design**  Phase Ib included HER2-positive patients with advanced breast cancer who had failed anti-HER2 therapy and had received at least 2 prior lines of therapy. The dose and frequency for Phase Ib was 2.3 mg/kg q3w. | Adjusted based on the development strategy and plan. |
| **Page 48**  **5.1 Phase Ia**   1. **Screening/Baseline Period (-28 to - 1 day)**  - Past medical history. (Collection of all past medical history, except for this indication, starting before the signing of the ICF, and considered relevant to the study). - Prior tumor history: including date of tumor diagnosis, histopathological classification and grade, ER status (including ER positive rate), PR status (including PR positive rate), HER2 status, Ki-67 proliferation index, clinical stage, presence of metastasis and site of metastasis, Keywords only bone lesions; performance status; Time from diagnosis to relapse, indication of chemotherapy, start/end date of prior treatment regimen, availability of targeted therapy and type of treatment (adjuvant therapy, neoadjuvant therapy, late therapy), number of lines of advanced therapy and treatment regimen, best treatment assessment, and disease progression date; The history of radiotherapy should include start/end date, site of radiotherapy. Previous clinically significant procedures. (e.g., invasive procedures for diagnosis or treatment such as bronchoscopy, biopsy, etc.) To be recorded on the eCRF, including start and end dates, operation name and site. - Tumor tissue sections or fresh tissues were obtained for IHC staining and/or FISH gene amplification testing. (Previous HER2 status reports can be used as the basis for enrollment, and tissue specimens without HER2 status reports prior to enrollment must be sent to the site or central laboratory for confirmation). - Blood chemistry tests included total protein, albumin, blood glucose, total cholesterol, low density lipoprotein, high density lipoprotein, triglyceride, urea, creatinine, alkaline phosphatase, lactate dehydrogenase, total bilirubin, direct bilirubin, indirect bilirubin, AST, ALT, Calcium, phosphorus, magnesium, potassium, sodium, chlorine, serum amylase, etc. - Urine routine includes specific gravity, PH, urine sugar, protein, urine white blood cells, urine red blood cells and ketone bodies. If positive urine protein is grade 2 or more, a 24-hour quantitative urine protein test is required. | **Page 49**  **5.1 Phase Ia**   1. **Screening/Baseline Period (-28 to - 1 day)**  - Past medical history. (Collection of all prior medical history starting prior to the signing of the ICF except for this indication). - Prior tumor history: including the date of tumor diagnosis, histopathological classification and grade, ER status (including ER positive rate), PR status (including PR positive rate), HER2 status, Ki-67 proliferation index, clinical stage, presence and site of metastasis, and performance status; Time from diagnosis to relapse, history of chemotherapy, start/end date of prior treatment regimen; The availability of targeted therapy and type of treatment (adjuvant, neoadjuvant, advanced therapy), number of lines of advanced therapy and treatment regimen, best treatment assessment, and date of disease progression; The history of radiotherapy should include start/end date, site of radiotherapy. Previous clinically significant procedures. (e.g., invasive procedures for diagnosis or treatment such as bronchoscopy, biopsy, etc.) To be recorded on the eCRF, including start and end dates, operation name and site. - Tumor tissue sections or fresh tissues were obtained for IHC staining and/or FISH gene amplification testing. (Patients can provide the HER2 test report issued by the pathology department of the medical institution within 5 years as the basis for enrollment, and provide sufficient paraffin sections or fresh tumor tissue specimens (if any) sent to central laboratory for confirmation). - Blood chemistry tests included total protein, albumin, blood glucose, total cholesterol, low density lipoprotein, high density lipoprotein, triglyceride, urea, creatinine, alkaline phosphatase, lactate dehydrogenase, total bilirubin, direct/indirect bilirubin, AST, ALT, Calcium, phosphorus, magnesium, potassium, sodium, chlorine, serum amylase, etc. - Urine routine includes specific gravity, PH, urine sugar, protein, urine white blood cells, urine red blood cells and ketone bodies. If proteinuria is 2 + or more, a 24-hour quantitative urine protein test is required.   **Ophthalmological examination: visual acuity and slit lamp examination.**  Tumor Assessment: Tumors were evaluated according to RECIST 1.1. CT or MRI scans of the chest, abdomen, pelvis, and skull should be performed at screening. | Increase ophthalmic examination to better protect the rights and interests of subjects. |
| **Pages 49 - 50**  **5.1 Phase Ia**  **Dosing Phase Ia:**   - Laboratory tests: including routine blood, urine, and blood chemistry tests, should be performed within 7 days before the first administration day. (If the relevant laboratory tests for the screening period are still within the time window, the values for the screening period can be used.) - 12-lead ECG, repeated three times, approximately 5 minutes apart, before the first dose. If the test results within 7 days prior to Day 1 are available, it is not necessary to repeat the test before the first dose on Day 1. - Heart ultrasound. - Cardiac function NYHA classification. - Blood sampling for PK and immunogenicity (see Attachment 1). | **Pages 50 - 51**  **5.1 Phase Ia**  **Dosing Phase Ia:**   - Laboratory tests: routine blood tests, routine urine tests, and blood chemistry tests should be performed within 7 days prior to administration. (If the relevant laboratory tests for the screening period are still within the time window, the values for the screening period can be used.) - A 12-lead ECG, repeated three times, approximately 5 minutes apart, before the first dose. If the test results available within 7 days prior to dosing, it is not necessary to repeat the test prior to dosing. - **Cardiac ultrasound (as clinically required).** - **Cardiac function NYHA classification (as clinically required).** - **Ophthalmological examination: visual acuity and slit lamp examination, results obtained within 7 days prior to dosing in each cycle. (C1D1 does not need to be repeated if the screening test results are within this time window).**   Blood sampling for PK and immunogenicity (see Attachment 1). Intensive PK blood sampling is planned for approximately 12 patients at the RP2D dose in Phase Ia. The rest of the reference Phase Ib procedures will be PK sparse blood sampling only. | Better protect the rights and interests of subjects. |
| **Page 50**  **5.1 Phase Ia:**  **3) End of treatment:**   - Laboratory tests, including routine blood tests, routine urine tests, and blood chemistry. - Coagulation tests. - Cardiac ultrasound examination - Cardiac function NYHA classification. | **Page 51**  **5.1 Phase Ia:**  **3) End of treatment:**   - Laboratory tests, including routine blood tests, routine urine tests. (urinary protein 2 + for 24-hour protein quantification), and blood biochemistry. - Coagulation test: as clinically required. - **Ophthalmological examination: visual acuity and slit lamp examination.** - Cardiac ultrasound: as clinically required. - Cardiac function NYHA classification: according to clinical need. | Better protect the rights and interests of subjects. |
| **Page 50**  **5.1 Phase Ia**  **4) Follow-up for 30 days after the end of treatment**   - Laboratory tests, including routine blood tests, routine urine tests, and blood chemistry. - Cardiac ultrasound examination - Cardiac function NYHA classification | **Pages 51 - 52**  **5.1 Phase Ia**  **4) Follow-up for 30 days after the end of treatment**   - Laboratory tests, including routine blood tests, routine urine tests. (urinary protein 2 + for 24-hour protein quantification), and blood biochemistry. - **Ophthalmological examination: visual acuity and slit lamp examination as clinically required.** - Cardiac ultrasound: as clinically required. - Cardiac function NYHA classification: according to clinical need. | Better protect the rights and interests of subjects. |
| **Pages 52 - 53**  **5.2 Phase Ib**   1. **Ib dosing phase**  - Laboratory tests (if results available within 7 days prior to Day 1, it is not necessary to repeat the test before the first dose on Day 1;). - 12-lead ECG. (Repeated three times, approximately 5 minutes apart, during the Screening period and before the first dose). If the test results within 7 days prior to Day 1 are available, it is not necessary to repeat the test before the first dose on Day 1. - Ophthalmic examination: visual acuity and slit lamp examination, results obtained within 3 days prior to dosing in each cycle. - Blood sampling for PK and immunogenicity (see Attachment 1). | **Page 54**  **5.2 Phase Ib**   1. **Ib dosing phase**  - Laboratory tests (repeated prior to dosing is not required if results from tests within 7 days prior to dosing are available). - 12-lead ECG. (Repeated three times, approximately 5 minutes apart, during the screening period and before the first dose). If the test results available within 7 days prior to dosing, it is not necessary to repeat the test prior to dosing. - **Ophthalmological examination: visual acuity and slit lamp examination, results obtained within 7 days prior to dosing in each cycle. (C1D1 does not need to be repeated if the results of the screening period are within this time window).** - Blood sampling for PK and immunogenicity (see Attachment 1). | More in line with clinical practice and protect subject's rights and interests |
| **Page 53**  **5.2 Phase Ib**  **3) End of treatment**   - Laboratory tests - Coagulation tests. | **Page 54**  **5.2 Phase Ib**  **3) End of treatment**   - Laboratory tests, including routine blood tests, routine urine tests. (urinary protein 2 + for 24-hour protein quantification), and blood biochemistry. - Coagulation test: as clinically required. | Better protect the rights and interests of subjects. |
| **Page 54**  **5.2 Phase Ib**  **4) Follow-up for 30 days after the end of treatment**   - Laboratory tests. - Ophthalmological examination: visual acuity and slit lamp examination. | **Page 55**  **5.2 Phase Ib**  **4) Follow-up for 30 days after the end of treatment**   - Laboratory tests: including routine blood tests, routine urine tests. (urinary protein 2 + for 24-hour protein quantification), and blood biochemistry. - Ophthalmological examination: visual acuity and slit lamp examination as clinically required. | Better protect the rights and interests of subjects. |
| **Page 56**  **6.7 Medication Methods**  (2) Dose calculated according to the patient's body weight as specified in the protocol:  FS-1502 lyophilized powder: Take one piece of FS-1502 lyophilized powder, extract 6mL sterilized water for injection with a 10mL syringe and dissolve it into FS-1502 solution with a concentration of 5mg/mL, slowly rotate until the powder is not completely dissolved. The appropriate FS-1502 solution was drawn from the dose calculated based on the patient's body weight and added to 100 mL of 0.9% sodium chloride injection for intravenous drip, and the infusion was completed within 60-90 minutes.  (3) If the patient's body weight changes by more than 10% from baseline, the dose will need to be recalculated.  (4) If infusion-related reactions (IRRs) occur, suspend intravenous drips or reduce the rate of intravenous drips for no more than 30 minutes, and administer antihistamine or hormone therapy as clinically indicated. Any infusion-related adjustments that occur during the IV drip will be recorded in the eCRF. | **Page 57**  **6.7 Method of medication**  (2) Dose calculated based on patient body weight as specified in the protocol:  FS-1502 lyophilized powder: Take one piece of FS-1502 lyophilized powder, use a 10mL syringe to extract 6mL sterilized water for injection and dissolve it into 5mg/mL FS-1502 solution, slowly rotate until the powder is not completely dissolved. Draw the corresponding volume of FS-1502 solution according to the patient's body weight calculated, and round off the second decimal place (e.g., 0.96 ml is taken as 0.9 ml), add it into 100 mL of 0.9% sodium chloride injection, intravenous drip, Instillation is completed within 60-90 minutes.  (3) If the patient's body weight changes by more than 10% from baseline (C1D1), the dose will be recalculated.  (4) If infusion-related reactions (IRRs) occur, suspend intravenous drips or reduce the rate of intravenous drips for no more than 30 minutes, and administer antihistamine or hormone therapy as clinically indicated. Any infusion-related adjustments that occur during the IV drip will be recorded in the eCRF. | Standardize the calculation method of dosage |
| **Page 58**  **6.12.2 QTc Prolongation**  Grade 4 QTc prolongation occurred. (defined as QTc ≥ 501 ms or > 60 ms increase from baseline and torsades, polymorphic ventricular tachycardia, or severe asymptomatic arrhythmia), FS-1502 treatment must be discontinued. Serum potassium and magnesium levels may be monitored and corrected to normal levels, if required. In the event of QTc ≥500 ms, an electrocardiogram (ECG) should be performed again within 1 hour to confirm QTc interval ≥500 ms and to assess the use of concomitant medications. Once Grade 4 QTc prolongation is confirmed, the administration of FS-1502 should be discontinued permanently and the patient should be hospitalized for close cardiac monitoring until the QT interval normalizes or cardiology consultation and adequate treatment is initiated. In other cases, the dose modification was performed according to the dose modification protocol.  If the patient's QTc interval returns to normal, another dose level may be reduced at the discretion of the investigator and the cardiologist. | **Page 59**  **6.12.3 QTc prolongation**  Withhold FS-1502 treatment if QTc ≥ 501 ms or if there is detrosade, polymorphic ventricular tachycardia, or severe asymptomatic arrhythmia.  If QTc is 501 ms or more, the average of two electrocardiograms (ECGs) should be re-examined within 1 hour to confirm the QTc interval is 501 ms or more, and serum potassium and magnesium ion levels should be monitored and corrected to normal. To assess if it was caused by the patient's concomitant medication and if the concomitant medication could be adjusted. When the patient's QTc interval recovers to Grade 1 or less, the dose level may be reduced by one at the discretion of the investigator and the cardiologist if QTc interval prolongation is considered related to FS-1502.  In the event of torsional ventricular tachycardia, polymorphic ventricular tachycardia, or severe asymptomatic arrhythmias, FS-1502 administration should be permanently discontinued and hospitalization for close cardiac monitoring should be carried out. Until the QT interval normalizes or cardiology consultation and adequate treatment is taken.  In other cases, the dose modification protocol was followed. | Clearer treatment of QTc prolongation |
| **Page 60**  **6.12.8 Elevated AST/ALT**  In the event of a Grade 3 ALT/AST increase, dosing was withheld until resolution to ≤ Grade 1 or Baseline. If recovered within 7 days, resume treatment at the same dose level. If recovery occurs between 7 days and 2 cycle times, treatment is continued by lowering to the next studied dose level after recovery. If there is no recovery within 2 cycles (42/56 days), the treatment is discontinued permanently. If Grade 3 ALT/AST elevations recur, hold until resolution to Grade 1 or less or baseline (If not recovered within 7 days, discontinue the drug permanently). In the event of > Grade 3 ALT/AST elevations, assessment is required:  - Detailed medical history: such as alcohol, drugs, nutrition, family history, sexual history, travel history, exposure to jaundice patients, surgery, blood transfusion, history of liver disease or allergic diseases and work environment.  - Physical examination and consideration of the possibility of tumor liver metastases.  - Liver imaging (e.g., biliary tract). | **Page 61**  **6.12.9 Elevated AST/ALT**  In the event of a Grade 3 ALT/AST increase, dosing was withheld until resolution to ≤ Grade 1 or baseline. In the event of > Grade 3 ALT/AST elevations, the assessment is required:  - Detailed medical history: such as alcohol, drugs, nutrition, family history, sexual history, travel history, exposure to jaundice patients, surgery, blood transfusion, history of liver disease or allergic disease and work environment.  - Physical examination and consideration of the possibility of tumor liver metastasis.  - Liver imaging. | keep consistent with the clinical practice. |
| **Page 61**  **6.12.11 Ocular Toxicity**  Monitor visual acuity and slit lamp results closely during medication, and in the event of ocular toxicity, manage according to the dosage adjustment principles described in the table below. For early prophylaxis, use preservative-free artificial tear drop eyes at the start of treatment, throughout the course of treatment, and 30 days after discontinuation.  Ocular toxicity grade was determined according to the NCI CTCAE 5.0 grading, and treatment drugs and outcomes were recorded.   \| **NCI-CTCAE V5.0** \| **Dose modification plan** \| \| --- \| --- \| \| **Ocular Toxicity** \| \| \| **xerophthalmia** \| \| \| Level 1 \| - Maintain original dose and continue treatment, observe closely - Preservative-free artificial tears are recommended, and the frequency can be increased appropriately. \| \| Level 2 \| - Withhold until grade 1 recovery, use original dose - Use preservative-free artificial tears, calf serum deproteinized eye drops or recombinant bovine basic fibroblast growth factor eye drops \| \| Level 3 \| - Dose delayed until ≤ Grade 1, reduce by 1 dose - Preservative-free artificial tears, calf serum deproteinized eye drops or recombinant bovine basic fibroblast growth factor eye drops, ophthalmology specialist \| \| Level 4 \| - Dose was delayed until ≤ Grade 1, and after comprehensive benefit/risk assessment, the dosage was reduced and continued - Preservative-free artificial tears, calf serum deproteinized eye drops or recombinant bovine basic fibroblast growth factor eye drops, ophthalmology specialist \| \| keratitis \| \| \| superficial punctate keratitis \| - Continue treatment with original dose and observe closely \| \| confluent superficial keratitis \| - Dose delayed until ≤ Grade 1, reduce by 1 dose - Discontinue treatment if recurrence \| \| Ulcerative keratitis or perforation \| - Discontinuation of treatment \| \| Conjunctivitis or other ocular adverse events \| \| \| Level 1 \| - Continue treatment with original dose and observe closely \| \| Level 2 \| - Dose was delayed until Grade ≤ 1 after first occurrence, and original dose was resumed - Dose delayed until ≤ Grade 1 after reoccurrence, reduce by 1 dose - Discontinue treatment at third occurrence \| \| Grade 3-4 \| - Discontinuation of treatment \| | **Page 62**  **6.12.12 Ocular Toxicity**  For details, see Attachment 7 FS-1502 Ocular Adverse Event Risk Management Plan | A risk management plan for ocular adverse events was developed to protect the rights and interests of patients. |
