# Supplementary material for: HER2-targeting antibody drug conjugate FS-1502 in HER2-expressing metastatic breast cancer: a phase 1a/1b trial
Source: Nat Commun. 2024 Jun 17;15:5158. doi: 10.1038/s41467-024-48798-w (PMC11183070; doi:10.1038/s41467-024-48798-w)
Supplement: Supplementary file 2 — Reporting Summary [file 41467_2024_48798_MOESM2_ESM.pdf]

nature portfolio

Corresponding author(s): Binghe Xu

Last updated by author(s): May 6, 2024

Reporting Summary

Nature Portfolio wishes to improve the reproducibility of the work that we publish. This form provides structure for consistency and transparency in reporting. For further information on Nature Portfolio policies, see our Editorial Policies and the Editorial Policy Checklist.

Statistics

For all statistical analyses, confirm that the following items are present in the figure legend, table legend, main text, or Methods section.

n/a	Confirmed	
☐	☒	The exact sample size (*n*) for each experimental group/condition, given as a discrete number and unit of measurement
☒	☐	A statement on whether measurements were taken from distinct samples or whether the same sample was measured repeatedly
☒	☐	The statistical test(s) used AND whether they are one- or two-sided<br>*Only common tests should be described solely by name; describe more complex techniques in the Methods section.*
☒	☐	A description of all covariates tested
☒	☐	A description of any assumptions or corrections, such as tests of normality and adjustment for multiple comparisons
☐	☒	A full description of the statistical parameters including central tendency (e.g. means) or other basic estimates (e.g. regression coefficient) AND variation (e.g. standard deviation) or associated estimates of uncertainty (e.g. confidence intervals)
☒	☐	For null hypothesis testing, the test statistic (e.g. *F*, *t*, *r*) with confidence intervals, effect sizes, degrees of freedom and *P* value noted<br>*Give P values as exact values whenever suitable.*
☒	☐	For Bayesian analysis, information on the choice of priors and Markov chain Monte Carlo settings
☒	☐	For hierarchical and complex designs, identification of the appropriate level for tests and full reporting of outcomes
☒	☐	Estimates of effect sizes (e.g. Cohen's *d*, Pearson's *r*), indicating how they were calculated

Our web collection on statistics for biologists contains articles on many of the points above.

Software and code

Policy information about availability of computer code

Data collection	Medidata Classic Rave System (Version: Medidata Classic Rave® 2023.1.1)
Data analysis	Statistical analysis was performed using SAS version 9.4, and PK data analysis was performed using WinNonlin software.

For manuscripts utilizing custom algorithms or software that are central to the research but not yet described in published literature, software must be made available to editors and reviewers. We strongly encourage code deposition in a community repository (e.g. GitHub). See the Nature Portfolio guidelines for submitting code & software for further information.

Data

Policy information about availability of data

All manuscripts must include a data availability statement. This statement should provide the following information, where applicable:

- Accession codes, unique identifiers, or web links for publicly available datasets
- A description of any restrictions on data availability
- For clinical datasets or third party data, please ensure that the statement adheres to our policy

The de-identified participant data that support the findings of this study are available from the corresponding author by contacting xubinghe@medmail.com.cn for research purposes and will be responded to within 4weeks. Source data are provided with this paper. The study protocol can be found in the Supplementary Information as Supplementary Note 5. All remaining data can be found in the Article, Supplementary and Source data files.

Research involving human participants, their data, or biological material

Policy information about studies with human participants or human data. See also policy information about sex, gender (identity/presentation), and sexual orientation and race, ethnicity and racism.

Reporting on sex and gender	Sex and/or gender was not considered in the study design as this trial investigated a treatment for breast cancer and it was anticipated that the majority of or all patients would be females. Sex and/or gender of participants was determined based on self-report. No sex- and gender-based analyses have been performed in this study, the analysis was based on patient disease status.
Reporting on race, ethnicity, or other socially relevant groupings	There were no race, ethnicity, or other socially relevant groupings in this study.
Population characteristics	For the whole study, the median age was 52.0 years (range 27-76), most patients were female (n = 146, 97.3%), and 145 (96.7%) patients were diagnosed with breast cancer; two (1.3%) had lung cancer, and one (0.7%) each had ampullary cancer, submandibular gland malignant tumor, and gastric cancer. For patients with breast cancer, 112 (77.2%) were HER2-positive and 32 (22.1%) had tumors with HER2-low expression. For non-breast cancer patients, two (40%) were HER2 IHC 2+ and three (60%) were HER2 IHC 3+. The most common metastatic sites were lymph nodes (60.7%), lung (54.0%), bone (42.0%) and liver (42.0%); 11 (7.3%) had brain metastases. Patients with ≥3 metastatic sites accounted for 59.3% (n = 89) across all dose groups. Among HER2-positive patients treated at the RP2D, almost all patients (99%) had visceral metastases. Among all patients enrolled, 88.7% received a median of 3 lines of previous therapy and for those patients enrolled at the RP2D, 93.3% received ≥ to 2 previous lines of therapy. All patients with HER2-positive breast cancer had previously received anti-HER2 therapy, among which the most common treatments were trastuzumab (n = 107, 95.5%), pyrotinib (n = 75, 67.0%) and pertuzumab (n = 38, 33.9%). A small number of patients had also received previous treatment with T-DM-1 (n=6, 5.4%). At the data cutoff date, 97 (64.7%) patients had discontinued treatment, most due to disease progression (n = 77, 79.4%; Fig. 1).
Recruitment	Eligible participants were enrolled based on their eligibility without selection at nine study sites in China.
Ethics oversight	This study was approved by relevant institutional review board and/or ethics committee at nine study sites in China. National Cancer Center/Cancer Hospital Chinese Academy of Medical Sciences and Peking Union Medical College, Beijing, China; Sir Run Run Shaw Hospital, Zhejiang university School of Medicine, Zhejiang, China; Jilin Cancer Hospital, Jilin, China; The Fourth Hospital of Hebei Medical University, Hebei, China; Cancer Hospital Chinese Academy of Medical Sciences, Shenzhen Center, Shenzhen, China; Henan Cancer Hospital, Zhengzhou, China; Tianjin Medical University Cancer Institute and Hospital, Tianjin, China; Sun Yat-sen University Cancer Center, Guangzhou, China; Meizhou People's Hospital, Meizhou, China.

Note that full information on the approval of the study protocol must also be provided in the manuscript.

Field-specific reporting

Please select the one below that is the best fit for your research. If you are not sure, read the appropriate sections before making your selection.

☒ Life sciences ☐ Behavioural & social sciences ☐ Ecological, evolutionary & environmental sciences

For a reference copy of the document with all sections, see nature.com/documents/nr-reporting-summary-flat.pdf

Life sciences study design

All studies must disclose on these points even when the disclosure is negative.

Sample size	The dose-escalation study enrolled patients with a 3+3 approach, and approximately 92 patients were estimated for enrollment. Approximately 50 patients were estimated for enrollment in the dose-expansion study, with the assumption that the ORR of FS-1502 was 50%, type I error was 0.025 for single side, and the 95% CI was 35.5-65.4%.
Data exclusions	No data were excluded.
Replication	This was a phase 1 trial to determine the tolerability, MTD, RP2D, and antitumor activity of FS1502. Findings from this phase 1 trial will be validated in future clinical trials.
Randomization	This is an open-label, single-arm study.
Blinding	This is an open-label, single-arm study.

Reporting for specific materials, systems and methods

We require information from authors about some types of materials, experimental systems and methods used in many studies. Here, indicate whether each material, system or method listed is relevant to your study. If you are not sure if a list item applies to your research, read the appropriate section before selecting a response.

Materials & experimental systems

n/a	Involved in the study
☒	☐ Antibodies
☒	☐ Eukaryotic cell lines
☒	☐ Palaeontology and archaeology
☒	☐ Animals and other organisms
☐	☒ Clinical data
☒	☐ Dual use research of concern
☒	☐ Plants

Methods

n/a	Involved in the study
☒	☐ ChIP-seq
☒	☐ Flow cytometry
☒	☐ MRI-based neuroimaging

Clinical data

Policy information about clinical studies
All manuscripts should comply with the ICMJEguidelines for publication of clinical research and a completedCONSORT checklist must be included with all submissions.

Clinical trial registration: The study is registered with ClinicalTrials.gov, NCT03944499.

Study protocol: Study protocol is provided and submitted with the manuscript.

Data collection: All patients provided written informed consent before entering the study.
Patient data was collected via electronic case report form system at nine study sites in China.
Patients were enrolled between November 11, 2019, and December 13, 2022. The cutoff date for safety and efficacy data for this study was December 24, 2022, and for pharmacokinetic (PK) data was July 30, 2022.

Outcomes: The primary end points of the dose-escalation part were DLTs (defined as predefined toxicities that occur during the DLT observation period, details are provided in the Supplementary Information), MTD (defined as the maximum dose of <33% of DLT events observed in patients with evaluable DLT events), RP2D (determined based on analysis of PK/PD, safety and efficacy results). Secondary end points included safety end points other than DLTs, such as incidence of TEAEs, SAEs, and TEAEs leading to drug discontinuation/ death. Other secondary end points were ORR (defined as the proportion of patients with confirmed CR and PR according to RECIST version 1.1) assessed by the investigator, PFS (defined as the time from the first dose of study treatment to disease progression or death, whichever occurred first), OS (defined as the time from the initiation of study treatment to death due to any cause), 1-year OS rate (defined as the proportion of patients that survived within 1 year of the initiation of study treatment), DOR (defined as the time from first CR or PR to disease progression or death due to any cause, whichever occurred first), and clinical benefit rate (CBR, defined as the proportion of patients with CR, PR and SD lasting ≥24 weeks according to RECIST version 1.1), PK parameters of FS-1502, total antibody and unconjugated MMAF (including maximum concentration, half-lives, area under the serum concentration-time curves, clearance and accumulation ratio, etc), ADA and the NAb of FS-1502. DCR (defined as the proportion of patients with CR, PR, and SD lasting ≥6 weeks) based on the investigator's assessment was not a prespecified end point, but was also analyzed in the efficacy analysis.
The primary end point of the dose-expansion part was to evaluate the IRC-assessed ORR of patients with HER2-positive breast cancer. Secondary end points included safety (such as incidence of TEAEs, SAEs, TEAEs leading to drug discontinuation, and frequency and cause of death within 30 days after the last dose), PFS, OS, 1-year OS rate, DOR, and CBR, PK parameters of FS-1502, total antibody and unconjugated MMAF, the ADA and the NAb of FS-1502. DCR, not a prespecified end point, was also analyzed in the efficacy analysis.

Plants

Seed stocks: *Report on the source of all seed stocks or other plant material used. If applicable, state the seed stock centre and catalogue number. If plant specimens were collected from the field, describe the collection location, date and sampling procedures.*

Novel plant genotypes: *Describe the methods by which all novel plant genotypes were produced. This includes those generated by transgenic approaches, gene editing, chemical/radiation-based mutagenesis and hybridization. For transgenic lines, describe the transformation method, the number of independent lines analyzed and the generation upon which experiments were performed. For gene-edited lines, describe the editor used, the endogenous sequence targeted for editing, the targeting guide RNA sequence (if applicable) and how the editor was applied.*

Authentication: *Describe any authentication procedures for each seed stock used or novel genotype generated. Describe any experiments used to assess the effect of a mutation and, where applicable, how potential secondary effects (e.g. second site T-DNA insertions, mosiacism, off-target gene editing) were examined.*
